# Supplementary material for: Biomarker-guided duration of Antibiotic Treatment in Children Hospitalised with confirmed or suspected bacterial infection (BATCH): protocol for a randomised controlled trial
Source: BMJ Open. 2022 Jan 25;12(1):e047490. doi: 10.1136/bmjopen-2020-047490 (PMC8796242; doi:10.1136/bmjopen-2020-047490)
Supplement: Supplementary data [file bmjopen-2020-047490supp001.pdf]

Insert NHS Trust / Health Board logo

Biomarker-guided duration of Antibiotic Treatment in Children Hospitalised with confirmed or suspected bacterial infection Trial

Information Sheet for Parents & Guardians

We are inviting you and your child to take part in this research trial run by Doctors at your hospital and scientists at the University of Liverpool, as well as some researchers from Cardiff University, Lancaster University and the University of Southampton. Before you decide if you want your child to take part, we would like you to understand why the research is being done and what it would involve for you and your child.

Please read this information sheet carefully. One of our team can go through the information sheet with you and will answer any questions you have.

What is the study about?

A daily task in hospitals is to assess ill children for the presence or absence of infection. A decision has to be made as to whether to start, stop or change antibiotics. If we can reduce antibiotic usage within hospitals, this would be an important step in combating the spread of hospital superbugs.

We are interested in studying bacterial infections in children and young people admitted to hospital. We will be assessing different markers of bacterial infection in children in hospital, to see if they can help us identify how children and young people are responding to the antibiotics that are being used to treat the infection.

Waldron C-A, *et al. BMJ Open* 2022; 12:e047490. doi: 10.1136/bmjopen-2020-047490

Procalcitonin is a blood test which measures the body's response to bacterial infection, so the level is high if you have a bacterial infection, but levels reduce as you respond to antibiotics.

We will be using procalcitonin to help us decide if a patient is responding to antibiotic treatment or whether the treatment can be stopped safely.

We want to see if the use of a procalcitonin test can safely aid decisions to start, stop or change antibiotics. We are looking to recruit nearly two thousand patients aged under 18 years old to take part in a trial to help us reduce antibiotic usage safely in children and young people admitted to hospital.

We need to assess new tests of infection in a scientific manner to be sure that they improve patient care before being introduced into everyday routine practice.

Why has my child been asked to take part?

Your child's clinician thinks that your child may have a bacterial infection, and has identified that they could be eligible for the BATCH trial.

Does my child have to take part?

Your child does not have to take part in this trial. Participation in the study is entirely voluntary and you and your child are free to refuse to take part or withdraw from the trial at any time without having to give a reason. If you do decide not to take part or to withdraw from the trial, this will not affect the care that your child receives.

What will happen if my child takes part?

If you do decide to take part in the trial your child will be randomised to either have procalcitonin tests or normal care. We will try to do these procalcitonin tests at the same time as routine blood tests are being taken, however, we may need to take an additional blood test at separate time points if routine bloods are not due to be collected or there is not enough routine blood collected to perform the procalcitonin test,

There will be an equal chance of your child having the additional procalcitonin tests or receiving normal care.

Waldron C-A, *et al. BMJ Open* 2022; 12:e047490. doi: 10.1136/bmjopen-2020-047490

Can I choose which part of the trial to be in?

No. To get the most reliable information about how well the procalcitonin test works we must ensure that each patient is given an equal chance of having the additional tests or not, and that this is chosen at random.

During your child's hospital stay

Your child's clinician will manage your child's care as per normal practice, however, if your child is randomised to the intervention group and has the procalcitonin tests, your child's treatment will be guided by the results of these tests. We will study how your child's procalcitonin levels relate to their response to treatment.

We will collect an additional 1ml of blood for this test. We will try to collect this when blood is being taken as part of routine care, however we may need to take an additional blood test at separate time points if routine bloods are not due to be collected or there is not enough routine blood collected to perform the procalcitonin test. All the other tests performed are those which should routinely be performed.

We will collect information on:

- whether infection is present and on the severity of their illness
- costs associated with having a child in hospital with a bacterial infection
- You will also be asked to complete some questionnaires about your child's health status.

If you do not give consent to take part in the study this will not affect the care given to your child.

What will happen to the blood sample?

Your child's samples taken whilst they are being treated for this illness will be tested in accredited laboratories at your hospital. They will be kept safe and will only be identified by a study code.
This may include samples taken when they were admitted which are normally discarded by the laboratory after the routine tests are done.

Your child's blood samples taken whilst they are being treated for this illness may also be stored for future research concerning infections and antibiotic resistance.

Waldron C-A, *et al. BMJ Open* 2022; 12:e047490. doi: 10.1136/bmjopen-2020-047490

The samples will be stored **confidentially** in a secure location in a laboratory at the University of Liverpool. Your child's name will not be stored with the sample and will not be used in any publications relating to use of your child's sample(s). **You may withdraw consent for this storage and future use of your child's sample(s)**. If you withdraw consent, the sample(s) will not be used in any future studies and will be destroyed according to approved practices. We will seek ethical approval if we want to use the samples for any future research. We will not be doing any tests on human DNA now or in the future.

At Day 28 follow-up.

We will contact you 28 days after your child was recruited into the study to find out how they are. This will likely be by telephone, email or post and should take around half an hour of your time. We would also like to look at your child's medical records so that we can collect information on healthcare costs such as prescriptions, visits to the GP and so on. This is to allow us to work out if the additional test to reduce antibiotic use will save money in the NHS in the long run.

Longer term follow up using health data

After the trial, we would like to see how your child is doing in the longer term by using routinely collected health data. This is information that your GP and hospital collect when looking after your child. We will look at this information to explore the long term effects of procalcitonin test versus normal care.

The reason we want to look at this information is to assess your child's well-being and health following the trial. The research team will provide details such as name, NHS number, date of birth, postcode and gender to identify your child to NHS Digital in order for those professionals to provide relevant information about your child's ongoing health. NHS Digital will provide information on how trial participants have used different health services (e.g. how many hospital visits) and for what reasons.

NHS digital will provide data to Cardiff University. No identifiable data will be sent to the database. Instead, a study number will be assigned to each individual and this will be used to join pieces of information together. Data viewed by the research team will not be identifiable. In other words, when we look at your health information, all we will see is a database containing numbers. A Data Manager will use the study number to identify a person and join up the information. We will not know who is who in the database. The data will be held on a secure server and only the research team will have access to it.

Qualitative interview study

A few parents in the trial will be asked by a member of the research team to discuss the experience of their child taking part in the trial and about their child's health. If you agree, a researcher will give you some information about the additional study and what will be required. After you have had time to go through this and ask any questions you might have, you will be asked if you are happy to take part. If so you will be asked to sign a consent form to confirm that you understand this part of the study and agree to participate. If you do not take part this will not affect your involvement in the research or ongoing health care.

What are the possible advantages of taking part?

If the number of days that it is necessary to give antibiotic treatment can be safely reduced, children may benefit from the possibility of fewer days suffering from the known side effects of antibiotic treatment and a reduced risk of building up resistance to antibiotics working in the future (antimicrobial resistance). Your child will continue to receive standard clinical treatment, and their clinicians continue to decide on how long antibiotic treatment is given. By participating in this trial the information we collect about your child will help children/young people in the future.

What are the possible disadvantages and risks of taking part?

Taking part in the trial will mean that we ask you to give up some of your time during your child's hospital stay and at your child's follow-up telephone call. There are no risks for your child. In the event that something does go wrong and your child is harmed during the research and this is due to someone's negligence then you may have grounds for a legal action for compensation against Alder Hey Children's NHS Foundation Trust and The University of Liverpool, but you may have to pay your legal costs. The normal National Health Service complaints mechanisms will still be available.

Will all my information be kept confidential?

Yes, all information collected about you and your child during the course of the trial will be kept strictly confidential. Doctors involved in the care of your child will be informed about your participation in the trial, and will be kept up to date with your child's progress to ensure their safety, but they will not see any answers to trial questionnaires.

Waldron C-A, *et al. BMJ Open* 2022; 12:e047490. doi: 10.1136/bmjopen-2020-047490

Your child's medical records may be examined by the research team but will be kept confidential. Your child's name (or any other identifiable information) will not be given out or appear on any publications.

University of Liverpool and Cardiff University will use your name, date of birth and contact details to contact you about the research study, and make sure that relevant information about the study is recorded for your care, and to oversee the quality of the study. Individuals from University of Liverpool and Cardiff University and regulatory organisations may look at your research records to check the accuracy of the research study. The only people in University of Liverpool and Cardiff University who will have access to information that identifies you/your child will be people who need to contact you to collect more information, take part in a qualitative interview, send you the results of the trial or to audit the data collection process. The people who analyse the information will not be able to identify you and will not be able to find out your name, or contact details.

University of Liverpool and Cardiff University will keep identifiable information about you/your child securely, for a minimum of 10 years after the BATCH trial has finished. This is in line with University of Liverpool policies.

All members of the trial team and regulatory authorities are trained in data protection issues. They are also bound by the terms of the General Data Protection Regulation (GDPR) (EU) 2016/679.

Who is responsible for looking after my information?

University of Liverpool is the sponsor for this study based in the United Kingdom. The University of Liverpool and Cardiff University will be using information from you and/your child's medical records in order to undertake this study and will act as the data controller for this study. This means that we are responsible for looking after your information and using it properly. Your rights to access, change or move your information are limited, as we need to manage your information in specific ways in order for the research to be reliable and accurate. If you withdraw from the study, we will keep the information about you/ your child that we have already obtained. To safeguard your rights, we will use the minimum personally-identifiable information possible.

You can find out more about how we use your information here:
https://www.cardiff.ac.uk/public-information/policies-and-procedures/data-protection

Waldron C-A, *et al. BMJ Open* 2022; 12:e047490. doi: 10.1136/bmjopen-2020-047490

Is my data likely to be used for future research?

When you agree to take part in a research study, the information about your health and care may be provided to researchers running other research studies in this organisation and in other organisations. These organisations may be universities, NHS organisations or companies involved in health and care research in this country or abroad. Your information will only be used by organisations and researchers to conduct research in accordance with the UK Policy Framework for Health and Social Care Research.

This information will not identify you/your child and will not be combined with other information in a way that could identify you. The information will only be used for the purpose of health and care research, and cannot be used to contact you or to affect your care. It will not be used to make decisions about future services available to you, such as insurance.

What if I, or my child do not want to carry on being part of the trial?

You can decide for your child to stop taking part in the trial at any time and without needing to give a reason. If you wish to withdraw, you can contact the trial manager or let the research nurse know next time they contact you. In order for us to understand the reasons why parents withdraw their children from the study, we may ask you why you have decided to withdraw. However, you do not have to give any reasons.

It is usual practice to keep the information we have collected to help draw conclusions about the treatment you received. If however you do not want your information used you may request that it be withdrawn from the study as well.

If you decide to stop taking part, your child's current and future medical care and the legal rights of you and your child will not be affected in any way.

What will happen to the results of the research?

We hope that the results of the trial will improve the care of many patients in the future. At the end of the trial, the results will be published in medical journals and presented at medical conferences. This will allow us to tell other doctors and healthcare professionals across the world about the results of the trial.

Waldron C-A, *et al. BMJ Open* 2022; 12:e047490. doi: 10.1136/bmjopen-2020-047490

Information that identifies you or your child **will not** be presented. We can provide you with information of where to access the published trial data. A copy of the results can be sent to you if you would like.

Who has funded and approved the study?

This trial has been funded by the National Institute for Health Research Health Technology Assessment (NIHR HTA) Programme and is being managed by Cardiff University.

The study has been approved by an independent NHS Research Ethics Committee (REC) North West-Liverpool East (reference: 18/NW/0100). The committee makes sure that the study is conducted ethically to protect the safety, rights, wellbeing and dignity of the patient in line with the requirements of Clinical Trial Regulations.

What if there is a problem?

If your child experiences any side effects or complications as a result of taking part in the trial there are no compensation arrangements over and above the usual routes available to any patient treated within the NHS. If at any point you are unhappy with any aspect of the trial, please advise your child's Clinician or the research team at the Centre for Trials Research, Cardiff University (contact numbers below). If you remain unhappy and wish to formally complain, you can do this through the normal NHS complaints procedure. Taking part in the trial will not affect your legal rights.

What do I need to do now?

If you agree to consider having your child take part in the study please tell your child's clinician whether or not you are happy for your child to take part. If your child is old enough you will probably want to discuss the study with them. The research nurse can help you discuss the study with your child.

Thank you for reading this information sheet and considering participation in this trial. Our team is experienced and dedicated to doing this important trial to the highest international standards, and helping to improve the future care of patients.

Waldron C-A, *et al. BMJ Open* 2022; 12:e047490. doi: 10.1136/bmjopen-2020-047490

BMJ Publishing Group Limited (BMJ) disclaims all liability and responsibility arising from any reliance placed on this supplemental material which has been supplied by the author(s)

Should you have any further questions or require further information about taking part you can contact (during normal working hours):

BATCH Trial Manager
Centre for Trials Research,
Cardiff University,
Heath Park,
Cardiff, CF14 4YS
Tel: 02920 687609
Email: BATCH@cardiff.ac.uk

Please note that this number is only for queries regarding the trial; if you have an urgent medical problem please contact your surgeon/doctor in the normal way.

The Principal Investigator for this site is:-

Insert PI details

The Research Nurse for this site is:-

Insert RN detail

Waldron C-A, *et al. BMJ Open* 2022; 12:e047490. doi: 10.1136/bmjopen-2020-047490
