# Supplementary material for: Biomarker-guided duration of Antibiotic Treatment in Children Hospitalised with confirmed or suspected bacterial infection (BATCH): protocol for a randomised controlled trial
Source: BMJ Open. 2022 Jan 25;12(1):e047490. doi: 10.1136/bmjopen-2020-047490 (PMC8796242; doi:10.1136/bmjopen-2020-047490)
Supplement: Supplementary data [file bmjopen-2020-047490supp002.pdf]

BATCH: Biomarker-guided duration of Antibiotic Treatment in Children Hospitalised with confirmed or suspected bacterial infection Trial

Centre for Trials Research / Canolfan Ymchwil Treialon

Insert NHS Trust /Health Board logo

Site ID | | |

Patient ID | | | |

CONSENT FORM

(Please **initial** each box and sign in full at the bottom of the page)

1. I confirm that I have read and understood the Information Sheet Information Sheet for Parents and Guardians (version 2.1, dated 31/01/2019) for the BATCH trial. I have had the opportunity to consider the information, ask questions and have had these answered satisfactorily. ☐
2. I understand that my child's participation is voluntary and that I am free to withdraw my child at any time, without giving any reason, without my child's normal medical care or legal rights being affected. ☐
3. I understand the trial is randomised and no one can pick which treatment my child receives. I understand that my child will be randomised to have either procalcitonin tests or usual care. If my child is allocated to the treatment arm of the trial their procalcitonin levels will be tested. This may be done as part of routine blood tests or an additional blood sample may need to be taken at separate time points if routine bloods are not due to be collected or not enough routine blood is collected to perform the procalcitonin test. I consent to these tests being performed on the samples collected during my child's illness. I understand that I will not be given the laboratory results. ☐
4. I understand that the research team may be required to look in my child's medical notes or contact my child's GP to ask about my child's health and medications. I give permission for them to do this. ☐
5. I understand that a member of the research team will need to contact me by telephone, email or post to complete a survey about my child's health. I give permission for them to do so. ☐
6. I understand that a member of the research team may need to contact me to carry out an interview about my child's health and experiences in the trial (Qualitative Interviews). I give permission for them to do so. ☐
7. I understand that information collected about my child that is held and maintained by NHS Digital and other central UK NHS bodies, may be collected from my medical records and other health-related records and looked at by the research team and responsible practitioners during the trial. I give permission for these individuals to have access to these records and for them to be used in this research on the understanding that all information will remain confidential. ☐
8. I understand that information collected about my child (including name and address) will be held at the Centre for Trials Research, Cardiff University according to the 2018 General Data Protection Regulation (GDPR) (EU 2016/679). I understand that this information will be kept strictly confidential and that no personal information will be used in the study report or publications. ☐
9. I understand that my child's blood samples will be stored securely for further research. Names and addresses will be removed from all samples stored for research purposes. Further ethical approval will be sought from the Ethics Committee for any future research on these samples. No DNA analysis will be carried out now or in the future. ☐
10. I agree for my child to take part in the above trial. ☐

Name of Child (please print):____

Name of Parent / Guardian (please print):___________________________ Signed:__________Date:___/___/___

Name of Person taking consent (please print):________________________Signed:__________Date:___/___/___

When completed, store White copy in Site File; Green for Medical Notes; Yellow for parent/carer
Please fax a copy to CTR: 02030 095405

Local Researcher: Professor Enitan Carrol, Honorary Consultant in Paediatric Infectious Diseases and Immunology, University of Liverpool and Alder Hey Children's NHS Foundation Trust

BATCH Consent form v2.1 **IRAS: 235042** **Date 31.01.2019**
