# Supplementary material for: Biomarker-guided duration of Antibiotic Treatment in Children Hospitalised with confirmed or suspected bacterial infection (BATCH): protocol for a randomised controlled trial
Source: BMJ Open. 2022 Jan 25;12(1):e047490. doi: 10.1136/bmjopen-2020-047490 (PMC8796242; doi:10.1136/bmjopen-2020-047490)
Supplement: Author's manuscript [file bmjopen-2020-047490.draft_revisions.pdf]

BMJ Open is committed to open peer review. As part of this commitment we make the peer review history of every article we publish publicly available.

When an article is published we post the peer reviewers' comments and the authors' responses online. We also post the versions of the paper that were used during peer review. These are the versions that the peer review comments apply to.

The versions of the paper that follow are the versions that were submitted during the peer review process. They are not the versions of record or the final published versions. They should not be cited or distributed as the published version of this manuscript.

BMJ Open is an open access journal and the full, final, typeset and author-corrected version of record of the manuscript is available on our site with no access controls, subscription charges or pay-per-view fees (http://bmjopen.bmj.com).

If you have any questions on BMJ Open's open peer review process please email info.bmjopen@bmj.com

BMJ Open

Biomarker-guided duration of Antibiotic Treatment in Children Hospitalised with confirmed or suspected bacterial infection (BATCH): Protocol for a randomised controlled trial.

Journal:	*BMJ Open*
Manuscript ID	bmjopen-2020-047490
Article Type:	Protocol
Date Submitted by the Author:	30-Nov-2020

Complete List of Authors:	Waldron, Cherry-Ann; Cardiff University, Centre for Trials Research<br>Thomas-Jones, Emma; Cardiff University, Centre for Trials Research<br>Bernatoniene, Jolanta; Bristol Royal Hospital for Children, Paediatric Infectious Disease Department<br>Brookes-Howell, Lucy; South East Wales Trials Unit,<br>Faust, Saul; University of Southampton, UK, NIHR Wellcome Trust Clincial Research Facility<br>Harris, Debbie; Cardiff University, Centre for Trials Research<br>Hinds, Lucy ; Sheffield Children's NHS Foundation Trust, Paediatrics<br>Hood, Kerenza; Cardiff University,<br>Huang, Chao; Hull York Medical School,<br>Mateus, Ceu; University of Lancaster, Division of Health Research<br>Pallmann, Philip; Cardiff University, College of Biomedical and Life Sciences, Centre for Trials Research<br>Patel, Sanjay; Southampton Children's Hospital<br>Paulus, Stephane; University of Oxford, Paediatrics; University of Liverpool Institute of Infection and Global Health,<br>Peak, Matthew; Alder Hey Children's NHS Foundation Trust, R&D<br>Powell, Colin; Children's Hospital for Wales, Paediatrics<br>Preston, Jenny<br>Carrol, Enitan; University of Liverpool, Institute of Infection, Veterinary and Ecological Sciences

Keywords:	INFECTIOUS DISEASES, Paediatric intensive & critical care < INTENSIVE & CRITICAL CARE, Diagnostic microbiology < INFECTIOUS DISEASES, Public health < INFECTIOUS DISEASES

SCHOLARONE™ Manuscripts

Biomarker-guided duration of Antibiotic Treatment in Children Hospitalised with confirmed or suspected bacterial infection (BATCH): Protocol for a randomised controlled trial.

Waldron CA[1*], Thomas-Jones E[1], Bernatoniene J[2], Brookes-Howell L[1], Faust S.N[3], Harris D[1], Hinds L[4], Hood K[1], Huang C[5], Mateus C[6], Pallmann P[1], Patel S[7], Paulus S[8], Peak M[9], Powell C[10], Preston J[9], Carrol ED[11]

[*]Corresponding author
Dr Cherry-Ann Waldron, Centre for Trials Research, Cardiff University, 7th Floor, Neuadd Meirionnydd, Heath Park, Cardiff, CF14 4YS, Tel: +44(0)29 20687609, Email: waldronc@cardiff.ac.uk

1 Centre for Trials Research, Cardiff University, Cardiff, UK
2 University Hospitals Bristol NHS Foundation Trust
3 University Hospital Southampton NHS Foundation Trust, Southampton, UK
4 Sheffield Children's NHS Foundation Trust, Sheffeld, UK
5 Hull York Medical School, Hull, Univeristy of Hull, UK
6 Division of Health Research, Lancaster Univeristy, Lancaster, UK
7 Faculty of Medicine, University of Southampton, University Hospital Southampton NHS Foundation Trust, Southampton
8 Oxford University Hospitals NHS Foundation Trust, Oxford, UK
9 Alder Hey Children's NHS Foundation Trust, Liverpool, UK
10 Child Health, School of Medicine, Cardiff Univeristy, UK / Sidra Medicine, Doha, Qatar
11 University of Liverpool, Liverpool, UK

Key words: Paedatrics, Bacterial infection, Procalcitonin, Antibiotic duration

For peer review only

ABSTRACT

Introduction

Procalcitonin (PCT) is a biomarker more specific for bacterial infection and responds quicker than other commonly used biomarkers such as C-Reactive Protein, but is not routinely used in the NHS. Studies mainly in adults show that using PCT to guide clinicians may reduce antibiotic use, reduce hospital stay, with no associated adverse effects such as increased rates of hospital re-admission, incomplete treatment of infections, relapse or death. A review conducted for NICE recommends further research on PCT testing to guide antibiotic use in children.[1]

For peer review only - http://bmjopen.bmj.com/site/about/guidelines.xhtml

Methods and analysis

BATCH is a multi-centre, prospective, two-arm, individually RCT with a 28 day follow up and internal pilot. The intervention is a PCT-guided algorithm used in conjunction with best practice. The control arm is best practice alone. We plan to recruit 1942 children, aged between 72 hours and up to 18 years old, who are admitted to hospital and being treated with intravenous antibiotics for suspected or confirmed bacterial infection. Co-primary outcomes are duration of antibiotic use and a composite safety measure. Secondary outcomes include time to switch from broad to narrow spectrum antibiotics, time to discharge, adverse drug reactions, health utility and cost effectiveness. We will also perform a qualitative process evaluation.

Ethics and dissemination

The trial protocol was approved by the HRA and NHS REC (North West Liverpool East REC reference 18/NW/0100). We will publish the results in international peer-reviewed journals and present at scientific meetings.

Trial registration: ISRCTN 11369832

Strengths and limitations of this trial

- First trial to evaluate effectiveness of PCT to guide antibiotic duration in children hospitalised with suspected or confirmed bacterial infection.
- Potential to inform NICE guidelines and impact clinical care, by reducing exposure to antibiotics, leading to a reduction in antimicrobial resistance.
- Potential shift in patient population following a recruitment break due to COVID-19 pandemic will be addressed with sensitivity analyses.

INTRODUCTION

Sepsis is defined as life-threatening organ dysfunction caused by a dysregulated host response to infection.[2] Sepsis causes many non-specific symptoms and signs that can also be caused by a large number of conditions that may or may not be due to infection, and that may or may not require immediate or urgent treatment. Sepsis is usually caused by bacteria, although viral and fungal causes do occur. The problem for clinicians is the difficulty in

For peer review only - http://bmjopen.bmj.com/site/about/guidelines.xhtml
For peer review only

distinguishing bacterial sepsis from other conditions presenting with similar non-specific signs and symptoms. Prompt administration of antibiotics reduces mortality by half,[3] but indiscriminate antibiotic use unnecessarily increases antimicrobial resistance (AMR), resulting in increased costs in hospitalised patients,[4 5] which represents a significant resource burden in the NHS. Not all children admitted with bacterial infection will meet the criteria for sepsis, but they could still have serious infection, requiring IV antibiotics for several days.

Biomarker blood tests currently used in the NHS, such as CRP do not reliably differentiate beween severe bacterial infection (SBI) and inflammation, and show a delayed response to bacterial infection. PCT is a biomarker released in response to inflammatory stimuli including bacterial infections, with very high levels produced in SBI.[6] In contrast to CRP, PCT rises early and peaks early, and falls rapidly in response to effective antimicrobial therapy. This makes blood PCT potentially a better biomarker for monitoring progression of SBI and response to antimicrobial therapy, and for facilitating clinical decision making by informing initiation, change or discontinuation of antimicrobial therapy. It supports the aims of the Department of Health Five Year Antimicrobial Resistance Strategy 2019-2024 of conserving and stewarding the effectiveness of existing antimicrobials by ensuring that antibiotics are used responsibly and less often (i.e. antimicrobial stewardship (AMS)).[7]

There is strong evidence for introducing paediatric AMS programmes in hospital settings, in terms of reduced antibiotic use, improved quality of prescribing and cost-savings. Long-term and sustainable reductions in antimicrobial prescribing and a reduction of resistance rates at a population level have been achieved by the implementation of nationally coordinated, whole-system approaches, with no evidence of an increase in the rate of serious infection or bacterial complications.[8]

National Institute for Health and Care Excellence (NICE) guidance on AMS (https://www.nice.org.uk/guidance/ng15) recommends decision support systems as an AMS intervention. The use of a PCT-based algorithm to guide antibiotic stopping or escalation is one such decision support system which can be used. The AMS guidelines made research recommendations including RCTs to determine whether short or long

For peer review only - http://bmjopen.bmj.com/site/about/guidelines.xhtml

courses of antibiotics reduce AMR and whether using point-of-care tests is clinically and cost effective when prescribing antimicrobials in children with respiratory tract infections. The BATCH trial described in this paper is aligned with these recommendations in seeking to evaluate if PCT-guided management can result in shorter courses of antibiotics whilst not increasing the risk of adverse outcomes to paediatric patients.

A systematic review and cost-effectiveness analysis conducted on behalf of NICE (https://www.nice.org.uk/guidance/dg18) evaluated PCT testing to guide antibiotic therapy for the treatment of sepsis in intensive care settings and for suspected bacterial infection in emergency department (ED) settings in adults and children [1]. It concluded that addition of a PCT algorithm to the information used to guide antibiotic treatment may reduce antibiotic exposure in adults in intensive care unit (ICU) settings and in the ED without any adverse consequences and may also be associated with reductions in the length of hospital and ICU stay in adults. Evidence was not found on the effectiveness using a PCT algorithm to guide antibiotic treatment for children with suspected or confirmed SBI admitted from emergency care. None of the identified studies were conducted in the UK, and it was not clear whether the control arms of these studies were representative of standard practice in the UK. Therefore, the report recommended further studies to adequately assess the effectiveness of adding PCT algorithms to the information used to guide antibiotic treatment in adults and children with suspected or confirmed SBI in ICU settings and in adults and children with suspected bacterial infection in ED settings. It states that further studies are needed particularly for children, where data are currently lacking, and research examining (short-term) health-state utility values in the UK for adults and children with confirmed or suspected SBI in the ICU and ED.

A recent systematic review and meta-analysis of antibiotic duration for bacterial infections in children demonstrated that IV to oral switch can occur earlier than previously recommended.[9] The authors produced recommendations for antibiotic duration and IV to oral switch to support clinical decision making, and recommend prospective research on optimal antibiotic durations. The lack of good evidence on recommended duration of antibiotic therapy leads to an overuse of antibiotics, contributing to the development of AMR. Combating AMR has been identified as a national and global priority. Shorter courses

For peer review only - http://bmjopen.bmj.com/site/about/guidelines.xhtml

of antibiotic therapy would be associated with reductions in adverse effects for patients, and reductions in healthcare resource utilisation. Results from this research will inform recommendations relating to the duration of antibiotic use in future guideline updates including NICE sepsis guidelines.

Aims and Objectives

The BATCH trial aims to improve AMS in hospitalised children with suspected or confirmed bacterial infection, by reducing antibiotic duration with guidance from an additional PCT laboratory test.

Primary Objective

To determine if the addition of PCT testing to current best practice based on the NICE AMS guidelines can safely allow a reduction in duration of intravenous antibiotic therapy in hospitalised children with suspected or confirmed bacterial infection compared to current best practice alone.

To meet this objective specifically, we will assess;

- Duration of IV antibiotics
- Unscheduled admissions to PICU with infective diagnosis
- Readmissions to PICU with infective diagnosis
- Unscheduled readmissions with infective diagnosis within a week of stopping IV antibiotics
- Re-commencing IV antibiotics for the same infection within a week of stopping IV antibiotics
- Mortality

Secondary Objectives

To assess the effect of additional PCT testing to AMS best practice on:

- total duration of oral and IV antibiotics,
- time to switch from broad spectrum to narrow spectrum antibiotics,
- time to discharge from hospital, hospital acquired infection,
- suspected adverse drug reactions,
- cost of hospital episode, health related quality of life,

For peer review only - http://bmjopen.bmj.com/site/about/guidelines.xhtml

Also to provide detailed understanding of parent and health professionals attitudes to and experiences of, participating in the BATCH RCT.

METHODS AND ANALYSIS

Design

This is a multi-centre, prospective, two-arm, individually randomised controlled trial. An internal pilot will assess the site and participant absolute recruitment and consent rates, the proportion of participants undergoing PCT assessments and the ability to collect the primary outcome data.

Participants

Children admitted to hospital with suspected or confirmed SBI and commenced on IV antibiotics, in whom IV antibiotics are likely to be continued for more than 48 hours. Recruited from paediatric wards or Paediatric Intensive Care Units (PICUs) within children's hospitals and general hospitals in the UK. The eligibility criteria are described in Table 1.

Intervention

In children randomised to the intervention arm, a blood sample will be sent to the hospital laboratory for a PCT test at baseline/randomisation and every 1-3 days whilst still on IV antibiotics. This includes instances where IV antibiotics are restarted for the same infection (up to day 28 post-randomisation).

PCT tests will be performed on a bioMérieux VIDAS® platform. This is a semiautomated immunoassay system based on Enzyme Linked Fluorescent Assay principles. It requires 200µl of plasma or serum and can be run on a sample sent for routine biochemistry after the routine tests have been performed.

PCT results feed into an algorithm where thresholds have been defined by previous data (Figure 1). The algorithm provides both definitive guidelines, e.g. stop antibiotics if PCT

For peer review only - http://bmjopen.bmj.com/site/about/guidelines.xhtml

<0.25 ng/mL, and advisory guidelines, e.g. consider oral switch if PCT decreased by ≥80%. Clinicans can overrule the algothim if they feel it is appropriate to do so.

Children in the control arm will not have the PCT test performed.

Primary Outcome measure

The trial will use a co-primary outcome of antibiotic use and safety.

1) Antibiotic usage is defined as the number of days IV antibiotics used.
2) Safety is defined as the absence of all of the following:
    - Unscheduled admissions/re-admissions (to include re-admission within 7 days of discharge with infective diagnosis, unscheduled re-admission to PICU with infective diagnosis, or admission to PICU with infective diagnosis),
    - Re-treatment for same condition within 7 days of stopping IV antibiotics (re-starting IV antibiotics which have been stopped),
    - Death.

Secondary Outcome measures

- Total duration of antibiotics (IV and oral).
- Time to switch from broad spectrum to narrow spectrum antibiotics.
- Time to discharge from hospital.
- Suspected Adverse Drug Reactions (ADR) (categorised using the Liverpool Causality Assessment Tool).
- Cost of hospital episode.
- Hospital Acquired Infection (HAI) up to Day 28.
- Health utility as measured by the Child Health Utility questionnaire (CHU9D) up to Day 28.

For peer review only

For peer review only - http://bmjopen.bmj.com/site/about/guidelines.xhtml

Trial procedures

Data Collection

All data collection will be by electronic data capture using a bespoke database developed by the Centre for Trials Research (CTR) and hosted by Cardiff University secure servers. It is encrypted and accessed by individual username and password. Paper copies of all case report forms will be available.

Data Management

Details of data management procedures (such as checking for missing, illegible or unusual values (range checks)) will be specified in the BATCH Data Management Plan. Details of Monitoring procedures will be specified in the BATCH Monitoring Plan.

Identification and screening

Identification of potential participants will be by the clinical care team, or the clinical members of the research team involved in care of children on the ward, or the general paediatric or infectious diseases teams involved in care of children on the ward.

Informed consent

Informed consent will be obtained by those suitably trained and on the delegation log. Parent/carer(s) of children (or the child if over the age of 16 or Gillick competent) will be given a participant information sheet about the trial and will have time to consider before being asked to sign the consent form. The consent form includes storing samples for future research. Age appropriate information sheets will also be provided for children who are old enough to use them, and those deemed to have capacity will be asked to sign an age appropriate assent form, additional to their parent/carer's consent.

Once consented, participants will be allocated a unique identification number (participant ID). Separate informed consent will be taken for participation in the qualitative data collection.

For peer review only - http://bmjopen.bmj.com/site/about/guidelines.xhtml

Withdrawal

Participants may withdraw consent to participate in any aspect of the trial, at any time. Declining to participate or withdrawing from the trial will not affect the care of the child.

Randomisation and enrolment

Participants will be allocated to the trial arms in a 1:1 ratio using minimisation[10] via a secure (24-hour) web-based randomisation programme controlled centrally by the CTR. The covariates whose imbalance is to be minimised are age group and site. A random element will be added to the minimisation algorithm to reduce the risk of predictability and subversion. Full details are provided in the BATCH randomisation strategy.

All participants will be enrolled in the trial from the date of randomisation until Day 28 follow up. Participants will be assessed until they are discharged from clinical care. Figure 2 shows the trial schema.

During Hospitalisation

Outcome data described in Table 3 will be recorded daily by the research nurse for all recruited participants (up to and including Day 28, or until discharge). Observation and medication charts and medical notes will be reviewed. Assessments include antibiotic use, routine test results, PCT measurements and clinician adherence to the algorithm.

28 day follow up

At Day 28 (+2 week time window) parents will be contacted by telephone or email to ask about their child's healthcare resource use (e.g. hospital admissions, other prescribed medicines, over-the-counter medicines, GP attendance), direct non-medical costs (i.e. travel costs, child care costs, expenses incurred while in hospital, self-reported lost earnings) and the child's health related quality of life (i.e. CHU9D). If efforts to contact them by phone or email are unsuccessful, a questionnaire booklet will be posted out with a pre-paid envelope for return.

For peer review only - http://bmjopen.bmj.com/site/about/guidelines.xhtml

Public and patient involvement

Patient and Public Involvement (PPI) will be sought throughout the research process, from conceptualisation to dissemination. An example of this is the active involvement of the Liverpool GenerationR Young People's Advisory Group (YPAG) in contributing to the design of this research.[11] The group consists of 24 young people aged between 8 to 21 year olds who have worked with several researchers exploring the topic of developing tests to rapidly detect or diagnose SBI in children. YPAG members are well aware of the problems associated with diagnosing and treating sepsis and have discussed at length the issues associated with AMR and the need to educate young people and families about the misuse of antibiotics.

We will involve the Liverpool GenerationR YPAG, throughout the duration of the trial. The group will advise on children's information sheets and the production of educational materials for young people and families on the most appropriate use of antibiotics. We will invite parents and young people to contribute actively to dissemination events, including presenting parents' and young peoples' views and stories. Parent representatives will also be invited to attend Trial Management and Steering Group Meetings. Members of the YPAG and parents will be trained by our PPI Lead.

Safety Reporting

Events described in table 2 (i.e. hospitalisation and death) are primary outcomes of the trial and are recorded as part of primary outcome data collection, and therefore are not subject to expedited reporting. Serious adverse events will be reported if the event results in persistent or significant disability or incapacity or consists of a congenital anomaly or birth defect. An assessment of causality between the event and the trial intervention will be carried out by the Principal Investigator or delegated clinician, and then independently by a clinical reviewer who will also assess expectedness. If the clinical reviewer classifies the event as probably or definitely caused by the intervention, it will be classified as a serious adverse reaction.

For peer review only - http://bmjopen.bmj.com/site/about/guidelines.xhtml

Sample Size

Two co-primary outcomes (IV antibiotics duration and a composite safety outcome) are defined in this trial[12] and the overall sample size is determined by both.

The focus for the intervention is on moving the step down from IV to oral therapy earlier, and therefore the time until this step down is the primary outcome on antibiotic usage, and the trial is powered to detect if PCT-directed care is *superior* to standard care on time until switch from IV antibiotics. The size of potential shortening of time to detect an effect has been taken from a systematic review.[1] The safety co-primary (table 2) is a composite measure reflecting various outcomes which represent deterioration or lack of clinical response in the child, and would be expected to increase if IV antibiotics were being withdrawn inappropriately early.

In terms of IV antibiotic duration, a one day reduction[13] in antibiotics from an estimated median of 5 days in the control arm (from our observation data[14]) demonstrates a hazard ratio of 1.25. At a 5% significance level with 90% power, 844 participants with observed IV antibiotics duration are needed. In terms of the event rates of safety elements, an observational study a showed a re-admission rate of 15% at Day 28.[15] In critically ill patients, up to 3% reinstating IV antibiotic therapy rate, and 3% mortality were reported.[1 13 16] With some overlaps considered, we estimate around 15% overall rate of our composite safety outcome. The SAPS trial in adults used a non-inferiority margin of 8% for mortality.[1] Given the lower expected rate of safety outcomes in this population we have chosen a similar relative non-inferiority bound of 5%. This means increases in the composite safety measure of less than 5% (from 15 to 20%) using PCT guided therapy would be considered as not inferior. With a one-sided significance level of 0.05 and 90% power we would need 1748 participants to test non-inferiority. Overall, with 1748 effectively recruited participants, we would have 99% power to detect antibiotic duration decrease and 90% power to test non-inferiority in safety separately. Assuming that these two co-primary outcomes are independent, this would give us at least 89% power for the combined analysis.[17] By considering 10% loss to follow-up for the primary outcomes, our final targeted sample size is inflated to 1942 as achievable.

For peer review only - http://bmjopen.bmj.com/site/about/guidelines.xhtml

STATISTICAL ANALYSES

Primary Outcome

Our primary analysis of co-primary outcomes will be intention to treat and will compare between trial arms: a) the duration of days of IV antibiotics following randomisation using Cox regression; and b) the rate of adverse events using logistic regression, with a one-sided 95% confidence interval constructed to assess non-inferiority. This analysis will control for balancing factors in the randomisation. A positive conclusion will only be made if both a decrease in IV antibiotic duration AND non-inferiority in safety can be demonstrated (table 4). are observed. We will assess ifheterogeneity among centres exists and fit a two-level model with random centre effects if confirmed. Per-protocol and Complier average causal effect (CACE) analysis will also be undertaken to test the treatment effects to patients with fully utilised PCT algorithm (Table 5).

Secondary Outcomes

For secondary outcomes, differences in the proportion of ADR, unscheduled readmission, re-commencing IV antibiotics and 28-day mortality will be assessed separately by logistic regression models. We will also compare the total duration of antibiotics (IV and oral) and time to discharge from hospital between treatment groups via Kaplan Meier plots and Cox regression. Average utility will be compared between the two groups at 28 days using linear regression (Table 6). The ineligible/inevaluable participants will be excluded in secondary analyses (per protocol, CACE, etc.).

Sub group analysis

Analysis will be split by the organ system of the infection (i.e. lower urinary tract, lower respiratory, intra-abdominal, bacteraemia, skin and soft tissue, etc).

Missing, unused and spurious data

Missing primary outcome data are likely to be minimal, so complete case analysis will be used. However, if this exceeds more than 20% of participants we will employ multiple

For peer review only - http://bmjopen.bmj.com/site/about/guidelines.xhtml

imputation and report the impact on the treatment effect alongside the complete case analysis.

Economic evaluation

Health economic analysis will include direct and indirect costs associated with unscheduled admissions (to ward or PICU), re-admissions, re-starting IV antibiotics, and hospital-acquired infections. Descriptive and regression analysis will be used to identify key elements of service use and cost and to explore the potential impact of baseline participant characteristics on the costs and outcomes measures. Average cost per participant will be estimated at the end of the treatment and the follow-up periods, respectively, and average cost per sub-group of patients may be explored for the same time points. Bootstrapping and missing data imputation will be done if justified. Differences in each arm will be assessed and used for the computation of an incremental cost-effectiveness ratio (ICER). We will calculate ICERs for a clinically effective outcome (fewer days on IV antibiotics with increased or equivalent safety) and the cost per IV antibiotic day avoided.

A cost-effectiveness analysis will assess possible efficiency gains. A NHS perspective will be used and relevant direct medical costs will be collected. Information on resource use will include data on inpatient bed days, antibiotic consumption, nursing and medical resources, other medicines including over the counter medicines, diagnostic and monitoring laboratory tests, GP visits and emergency visits. Direct hospital costs will be calculated by multiplying resource use with the accompanying unit costs collected from patient level data in the participating hospitals, routine NHS sources (e.g. NHS reference costs and *British National Formulary* (BNFc) and from the manufacturer of the PCT test, as appropriate. Time horizon will be 28 days, therefore there is no need to consider a discount rate. Patients' health utility will be measured using CHU9D up to day 28.

Descriptive and regression analysis will be used to identify key elements of service use and cost and to explore the potential impact of baseline participant characteristics on the costs and outcomes measures. Differences in each arm will be assessed and used for the computation of anICER. One way sensitivity analysis will be carried out in key model

For peer review only - http://bmjopen.bmj.com/site/about/guidelines.xhtml

parameters. Probabilistic sensitivity analysis (PSA) and cost-effectiveness acceptability curves (CEACs) will be constructed. Information on direct non-medical costs, such as travelling to and from the hospitals, and indirect costs, such as parents' productivity losses, will also be collected.

Qualitative study

The qualitative evaluation will aim to explore the experiences and understanding of parents of children recruited into the BATCH trial (n= 10-15) about their child's condition and treatment of confirmed or suspected bacterial infection, and also to explore their views and experiences about participating in a RCT.

It will also aim to explore the views and experiences of health professionals involved in the BATCH trial (n=10-20) about participating in a RCT with a focus on acceptability of the trial, clinical equipoise, taking informed consent, and support needs of trial involvement. Interviews will be conducted at two time points (before or at the beginning and after the intervention) which will enable us to capture whether there are any changes in attitudes towards the PCT test. Semi-stuctured interviews will be undertaken face-to-face or via telephone. They will be recorded and transcribed. Data will be double coded and analysed using thematic analysis. Non-participant observation of episodes of patient care and trial delivery (of both arms) will also be carried out in some centres. Observations and field notes will enable an understanding of how the individual intervention components and delivery processes work in the real healthcare setting.

TRIAL MANAGEMENT

The trial is sponsored by University of Liverpool, coordinated by CTR, Cardiff University and hosted by Alder Hey Children's NHS Foundation Trust. The other partner organisations are University of Southampton, University Hospital Southampton NHS Foundation Trust, University Hospitals Bristol NHS Foundation Trust, Oxford University Hospitals NHS Foundation Trust, Sheffield Children's NHS Foundation Trust, Lancaster University and Hull University.

For peer review only - http://bmjopen.bmj.com/site/about/guidelines.xhtml

Trial Management Group

The Trial Management Group (TMG) will meet monthly throughout the course of the trial and will include the chief investigator co-applicants, collaborators, trial manager, data manager, statistician and administrator. A parent representative will also attend and contribute to the design and management of the trial, as well as patient-facing materials. TMG members will be required to sign up to the remit and conditions as set out in the TMG Charter.

Trial Steering Committee

A Trial Steering Committee (TSC) consisting of an independent chairperson, two independent members and a parent representative will provide oversight of the BATCH trial. Members will be required to sign up to the remit and conditions as set out in the TSC charters and will meet at least annually.

Independent Data Monitoring Committee

An Independent Data Monitoring Committee (IDMC) will provide oversight of all matters related to patient safety and data quality. Members will be required to sign up to the remit and conditions as set out in the IDMC charter and will meet at least annually.

Ethics and Dissemination

Research Approvals

The trial was approved by the Health Research Authority and NHS Research Ethics Committee (North West Liverpool East REC reference 18/NW/0100) on 13th April 2018. The following substantial amendments were made to the trial and were communicated to all trial sites: Amendment 2 (30 May 2018); Amendment 3 (13 July 2018); Amendment 5 (12 December 2018); Amendment 9 (9 August 2019); Amendment 14 (9 October 2020). The current protocol is version 5.0 dated 8 July 2020.

For peer review only - http://bmjopen.bmj.com/site/about/guidelines.xhtml

Dissemination Plan

Following completion of the trial, a final report will be prepared for the National Institute of Health Research (NIHR) HTA Journal series. Findings will be published in peer-reviewed journals and presented at international conferences. Nationally, we will engage with NICE, the Royal College of Paediatrics and Child Health, The British Society for Antimicrobial Chemotherapy, British Infection Society, and the British Paediatric Allergy, Immunity and Infection Group. All publications and presentations related to the trial will be authorised by the TMG in accordance with the BATCH publication policy. Where appropriate, the results of this trial can be directly implemented in the revisions of the NICE guidelines.

DISCUSSION

This trial has the potential to impact the clinical care of hospitalised children with confirmed or suspected SBI, which currently accounts for a large proportion of antibiotic use in hospitalised children. It will contribute to the development of PCT-guided antibiotic management guidelines of infections in hospitalised children, and will address the safety of shorter antibiotic courses. If shorter duration of PCT-guided antimicrobial therapy is shown to be safe and effective, this will have major implications for direct and indirect costs of childhood hospitalisations from infection. This will potentially lead to significant reductions in duration of hospitalisation and reduced antibiotic exposure, resulting in a positive impact on healthcare services and societal costs. Reduced exposure to antibiotics will, in turn, reduce AMR. This trial will demonstrate if introducing PCT-guided AMS management in the NHS is cost-effective. Reduced antibiotic use will lead to reduced hospital costs and reduced ADRs.

This trial is timely as it aligns with the current Department of Health Five Year action plan for antimicrobial resistance 2019 to 2024 and is a response to research recommendations from two published NICE guidance documents (DG18 and NG15).

Acknowledgements: In addition to the authors, the BATCH team comprises Helen Nabwera, Katrina Cathie, Daniel Owens, Jenny Whitbread, Zoe Gray, Zoe Oliver, Judith Evans, Sarah Jones, Sam Clarkstone, Abin Thomas, Kim Smallman and Sarah Milosevic. The

For peer review only - http://bmjopen.bmj.com/site/about/guidelines.xhtml

authors would like to thank the research nurses, clinicians and laboratory staff for their support during the trial. They would also like to acknowledge the contribution of the Liverpool GenerationR YPAG, the TSC members, namely Ann Marie Swart, Paul Brocklehurst, Colette Smith, Paul Dark, Phil Shackley, and Kelly Chapman, and IDMC members, namely Graeme MacLennan, Danielle Horton-Taylor, Philip Howard and Alastair Sutcliffe. The CTR receives funding from Health and Care Research Wales and Cancer Research UK.

Author Contributions: EC is the chief investigator of this trial. EC along with JB, SNF, SP, LH, SVP, ET-J, LB-H, CM, CH, CP, JP, MP and KH led the development of the research question, study design, obtaining the funding and implementation of the protocol. CAW is the trial manager and ET-J is the senior trial manager who coordinate the operational delivery of the trial protocol and recruitment. LB-H is the lead qualitative researcher. CH and PP are the trial statisticians. DH is the data manager. All authors listed provided critical review and final approval of the manuscript.

Funding: This trial was funded by the National Institute of Health Research Health Technology Assessment (NIHR HTA) Programme, reference 15/188/42. Sponsored by University of Liverpool, Research Support Office, 2nd Floor Block D, Waterhouse Building, 3 Brownlow Street, Liverpool, L69 3GL. Contact person: Mr Alex Astor; Sponsor@Liverpool.ac.uk, Sponsor reference: UoL001333. The study is supported by the NIHR Clinical Research Network, NIHR Southampton Clinical Research Facility,

Disclaimer: The views expressed are those of the authors and not necessarily those of the NHS, the NIHR or the Department of Health and Social Care. Neither the Sponsor nor the Funder had any role on the study design; collection, management, analysis and interpretation of data; writing of this manuscript or in the decision to submit this manuscript for publication.

Competing interests: EDC is a member of NIHR Invention for Innovation panel, member of NICE Diagnostic advisory committee April 2014-September 2020, Member of MRC DPFS panel and MRC COVID-19 Agile panel. SNF is an NIHR Senior Investigator. All other authors declare no competing interests.

For peer review only - http://bmjopen.bmj.com/site/about/guidelines.xhtml

Patient and public involvement: Patients and/or the public were involved in the design, or conduct, or reporting, or dissemination plans of this research. Refer to the Methods section for further details.

Patient consent for publication: Not required.

Provenance and peer review: Not commissioned; externally peer reviewed.

Word count: 4176

For peer review only

For peer review only - http://bmjopen.bmj.com/site/about/guidelines.xhtml

References

1. Westwood M, Ramaekers B, Whiting P, et al. Procalcitonin testing to guide antibiotic therapy for the treatment of sepsis in intensive care settings and for suspected bacterial infeaction in emergency department settings: a systematic review and ocst-effectiveness analysis. *Health Technology Assessment* 2015;19(96) doi: 10.3310/hta19960
2. Singer M, Deutschman C, Seymour C, et al. The Third International Consensus Definitions for Sepsis and Septic Shock (Sepsis-3). *JAMA* 2016;315(8):801-10. doi: 10.1001/jama.2016.0287
3. Kumar A, Roberts D, Wood K, et al. Duration of hypotension before initiation of effective antimicrobial therapy is the critical determinant of survival in human septic shock. *Critical Care Medicine* 2006;34(6):1589-96.
4. Fischer J, Ramser M, Fanconi S. Use of antibiotics in pediatric intensive care and potential savings. *Intensive Care Medicine* 2000;27(7):959-66.
5. Goldmann D, Weinstein R, Wenzel R, et al. Strategies to Prevent and Control the Emergence and Spread of Antimicrobial-Resistant Microrganisms in Hospitals. A challenge to hospital leadership. *JAMA* 1996;275(3):234-40.
6. Carrol E, Thomson A, Hart C. Procalcitonin as a marker of sepsis. *International Journal of Microbial Agents* 2002;20(1):1-9.
7. Department of Health. Tackling Antimicrobial Resistence 2019-2024. The UK's five-year national action plan, 2019.
8. Patel S, Vergnano S. The impact of paediatric antimicrobial stewardship programmes on patient outcomes. *Curr Opin Infect Dis* 2018;31(3):216-23. doi: doi:10.1097/QCO.0000000000000449
9. McMullan B, Andersen D, Blyth C, et al. Antibiotic duration and timing of the swtich from intravenous to oral route for bacterial infections in children: systematic review and guidelines. *Lancet Infectious Diseases* 2016;16(8):e139-52. doi: 10.1016/S1473-3099(16)30024-X
10. Altman D, Bland J. Treatment allocation by minimisation. *BMJ* 2005;330:843.
11. Generation R Young People Improving Research [Available from: https://generationr.org.uk/liverpool
12. Gillespie D, Francis N, Carrol E, et al. Use of co-primary outcomes for trials of antimicrobial stewardship interventions. *The Lancet Infectious Diseases* 2018;18(6):595-97.
13. Paediatric Intensive Care Audit Network (PICANet). Annual Report (published November 2015), 2015.
14. Irwin A, Grant A, Williams R, et al. Predicting Risk of Serious Bacterial Infections in Febrile Children in the Emergency Department. *Pediatrics* 2017;140(2):e20162853.
15. Bustinduy ACS, I. Shaw, R. Irwin, A. Thiagarajan, J. Beynon, R. Ladhani, S. Sharland, M. CABIN network. Predictors of fever-related admissions to a paediatric assessment unit, ward and reattendances in a South London emergency department: the CABIN 2 study. . *Arch Dis Child* 2017;102(1):22-28. doi: doi:10.1136/archdischild-2016-310494
16. D'Souza SG, R. Jennings, R. Siner S. Paulus, S. Thorburn, K. Chesters, C. Downey, C. Baines, P. Lane, S. Carrol, ED. Procalcitonin and Other Common Biomarkers Do Not Reliably Identify Patients at Risk for Bacterial Infection After Congenital Heart

For peer review only - http://bmjopen.bmj.com/site/about/guidelines.xhtml

Surgery. . *Pediatr Crit Care Med* 2019;20(3):243-51. doi: doi:10.1097/PCC.0000000000001826
17. Chuang-Stein C, Stryszak P, Dmitrienko A, et al. Challenge of multiple co-primary endpoints: a new approach. *Stat Med* 2007;26(6):1181-92. doi: 10.1002/sim.2604
18. de Jong E, van Oers J, Beischuizen A, et al. Efficacy and safety of procalitonin guidance in reducing the duration of antibiotic treatment in critically ill patients: a randomised, controlled, open-label trial. *Lancet Infectious Diseases* 2016 doi: 10.1016/S1473-3099(16)00053-0

For peer review only

For peer review only - http://bmjopen.bmj.com/site/about/guidelines.xhtml

Table 1 Eligibility Criteria

Inclusion criteria	Exclusion criteria
• All children aged between 72 hours old and up to 18 years old admitted to hospital for confirmed or suspected severe bacterial infection (SBI), in whom intravenous (IV) antibiotics are commenced, and who are expected to remain on IV antibiotics for more than 48 hours.<br><br>Conditions include (but not limited to):<br>bacteraemia, central line-associated bloodstream infections (CLABSIs), uncomplicated bone and joint infections (such as single site infection, osteomyelitis with adjacent septic arthritis or septic arthritis with adjacent osteomyelitis), discitis, empyema, pneumonia, pyelonephritis, sinusitis, retropharyngeal abscess, pyomyositis, uncomplicated culture-negative meningitis, intra-abdominal infections, lymphadenitis, cellulitis.<br><br>• First time in the BATCH trial.	• Preterm infants age <37 weeks corrected gestational age, under 72 hours old or ≥18 years of age.<br>• Children admitted moribund and not expected to survive more than 24 hours.<br>• Children with a predicted duration of IV antibiotics of less than 48 hours.<br>• Children not expected to survive at least 28 days because of a pre-existing condition.<br>• Children with bacterial meningitis, bacterial endocarditis, or brain abscess.<br>• Children with complicated bone and joint infections.<br>• Children receiving antibiotics for surgical prophylaxis.<br>• Children with chronic co-morbidities, such as cystic fibrosis, chronic lung disease, bronchiectasis where there is already a pre-defined length of course of antibiotics.<br>• Children who are severely immunocompromised (e.g. chemotherapy, stem cell transplant, biological therapy for inflammatory or rheumatological conditions).<br>• Children who, in the opinion of the local investigator, are unsuitable for randomisation due to high probability of requiring sustained IV therapy.<br>• Children with a presence of existing directive to withhold life-sustaining treatment.<br>• Inborn infants admitted to Neonatal Intensive Care Units (NICU), Neonatal High Dependency Units (NHDU), Special Care Baby Units (SCBU) or Postnatal wards.

For peer review only

Table 2 Elements of the Composite Safety Outcome

Composite element	Definition	Reason for inclusion	Expected prevalence in usual care	Potential direction of change with intervention
Unscheduled admissions/ Readmissions	Admitted/readmitted to PICU or unplanned readmission to hospital within 7 days of stopping IV antibiotics	Indicators of a deterioration and need for increased level of care	Our observation study showed 8.8% patients have admissions/re-admissions[14]	Increase
Reinstating IV antibiotic therapy	Restarting IV antibiotic (for any reason) therapy within 7 days of stopping IV therapy	Indicator of potentially inappropriate withdrawal of IV antibiotics and deterioration	de Jong study 2.9% in control arm re-started IV antibiotic[18]	Increase
Mortality	Death for any reason in the 28 days following randomisation	-	PICANet Annual report 2015: deaths on PICUs ~4% in 2012-2014[13]	Increase

For peer review only - http://bmjopen.bmj.com/site/about/guidelines.xhtml

Table 3 Schedule of enrolment, interventions and assessments

Data Type		Source Data	Data type	Screening	Baseline	Post randomisation until discharged home	Follow up (Day 28)	Frequency	By whom
1	Informed Consent	Consent form	-	X				Once	Site Clinical/Research team
2	Eligibility Assessment	Eligibility Case Report Form (CRF)	-	X				Once	Site research team
3	Demographics	CRF			X			Once	Research Nurse
4	Admission data	CRF	Comorbidities, preadmission antibiotic use, initial working diagnosis		X			Once	Research Nurse
5	Health-related Quality of Life	QRF	CHU9D		X		X	Twice	Patient/parent reported (over telephone or post at Day 28)
6	Randomisation	CRF	-		X			Once	Site Research team
7	Antibiotic use (IV/Oral)	Observation charts/ Medical notes	Antibiotic type, dose, duration, including			X		Daily	Research Nurse
8	Blood tests including PCT	CRF/Medical notes	Routine blood tests<br>PCT results (for those in intervention arm)			X			
9	Clinical review	CRF/Medical notes	Clinical decision made and whether the algorithm was complied with			X		As required when a clinical decision has been made	

For peer review only - http://bmjopen.bmj.com/site/about/guidelines.xhtml

Data Type		Source Data	Data type	Screening	Baseline	Post randomisation until discharged home	Follow up (Day 28)	Frequency	By whom
10	Cerebrospinal fluid metrics, radiology and microbiology	CRF/Medical notes	White cell count, biochemistry. Microbiology results, radiology results			X			
11	Re-commencing of Antibiotics (IV and oral	Observation charts/ Medical notes	Antibiotic type, dose, duration, time recommenced			X		Daily	Research Nurse
12	Unscheduled Admissions	Medical notes	PICU readmissions post discharge			X		Daily	Research Nurse
13	Mortality	Medical notes	Date, description			X		If before Day 28	Research Nurse
14	Discharge	Medical notes	Date, description			X		If before Day 28	Research Nurse
15	Adverse events	Observation charts/ Medical notes	Date, type			X		Daily	Research Nurse
16	Suspected Adverse Drug Reactions (ADR)	Liverpool Causality Assessment tool	Date, description			X		Daily	Research Nurse
17	Resource Use	Questionnaire	Direct Medical costs (Inc. medication and ventilation and vasopressor) and Resource Use				X	Once	Patient/parent reported (over telephone or post)
18	SAE	SAE form			←------------------------As Required----------------------→				Research Nurse
19	Withdrawals	Withdrawal form			←------------------------As Required----------------------→				Research Nurse, Centre for Trial Research

For peer review only - http://bmjopen.bmj.com/site/about/guidelines.xhtml

Table 4 Combined Primary Outcome

	Antibiotic duration different (reduction in PCT group) (H1)	Antibiotic duration no different (no reduction in PCT group) H0)
Safety composite not worse in PCT group (H1)	✔	✖
Safety composite worse in PCT group (H0)	✖	✖

✔ - Intervention successful; ✖ - Intervention unsuccessful

Table 5 Summary of analyses of co-primary outcomes

	Co-primary outcomes	Analysis approach	Covariates in the model
Primary analysis	Duration of days of IV antibiotics (Intervention effect)	Cox regression (superiority test)	Trial arm and minimisation factors (site, age group)
	Adverse events (composite safety outcome)	Logistic regression (non-inferiority test)	Trial arm and minimisation factors (site, age group)
Secondary analyses	Duration of days of IV antibiotics (Intervention effect)	Kaplan Meier plot	Trial arm
		Log rank test	Trial arm
		Cox regression (assessments of suspected baseline confounders)	Covariates in the primary analysis, plus suspected baseline confounders (e.g. gender)
		Complier Average Causal Effect (CACE)	Covariates in the primary analysis, plus intervention adherence
	Adverse events (composite safety outcome)	Logistic regression (assessments of suspected baseline confounders)	Covariates in the primary analysis, plus suspected baseline confounders (e.g. gender)

Table 6 Summary of analyses of secondary outcomes

Secondary outcomes	Analysis approach	Covariates in the model
Proportion of ADR	Logistic regression	Trial arm and minimisation factors (site, age group)
Proportion of unscheduled readmission		
Proportion of re-commencing IV antibiotics		
Proportion of mortality		
Duration of antibiotics (IV and oral)	Cox regression	Trial arm and minimisation factors (site, age group)
Time to discharge from hospital		
Average utility	Linear regression	Trial arm and minimisation factors (site, age group)

For peer review only - http://bmjopen.bmj.com/site/about/guidelines.xhtml

Figure 1 Guidelines for continuing or stopping IV antibiotics

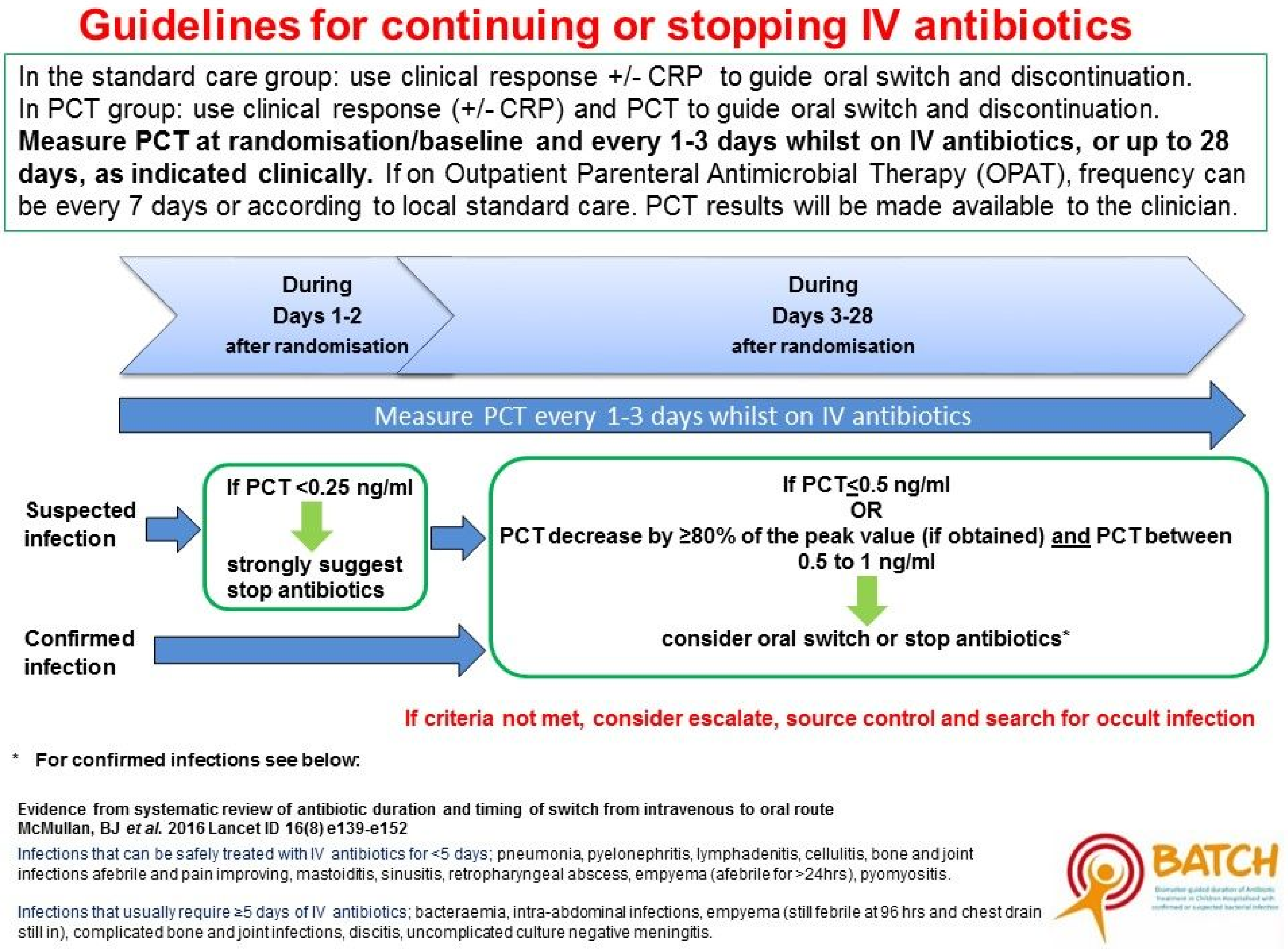

Figure 2 Trial Schema / Participant flow

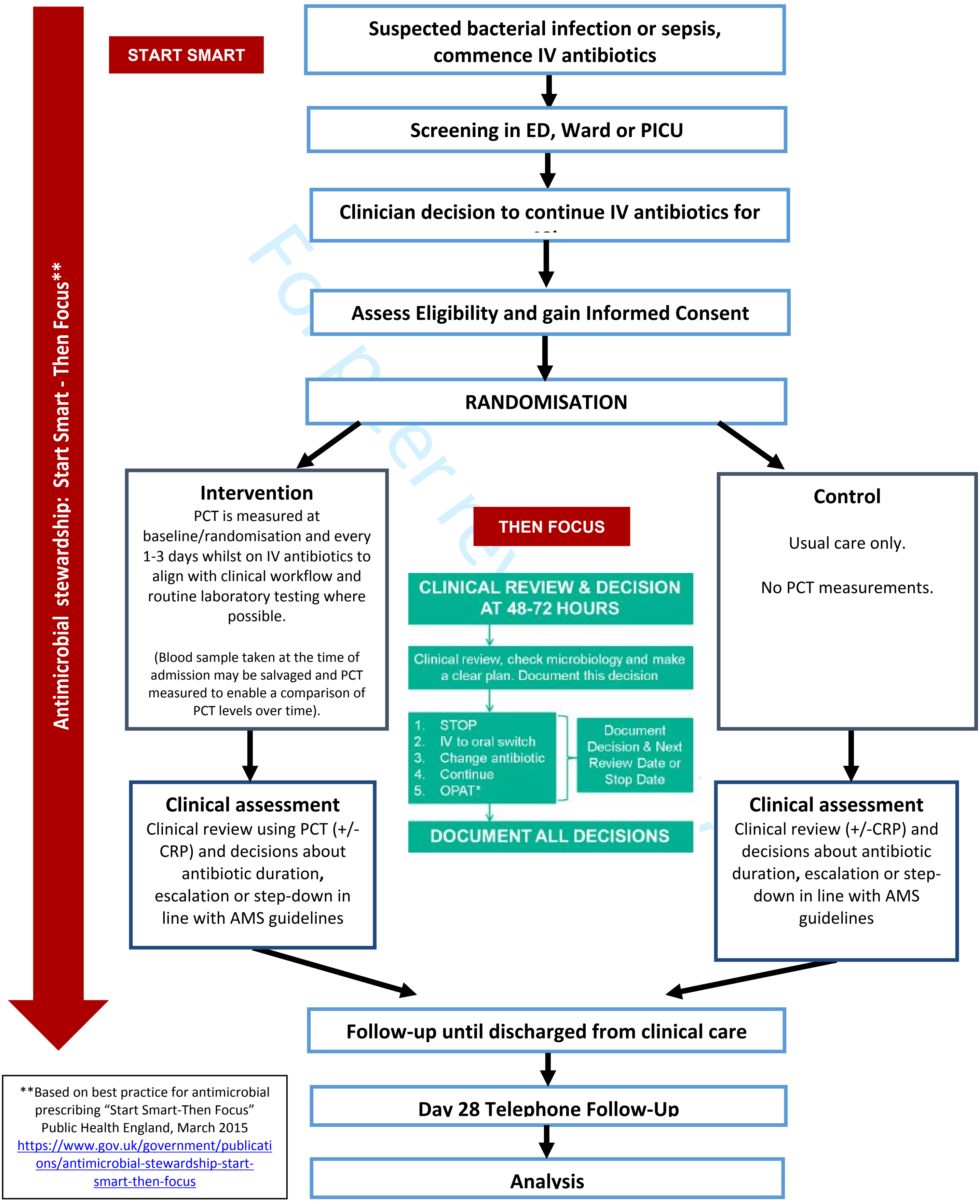

For peer review only

For peer review only - http://bmjopen.bmj.com/site/about/guidelines.xhtml

Reporting checklist for protocol of a clinical trial.

Based on the SPIRIT guidelines.

Instructions to authors

Complete this checklist by entering the page numbers from your manuscript where readers will find each of the items listed below.

Your article may not currently address all the items on the checklist. Please modify your text to include the missing information. If you are certain that an item does not apply, please write "n/a" and provide a short explanation.

Upload your completed checklist as an extra file when you submit to a journal.

In your methods section, say that you used the SPIRITreporting guidelines, and cite them as:

Chan A-W, Tetzlaff JM, Gøtzsche PC, Altman DG, Mann H, Berlin J, Dickersin K, Hróbjartsson A, Schulz KF, Parulekar WR, Krleža-Jerić K, Laupacis A, Moher D. SPIRIT 2013 Explanation and Elaboration: Guidance for protocols of clinical trials. BMJ. 2013;346:e7586

		Reporting Item	Page Number
**Administrative information**			
Title	#1	Descriptive title identifying the study design, population, interventions, and, if applicable, trial acronym	1
Trial registration	#2a	Trial identifier and registry name. If not yet registered, name of intended registry	1
Trial registration: data set	#2b	All items from the World Health Organization Trial Registration Data Set	1
Protocol version	#3	Date and version identifier	15
Funding	#4	Sources and types of financial, material, and other support	17
Roles and responsibilities: contributorship	#5a	Names, affiliations, and roles of protocol contributors	1 and 17

For peer review only

Roles and responsibilities: sponsor contact information	#5b	Name and contact information for the trial sponsor	17
Roles and responsibilities: sponsor and funder	#5c	Role of study sponsor and funders, if any, in study design; collection, management, analysis, and interpretation of data; writing of the report; and the decision to submit the report for publication, including whether they will have ultimate authority over any of these activities	17
Roles and responsibilities: committees	#5d	Composition, roles, and responsibilities of the coordinating centre, steering committee, endpoint adjudication committee, data management team, and other individuals or groups overseeing the trial, if applicable (see Item 21a for data monitoring committee)	15
**Introduction**			
Background and rationale	#6a	Description of research question and justification for undertaking the trial, including summary of relevant studies (published and unpublished) examining benefits and harms for each intervention	2-5
Background and rationale: choice of comparators	#6b	Explanation for choice of comparators	6-7
Objectives	#7	Specific objectives or hypotheses	5
Trial design	#8	Description of trial design including type of trial (eg, parallel group, crossover, factorial, single group), allocation ratio, and framework (eg, superiority, equivalence, non-inferiority, exploratory)	6
**Methods: Participants, interventions, and outcomes**			
Study setting	#9	Description of study settings (eg, community clinic, academic hospital) and list of countries where data will be collected. Reference to where list of study sites can be obtained	8

For peer review only

Eligibility criteria	#10	Inclusion and exclusion criteria for participants. If applicable, eligibility criteria for study centres and individuals who will perform the interventions (eg, surgeons, psychotherapists)	6 and Table 1
Interventions: description	#11a	Interventions for each group with sufficient detail to allow replication, including how and when they will be administered	7
Interventions: modifications	#11b	Criteria for discontinuing or modifying allocated interventions for a given trial participant (eg, drug dose change in response to harms, participant request, or improving / worsening disease)	n/a
Interventions: adherance	#11c	Strategies to improve adherence to intervention protocols, and any procedures for monitoring adherence (eg, drug tablet return; laboratory tests)	n/a
Interventions: concomitant care	#11d	Relevant concomitant care and interventions that are permitted or prohibited during the trial	n/a
Outcomes	#12	Primary, secondary, and other outcomes, including the specific measurement variable (eg, systolic blood pressure), analysis metric (eg, change from baseline, final value, time to event), method of aggregation (eg, median, proportion), and time point for each outcome. Explanation of the clinical relevance of chosen efficacy and harm outcomes is strongly recommended	7
Participant timeline	#13	Time schedule of enrolment, interventions (including any run-ins and washouts), assessments, and visits for participants. A schematic diagram is highly recommended (see Figure)	9 and Figure 2
Sample size	#14	Estimated number of participants needed to achieve study objectives and how it was determined, including clinical and statistical assumptions supporting any sample size calculations	11
Recruitment	#15	Strategies for achieving adequate participant enrolment to reach target sample size	8
**Methods: Assignment of interventions (for controlled trials)**			
Allocation: sequence generation	#16a	Method of generating the allocation sequence (eg, computer-generated random numbers), and list of any factors for stratification. To reduce predictability of a random sequence, details of any planned restriction (eg, blocking) should be	9

For peer review only

		provided in a separate document that is unavailable to those who enrol participants or assign interventions	
Allocation concealment mechanism	#16b	Mechanism of implementing the allocation sequence (eg, central telephone; sequentially numbered, opaque, sealed envelopes), describing any steps to conceal the sequence until interventions are assigned	9
Allocation: implementation	#16c	Who will generate the allocation sequence, who will enrol participants, and who will assign participants to interventions	9
Blinding (masking)	#17a	Who will be blinded after assignment to interventions (eg, trial participants, care providers, outcome assessors, data analysts), and how	n/a
Blinding (masking): emergency unblinding	#17b	If blinded, circumstances under which unblinding is permissible, and procedure for revealing a participant's allocated intervention during the trial	n/a
**Methods: Data collection, management, and analysis**			
Data collection plan	#18a	Plans for assessment and collection of outcome, baseline, and other trial data, including any related processes to promote data quality (eg, duplicate measurements, training of assessors) and a description of study instruments (eg, questionnaires, laboratory tests) along with their reliability and validity, if known. Reference to where data collection forms can be found, if not in the protocol	8-9 and Table 2
Data collection plan: retention	#18b	Plans to promote participant retention and complete follow-up, including list of any outcome data to be collected for participants who discontinue or deviate from intervention protocols	9
Data management	#19	Plans for data entry, coding, security, and storage, including any related processes to promote data quality (eg, double data entry; range checks for data values). Reference to where details of data management procedures can be found, if not in the protocol	8
Statistics: outcomes	#20a	Statistical methods for analysing primary and secondary outcomes. Reference to where other details of the statistical analysis plan can be found, if not in the protocol	12-14

For peer review only

Statistics: additional analyses	#20b	Methods for any additional analyses (eg, subgroup and adjusted analyses)	12
Statistics: analysis population and missing data	#20c	Definition of analysis population relating to protocol non-adherence (eg, as randomised analysis), and any statistical methods to handle missing data (eg, multiple imputation)	12-13
**Methods: Monitoring**			
Data monitoring: formal committee	#21a	Composition of data monitoring committee (DMC); summary of its role and reporting structure; statement of whether it is independent from the sponsor and competing interests; and reference to where further details about its charter can be found, if not in the protocol. Alternatively, an explanation of why a DMC is not needed	15
Data monitoring: interim analysis	#21b	Description of any interim analyses and stopping guidelines, including who will have access to these interim results and make the final decision to terminate the trial	n/a
Harms	#22	Plans for collecting, assessing, reporting, and managing solicited and spontaneously reported adverse events and other unintended effects of trial interventions or trial conduct	10
Auditing	#23	Frequency and procedures for auditing trial conduct, if any, and whether the process will be independent from investigators and the sponsor	8
**Ethics and dissemination**			
Research ethics approval	#24	Plans for seeking research ethics committee / institutional review board (REC / IRB) approval	15
Protocol amendments	#25	Plans for communicating important protocol modifications (eg, changes to eligibility criteria, outcomes, analyses) to relevant parties (eg, investigators, REC / IRBs, trial participants, trial registries, journals, regulators)	15
Consent or assent	#26a	Who will obtain informed consent or assent from potential trial participants or authorised surrogates, and how (see Item 32)	8

For peer review only

Consent or assent: ancillary studies	#26b	Additional consent provisions for collection and use of participant data and biological specimens in ancillary studies, if applicable	8
Confidentiality	#27	How personal information about potential and enrolled participants will be collected, shared, and maintained in order to protect confidentiality before, during, and after the trial	8
Declaration of interests	#28	Financial and other competing interests for principal investigators for the overall trial and each study site	17
Data access	#29	Statement of who will have access to the final trial dataset, and disclosure of contractual agreements that limit such access for investigators	n/a
Ancillary and post trial care	#30	Provisions, if any, for ancillary and post-trial care, and for compensation to those who suffer harm from trial participation	n/a
Dissemination policy: trial results	#31a	Plans for investigators and sponsor to communicate trial results to participants, healthcare professionals, the public, and other relevant groups (eg, via publication, reporting in results databases, or other data sharing arrangements), including any publication restrictions	16
Dissemination policy: authorship	#31b	Authorship eligibility guidelines and any intended use of professional writers	n/a
Dissemination policy: reproducible research	#31c	Plans, if any, for granting public access to the full protocol, participant-level dataset, and statistical code	n/a
**Appendices**			
Informed consent materials	#32	Model consent form and other related documentation given to participants and authorised surrogates	n/a
Biological specimens	#33	Plans for collection, laboratory evaluation, and storage of biological specimens for genetic or molecular analysis in the current trial and for future use in ancillary studies, if applicable	8

Notes:

- 10: 6 and Table 1
- 13: 8-9 and Figure 2

For peer review only

- 18a: 8-9 and Table 2 The SPIRIT checklist is distributed under the terms of the Creative Commons Attribution License CC-BY-ND 3.0. This checklist was completed on 29. November 2020 using https://www.goodreports.org/, a tool made by the EQUATOR Network in collaboration with Penelope.ai

For peer review only

BMJ Open

Biomarker-guided duration of Antibiotic Treatment in Children Hospitalised with confirmed or suspected bacterial infection (BATCH): Protocol for a randomised controlled trial.

Journal:	*BMJ Open*
Manuscript ID	bmjopen-2020-047490.R1
Article Type:	Protocol
Date Submitted by the Author:	20-Jul-2021
Complete List of Authors:	Waldron, Cherry-Ann; Cardiff University, Centre for Trials Research<br>Thomas-Jones, Emma; Cardiff University, Centre for Trials Research<br>Bernatoniene, Jolanta; Bristol Royal Hospital for Children, Paediatric Infectious Disease Department<br>Brookes-Howell, Lucy; South East Wales Trials Unit,<br>Faust, Saul; University of Southampton, UK, NIHR Wellcome Trust Clincial Research Facility<br>Harris, Debbie; Cardiff University, Centre for Trials Research<br>Hinds, Lucy ; Sheffield Children's NHS Foundation Trust, Paediatrics<br>Hood, Kerenza; Cardiff University,<br>Huang, Chao; Hull York Medical School,<br>Mateus, Ceu; University of Lancaster, Division of Health Research<br>Pallmann, Philip; Cardiff University, College of Biomedical and Life Sciences, Centre for Trials Research<br>Patel, Sanjay; Southampton Children's Hospital<br>Paulus, Stephane; University of Oxford, Paediatrics; University of Liverpool Institute of Infection and Global Health,<br>Peak, Matthew; Alder Hey Children's NHS Foundation Trust, R&D<br>Powell, Colin; Children's Hospital for Wales, Paediatrics<br>Preston, Jenny<br>Carrol, Enitan; University of Liverpool, Institute of Infection, Veterinary and Ecological Sciences
**Primary Subject Heading**:	Paediatrics
Secondary Subject Heading:	Infectious diseases
Keywords:	INFECTIOUS DISEASES, Paediatric intensive & critical care < INTENSIVE & CRITICAL CARE, Diagnostic microbiology < INFECTIOUS DISEASES, Public health < INFECTIOUS DISEASES

SCHOLARONE™
Manuscripts

Biomarker-guided duration of **A**ntibiotic **T**reatment in **C**hildren **H**ospitalised with confirmed or suspected bacterial infection (BATCH): Protocol for a randomised controlled trial.

Waldron CA[1*], Thomas-Jones E[1], Bernatoniene J[2], Brookes-Howell L[1], Faust S.N[3], Harris D[1], Hinds L[4], Hood K[1], Huang C[5], Mateus C[6], Pallmann P[1], Patel S[7], Paulus S[8], Peak M[9], Powell C[10], Preston J[9], Carrol ED[11]

[*]Corresponding author
Dr Cherry-Ann Waldron, Centre for Trials Research, Cardiff University, 7th Floor, Neuadd Meirionnydd, Heath Park, Cardiff, CF14 4YS, Tel: +44(0)29 20687609, Email: waldronc@cardiff.ac.uk

1 Centre for Trials Research, Cardiff University, Cardiff, UK
2 University Hospitals Bristol NHS Foundation Trust
3 University Hospital Southampton NHS Foundation Trust, Southampton, UK
4 Sheffield Children's NHS Foundation Trust, Sheffield, UK
5 Hull York Medical School, Hull, University of Hull, UK
6 Division of Health Research, Lancaster University, Lancaster, UK
7 Faculty of Medicine, University of Southampton, University Hospital Southampton NHS Foundation Trust, Southampton
8 Oxford University Hospitals NHS Foundation Trust, Oxford, UK
9 Alder Hey Children's NHS Foundation Trust, Liverpool, UK
10 Child Health, School of Medicine, Cardiff University, UK / Sidra Medicine, Doha, Qatar
11 University of Liverpool, Liverpool, UK

Key words: Paediatrics, Bacterial infection, Procalcitonin, Antibiotic duration

ABSTRACT

Introduction

Procalcitonin (PCT) is a biomarker more specific for bacterial infection and responds quicker than other commonly used biomarkers such as C-Reactive Protein, but is not routinely used in the NHS. Studies mainly in adults show that using PCT to guide clinicians may reduce antibiotic use, reduce hospital stay, with no associated adverse effects such as increased rates of hospital re-admission, incomplete treatment of infections, relapse or death. A review conducted for NICE recommends further research on PCT testing to guide antibiotic use in children.

For peer review only

For peer review only - http://bmjopen.bmj.com/site/about/guidelines.xhtml

Methods and analysis

BATCH is a multi-centre, prospective, two-arm, individually RCT with a 28 day follow up and internal pilot. The intervention is a PCT-guided algorithm used in conjunction with best practice. The control arm is best practice alone. We plan to recruit 1942 children, aged between 72 hours and up to 18 years old, who are admitted to hospital and being treated with intravenous antibiotics for suspected or confirmed bacterial infection. Co-primary outcomes are duration of antibiotic use and a composite safety measure. Secondary outcomes include time to switch from broad to narrow spectrum antibiotics, time to discharge, adverse drug reactions, health utility and cost effectiveness. We will also perform a qualitative process evaluation.

Ethics and dissemination

The trial protocol was approved by the HRA and NHS REC (North West Liverpool East REC reference 18/NW/0100). We will publish the results in international peer-reviewed journals and present at scientific meetings.

Trial registration: ISRCTN 11369832

Strengths and limitations of this trial

- First trial to evaluate effectiveness of PCT to guide antibiotic duration in children hospitalised with suspected or confirmed bacterial infection.
- Potential to inform NICE guidelines and impact clinical care, by reducing exposure to antibiotics, leading to a reduction in antimicrobial resistance.
- The multicentre design including patients from hospital sites in England and Wales.
- Due to the type of intervention it is not possible to blind patients and clinicians.
- Potential shift in patient population following a recruitment break due to COVID-19 pandemic will be addressed with sensitivity analyses.

INTRODUCTION

Sepsis is defined as life-threatening organ dysfunction caused by a dysregulated host response to infection.[1] Sepsis causes many non-specific symptoms and signs that can also be

For peer review only - http://bmjopen.bmj.com/site/about/guidelines.xhtml

caused by a large number of conditions that may or may not be due to infection, and that may or may not require immediate or urgent treatment. Sepsis is usually caused by bacteria, although viral and fungal causes do occur. The problem for clinicians is the difficulty in distinguishing bacterial sepsis from other conditions presenting with similar non-specific signs and symptoms. Prompt administration of antibiotics reduces mortality by half,[2] but indiscriminate antibiotic use unnecessarily increases antimicrobial resistance (AMR), resulting in increased costs in hospitalised patients,[3 4] which represents a significant resource burden in the NHS. Not all children admitted with bacterial infection will meet the criteria for sepsis, but they could still have serious infection, requiring IV antibiotics for several days.

Biomarker blood tests currently used in the NHS, such as C-Reactive Protein (CRP) do not reliably differentiate beween severe bacterial infection (SBI) (defined previously [5]) and inflammation, and show a delayed response to bacterial infection. PCT is a biomarker released in response to inflammatory stimuli including bacterial infections, with very high levels produced in SBI.[6] In contrast to CRP, PCT rises early and peaks early, and falls rapidly in response to effective antimicrobial therapy. This makes blood PCT potentially a better biomarker for monitoring progression of SBI and response to antimicrobial therapy, and for facilitating clinical decision making by informing initiation, change or discontinuation of antimicrobial therapy. It supports the aims of the Department of Health Five Year Antimicrobial Resistance Strategy 2019-2024 of conserving and stewarding the effectiveness of existing antimicrobials by ensuring that antibiotics are used responsibly and less often (i.e. antimicrobial stewardship (AMS)).[7]

There is strong evidence for introducing paediatric AMS programmes in hospital settings, in terms of reduced antibiotic use, improved quality of prescribing and cost-savings. Long-term and sustainable reductions in antimicrobial prescribing and a reduction of resistance rates at a population level have been achieved by the implementation of nationally coordinated, whole-system approaches, with no evidence of an increase in the rate of serious infection or bacterial complications.[8] We recently published a large study prospectively assessing the performance of multiple biomarkers of serious bacterial infection (SBI) in a heterogeneous cohort of critically ill children and uniquely profiled longitudinal biomarker changes.

For peer review only - http://bmjopen.bmj.com/site/about/guidelines.xhtml

Longitudinal profiles for PCT showed the greatest percentage drop in values over the first seven days of therapy in children with SBI, suggesting that PCT might be useful in guiding duration of antimicrobial therapy in children [9].

National Institute for Health and Care Excellence (NICE) guidance on AMS (https://www.nice.org.uk/guidance/ng15) recommends decision support systems as an AMS intervention. The use of a PCT-based algorithm to guide antibiotic stopping or escalation is one such decision support system which can be used. The AMS guidelines made research recommendations including RCTs to determine whether short or long courses of antibiotics reduce AMR and whether using point-of-care tests is clinically and cost effective when prescribing antimicrobials in children with respiratory tract infections. The BATCH trial described in this paper is aligned with these recommendations in seeking to evaluate if PCT-guided management can result in shorter courses of antibiotics whilst not increasing the risk of adverse outcomes to paediatric patients.

A systematic review and cost-effectiveness analysis conducted on behalf of NICE (https://www.nice.org.uk/guidance/dg18) evaluated PCT testing to guide antibiotic therapy for the treatment of sepsis in intensive care settings and for suspected bacterial infection in emergency department (ED) settings in adults and children [10]. It concluded that addition of a PCT algorithm to the information used to guide antibiotic treatment may reduce antibiotic exposure in adults in intensive care unit (ICU) settings and in the ED without any adverse consequences and may also be associated with reductions in the length of hospital and ICU stay in adults. Evidence was not found on the effectiveness using a PCT algorithm to guide antibiotic treatment for children with suspected or confirmed SBI admitted from emergency care. None of the identified studies were conducted in the UK, and it was not clear whether the control arms of these studies were representative of standard practice in the UK. Therefore, the report recommended further studies to adequately assess the effectiveness of adding PCT algorithms to the information used to guide antibiotic treatment in adults and children with suspected or confirmed SBI in ICU settings and in adults and children with suspected bacterial infection in ED settings. It states that further studies are needed particularly for children, where data are currently lacking, and research examining (short-

For peer review only - http://bmjopen.bmj.com/site/about/guidelines.xhtml

term) health-state utility values in the UK for adults and children with confirmed or suspected SBI in the ICU and ED.

A recent systematic review and meta-analysis of antibiotic duration for bacterial infections in children demonstrated that IV to oral switch can occur earlier than previously recommended.[11] The authors produced recommendations for antibiotic duration and IV to oral switch to support clinical decision making, and recommend prospective research on optimal antibiotic durations. The lack of good evidence on recommended duration of antibiotic therapy leads to an overuse of antibiotics, contributing to the development of AMR. Combating AMR has been identified as a national and global priority. Shorter courses of antibiotic therapy would be associated with reductions in adverse effects for patients, and reductions in healthcare resource utilisation. Results from this research will inform recommendations relating to the duration of antibiotic use in future guideline updates including NICE sepsis guidelines.

Aims and Objectives

The BATCH trial aims to improve AMS in hospitalised children with suspected or confirmed bacterial infection, by reducing antibiotic duration with guidance from an additional PCT laboratory test.

Primary Objective

To determine if the addition of PCT testing to current best practice based on the NICE AMS guidelines can safely allow a reduction in duration of intravenous antibiotic therapy in hospitalised children with suspected or confirmed bacterial infection compared to current best practice alone.

To meet this objective specifically, we will assess;

- Duration of IV antibiotics
- Unscheduled admissions to PICU with infective diagnosis
- Readmissions to PICU with infective diagnosis
- Unscheduled readmissions with infective diagnosis within a week of stopping IV antibiotics

For peer review only - http://bmjopen.bmj.com/site/about/guidelines.xhtml

- Re-commencing IV antibiotics for the same infection within a week of stopping IV antibiotics
- Mortality

Secondary Objectives

To assess the effect of additional PCT testing to AMS best practice on:

- total duration of oral and IV antibiotics,
- time to switch from broad spectrum to narrow spectrum antibiotics,
- time to discharge from hospital,
- hospital acquired infection,
- suspected adverse drug reactions,
- cost of hospital episode,
- health related quality of life,

Also to provide detailed understanding of parent and health professionals attitudes to and experiences of, participating in the BATCH RCT.

METHODS AND ANALYSIS

Design

This is a multi-centre, prospective, two-arm, individually randomised controlled trial. An internal pilot will assess the site and participant absolute recruitment and consent rates, the proportion of participants undergoing PCT assessments and the ability to collect the primary outcome data.

Participants

Children admitted to hospital with suspected or confirmed SBI (diagnosed by the clinical team) and commenced on IV antibiotics, in whom IV antibiotics are likely to be continued for more than 48 hours. Recruited from paediatric wards or Paediatric Intensive Care Units (PICUs) within children's hospitals and general hospitals in the UK (approximately n=10). The eligibility criteria are described in Table 1.

For peer review only - http://bmjopen.bmj.com/site/about/guidelines.xhtml

Intervention

In children randomised to the intervention arm, a blood sample will be sent to the hospital laboratory for a PCT test at baseline/randomisation and every 1-3 days whilst still on IV antibiotics to align with clinical workflow and routine laboratory testing where possible. This includes instances where IV antibiotics are restarted for the same infection (up to day 28 post-randomisation). An additional 1ml (minimum 0.5ml) lithium heparin samples will be collected for PCT analysis.

PCT tests will be performed on a bioMérieux VIDAS® platform. It is a prerequisite that participating sites have access this platform to take part in the trial. This is a semiautomated immunoassay system based on Enzyme Linked Fluorescent Assay principles. Calibration is performed in line with manufacturer's guidelines. It is simple and flexible to use and gives results in 20 minutes. It requires 200µl of plasma or serum and can be run on a sample sent for routine biochemistry after the routine tests have been performed.

PCT results feed into an algorithm where thresholds have been defined by previous data (Figure 1). The algorithm provides both definitive guidelines, e.g. stop antibiotics if PCT <0.25 ng/mL, and advisory guidelines, e.g. consider oral switch if PCT decreased by ≥80%. Clinicians can overrule the algorithm if they feel it is appropriate to do so. The algorithm values are based on our now published work which is the largest study to prospectively assess the performance of multiple biomarkers of SBI in a heterogeneous cohort of critically ill children and uniquely profiles longitudinal biomarker changes within the cohort. [9]

Children in the control arm will not have the PCT test performed.

Primary Outcome measure

The trial will use a co-primary outcome of antibiotic use and safety.

1) Antibiotic usage is defined as the number of days IV antibiotics used.
2) Safety is defined as the absence of all of the following:

For peer review only - http://bmjopen.bmj.com/site/about/guidelines.xhtml

- Unscheduled admissions/re-admissions (to include re-admission within 7 days of discharge with infective diagnosis, unscheduled re-admission to PICU with infective diagnosis, or admission to PICU with infective diagnosis),
- Re-treatment for same condition within 7 days of stopping IV antibiotics (re-starting IV antibiotics which have been stopped),
- Death.

Secondary Outcome measures

- Total duration of antibiotics (IV and oral).
- Time to switch from broad spectrum to narrow spectrum antibiotics.
- Time to discharge from hospital.
- Suspected Adverse Drug Reactions (ADR) (categorised using the Liverpool Causality Assessment Tool comprising ten modified questions with their dichotomous responses into a flowchart to arrive at one of four outcome categories: 'definite', 'probable', 'possible', or 'unlikely'. It has high interrater agreement). [12,13]
-
- Cost of hospital episode.
- Hospital Acquired Infection (HAI) as defined by the clinical team up to Day 28.
- Health utility as measured by the Child Health Utility questionnaire (CHU9D) [14] up to Day 28.

Internal Pilot

An internal pilot phase will be conducted over the first eight months of the recruitment period with six lead sites. Predefined progression criteria will be used to assess feasibility to progress to the full trial, such as site and patient absolute recruitment and consent rate, proportion of patients undergoing PCT assessments and the ability to collect primary outcome data.

Trial procedures

Data Collection

For peer review only - http://bmjopen.bmj.com/site/about/guidelines.xhtml

All data collection will be by electronic data capture using a bespoke database developed by the Centre for Trials Research (CTR) and hosted by Cardiff University secure servers. It is encrypted and accessed by individual username and password. Paper copies of all case report forms will be available. Essential documents will be kept securely in a locked cupboard, and at the end of the trial, will be archived at an approved external storage facility for 10 years.

Data Management

Details of data management procedures (such as checking for missing, illegible or unusual values (range checks)) will be specified in the BATCH Data Management Plan. Details of Monitoring procedures will be specified in the BATCH Monitoring Plan.

Identification and screening

Identification of potential participants will be by the clinical care team, or the clinical members of the research team involved in care of children on the ward, or the general paediatric or infectious diseases teams involved in care of children on the ward. This includes a member of the research team visiting the wards where children with SBI are admitted to assess eligibility and screening admissions lists.

Informed consent

Informed consent will be obtained by those suitably trained and on the delegation log. Parent/carer(s) of children (or the child if over the age of 16 or Gillick competent) will be given a participant information sheet (Supplementary file 1) about the trial and will have time to consider before being asked to sign the consent form (Supplementary file 2). The consent form includes storing samples for future research. Age appropriate information sheets will also be provided for children who are old enough to use them, and those deemed to have capacity will be asked to sign an age appropriate assent form, additional to their parent/carer's consent.

For peer review only - http://bmjopen.bmj.com/site/about/guidelines.xhtml

Once consented, participants will be allocated a unique identification number (participant ID). Separate informed consent will be taken for participation in the qualitative data collection.

Withdrawal

Participants may withdraw consent to participate in any aspect of the trial, at any time. Declining to participate or withdrawing from the trial will not affect the care of the child.

Randomisation and enrolment

Children will only be randomised if the clinician expects IV antibiotics will be prescribed for longer than 48 hours. This will typically be between 20-48 hours after admission, to fit with clinical work flow of ward rounds and phlebotomy times for routine blood tests.

Participants will be allocated to the trial arms in a 1:1 ratio using minimisation[15] via a secure (24-hour) web-based randomisation programme controlled centrally by the CTR. The covariates whose imbalance is to be minimised are age group and site. A random element will be added to the minimisation algorithm (as described in [15]) to reduce the risk of predictability and subversion. Full details are provided in the BATCH randomisation strategy. To further minimise the risk of subversion, the specific age groups and the random element will not be disclosed to those involved in recruiting participants until the end of the recruitment period.

All participants will be enrolled in the trial from the date of randomisation until Day 28 follow up. Participants will be assessed until they are discharged from clinical care. Figure 2 shows the trial schema.

During Hospitalisation

Outcome data described in Table 2 will be recorded daily by the research nurse for all recruited participants (up to and including Day 28, or until discharge). Observation and

For peer review only - http://bmjopen.bmj.com/site/about/guidelines.xhtml

medication charts and medical notes will be reviewed. Assessments include antibiotic use, routine test results, PCT measurements and clinician adherence to the algorithm.

28 day follow up

At Day 28 (+2 week time window) parents will be contacted by telephone or email to ask about their child's healthcare resource use (e.g. hospital admissions, other prescribed medicines, over-the-counter medicines, GP attendance), direct non-medical costs (i.e. travel costs, child care costs, expenses incurred while in hospital, self-reported lost earnings) and the child's health related quality of life (i.e. CHU9D). If efforts to contact them by phone or email are unsuccessful, a questionnaire booklet will be posted out with a pre-paid envelope for return.

Public and patient involvement

Patient and Public Involvement (PPI) will be sought throughout the research process, from conceptualisation to dissemination. An example of this is the active involvement of the Liverpool GenerationR Young People's Advisory Group (YPAG) in contributing to the design of this research.[16] The group consists of 24 young people aged between 8 to 21 year olds who have worked with several researchers exploring the topic of developing tests to rapidly detect or diagnose SBI in children. YPAG members are well aware of the problems associated with diagnosing and treating sepsis and have discussed at length the issues associated with AMR and the need to educate young people and families about the misuse of antibiotics.

We will involve the Liverpool GenerationR YPAG, throughout the duration of the trial. The group will advise on children's information sheets and the production of educational materials for young people and families on the most appropriate use of antibiotics. We will invite parents and young people to contribute actively to dissemination events, including

For peer review only - http://bmjopen.bmj.com/site/about/guidelines.xhtml

presenting parents' and young peoples' views and stories. Parent representatives will also be invited to attend Trial Management and Steering Group Meetings. Members of the YPAG and parents will be trained by our PPI Lead.

Safety Reporting

Events described in table 3 (i.e. hospitalisation and death) are primary outcomes of the trial and are recorded as part of primary outcome data collection, and therefore are not subject to expedited reporting. Serious adverse events will be reported if the event results in persistent or significant disability or incapacity or consists of a congenital anomaly or birth defect. An assessment of causality between the event and the trial intervention will be carried out by the Principal Investigator or delegated clinician, and then independently by a clinical reviewer who will also assess expectedness. If the clinical reviewer classifies the event as probably or definitely caused by the intervention, it will be classified as a serious adverse reaction.

Sample Size

Two co-primary outcomes (IV antibiotics duration and a composite safety outcome) are defined in this trial[17] and the overall sample size is determined by both.

The focus for the intervention is on moving the step down from IV to oral therapy earlier, and therefore the time until this step down is the primary outcome on antibiotic usage, and the trial is powered to detect if PCT-directed care is *superior* to standard care on time until switch from IV antibiotics. The size of potential shortening of time to detect an effect has been taken from a systematic review.[10] The safety co-primary (table 3) is a composite measure reflecting various outcomes which represent deterioration or lack of clinical response in the child, and would be expected to increase if IV antibiotics were being withdrawn inappropriately early.

For peer review only - http://bmjopen.bmj.com/site/about/guidelines.xhtml

In terms of IV antibiotic duration, a one day reduction[18] in antibiotics from an estimated median of 5 days in the control arm (from our observation data[5]) demonstrates a hazard ratio of 1.25. At a 5% significance level with 90% power, 844 participants with observed IV antibiotics duration are needed. In terms of the event rates of safety elements, an observational study a showed a re-admission rate of 15% at Day 28.[19] In critically ill patients, up to 3% reinstating IV antibiotic therapy rate, and 3% mortality were reported.[10 18 20] With some overlaps considered, we estimate around 15% overall rate of our composite safety outcome. The SAPS trial in adults used a non-inferiority margin of 8% for mortality.[10] Given the lower expected rate of safety outcomes in this population we have chosen a similar relative non-inferiority bound of 5%. This means increases in the composite safety measure of less than 5% (from 15 to 20%) using PCT guided therapy would be considered as not inferior. With a one-sided significance level of 0.05 and 90% power we would need 1748 participants to test non-inferiority. Overall, with 1748 effectively recruited participants, we would have 99% power to detect antibiotic duration decrease and 90% power to test non-inferiority in safety separately. Assuming that these two co-primary outcomes are independent, this would give us at least 89% power for the combined analysis.[21] By considering 10% loss to follow-up for the primary outcomes, our final targeted sample size is inflated to 1942 as achievable.

STATISTICAL ANALYSES

Primary Outcome

Our primary analysis of co-primary outcomes will be intention to treat and will compare between trial arms: a) the duration of days of IV antibiotics following randomisation using Cox regression; and b) the rate of adverse events using logistic regression, with a one-sided 95% confidence interval constructed to assess non-inferiority. This analysis will control for balancing factors in the randomisation. A positive conclusion will only be made if both a decrease in IV antibiotic duration AND non-inferiority in safety can be demonstrated (Table 4). We will assess if heterogeneity among centres exists and fit a two-level model with random centre effects if confirmed. Per-protocol and complier average causal effect (CACE) analysis will also be undertaken to test the treatment effects to patients with fully utilised

For peer review only - http://bmjopen.bmj.com/site/about/guidelines.xhtml

PCT algorithm (Table 5). No interim analysis will be performed. The Trial Statistician will be blinded to the study group allocation.

Secondary Outcomes

For secondary outcomes, differences in the proportion of ADR, HAI, unscheduled readmission, re-commencing IV antibiotics and 28-day mortality will be assessed separately by logistic regression models. We will report the median (interquartile range [IQR]) of the total duration of antibiotics (IV and oral), time to switch from broad to narrow spectrum antibiotics and time to discharge from hospital in both treatment groups and assess the group differences via Kaplan Meier plots and Cox regression. Average utility will be compared between the two groups at 28 days using linear regression (Table 6). The ineligible/inevaluable participants will be excluded in secondary analyses (per protocol, CACE, etc.).

Sub group analysis

Analysis will be split by the organ system of the infection (i.e. lower urinary tract, lower respiratory, intra-abdominal, bacteraemia, skin and soft tissue, etc).

Missing, unused and spurious data

Missing primary outcome data are likely to be minimal, so complete case analysis will be used. However, if this exceeds more than 20% of participants we will employ multiple imputation and report the impact on the treatment effect alongside the complete case analysis.

Economic evaluation

For peer review only - http://bmjopen.bmj.com/site/about/guidelines.xhtml

Health economic analysis will include direct and indirect costs associated with unscheduled admissions (to ward or PICU), re-admissions, re-starting IV antibiotics, and hospital-acquired infections. Descriptive and regression analysis will be used to identify key elements of service use and cost and to explore the potential impact of baseline participant characteristics on the costs and outcomes measures. Average cost per participant will be estimated at the end of the treatment and the follow-up periods, respectively, and average cost per sub-group of patients may be explored for the same time points. Bootstrapping and missing data imputation will be done if justified. Differences in each arm will be assessed and used for the computation of an incremental cost-effectiveness ratio (ICER). We will calculate ICERs for a clinically effective outcome (fewer days on IV antibiotics with increased or equivalent safety) and the cost per IV antibiotic day avoided.

A cost-effectiveness analysis will assess possible efficiency gains. A NHS perspective will be used and relevant direct medical costs will be collected. Information on resource use will include data on inpatient bed days, antibiotic consumption, nursing and medical resources, other medicines including over the counter medicines, diagnostic and monitoring laboratory tests, GP visits and emergency visits. Direct hospital costs will be calculated by multiplying resource use with the accompanying unit costs collected from patient level data in the participating hospitals, routine NHS sources (e.g. NHS reference costs and *British National Formulary* (BNFc) and from the manufacturer of the PCT test, as appropriate. Time horizon will be 28 days, therefore there is no need to consider a discount rate. Patients' health utility will be measured using CHU9D up to day 28.

Descriptive and regression analysis will be used to identify key elements of service use and cost and to explore the potential impact of baseline participant characteristics on the costs and outcomes measures. Differences in each arm will be assessed and used for the computation of anICER. One way sensitivity analysis will be carried out in key model parameters. Probabilistic sensitivity analysis (PSA) and cost-effectiveness acceptability curves (CEACs) will be constructed. Information on direct non-medical costs, such as travelling to and from the hospitals, and indirect costs, such as parents' productivity losses, will also be collected.

For peer review only - http://bmjopen.bmj.com/site/about/guidelines.xhtml

Qualitative study

The qualitative evaluation will aim to explore the experiences and understanding of parents of children recruited into the BATCH trial (n= 10-15) about their child's condition and treatment of confirmed or suspected bacterial infection, and also to explore their views and experiences about participating in a RCT. Interviews will be conducted after the participant reaches their day 28 follow up. A purposive sample strategy will be employed to include parents from both intervention and control arms and inclusion of different site.

It will also aim to explore the views and experiences of health professionals involved in the BATCH trial (n=10-20) about participating in a RCT with a focus on acceptability of the trial, clinical equipoise, taking informed consent, and support needs of trial involvement. Interviews will be conducted at two time points (before or at the beginning and after the intervention) which will enable us to capture whether there are any changes in attitudes towards the PCT test. A purposive sample strategy will be developed to address representation from up to 5 different sites and variation in health professional role (e.g. ward nurse. consultant, research nurse etc.)

With regards to the sample size for both health professionals and parents, the qualitative researcher(s) will make pragmatic decisions along with the research team regarding when enough is known about certain themes (I.e. data saturation has occurred.

Semi-structured interviews will be undertaken face-to-face or via telephone. They will be recorded and transcribed. Data will be double coded and analysed using thematic analysis. Non-participant observation of episodes of patient care and trial delivery (of both arms) will also be carried out in some centres. Observations and field notes will enable an understanding of how the individual intervention components and delivery processes work in the real healthcare setting.

TRIAL MANAGEMENT

The trial is sponsored by University of Liverpool, coordinated by CTR, Cardiff University and hosted by Alder Hey Children's NHS Foundation Trust. The other partner organisations are

For peer review only - http://bmjopen.bmj.com/site/about/guidelines.xhtml

University of Southampton, University Hospital Southampton NHS Foundation Trust, University Hospitals Bristol NHS Foundation Trust, Oxford University Hospitals NHS Foundation Trust, Sheffield Children's NHS Foundation Trust, Lancaster University and Hull University.

Trial Management Group

The Trial Management Group (TMG) will meet monthly throughout the course of the trial and will include the chief investigator co-applicants, collaborators, trial manager, data manager, statistician and administrator. A parent representative will also attend and contribute to the design and management of the trial, as well as patient-facing materials. TMG members will be required to sign up to the remit and conditions as set out in the TMG Charter.

Trial Steering Committee

A Trial Steering Committee (TSC) consisting of an independent chairperson, two independent members and a parent representative will provide oversight of the BATCH trial. Members will be required to sign up to the remit and conditions as set out in the TSC charters and will meet at least annually.

Independent Data Monitoring Committee

An Independent Data Monitoring Committee (IDMC) will provide oversight of all matters related to patient safety and data quality. Members will be required to sign up to the remit and conditions as set out in the IDMC charter and will meet at least annually.

Ethics and Dissemination

Research Approvals

The trial was approved by the Health Research Authority and NHS Research Ethics Committee (North West Liverpool East REC reference 18/NW/0100) on 13th April 2018. The following substantial amendments were made to the trial and were communicated to all

For peer review only - http://bmjopen.bmj.com/site/about/guidelines.xhtml

trial sites: Amendment 2 (30 May 2018); Amendment 3 (13 July 2018); Amendment 5 (12 December 2018); Amendment 9 (9 August 2019); Amendment 14 (9 October 2020). The current protocol is version 5.0 dated 8 July 2020.

Dissemination Plan

Following completion of the trial, a final report will be prepared for the National Institute of Health Research (NIHR) HTA Journal series. Findings will be published in peer-reviewed journals and presented at international conferences. Nationally, we will engage with NICE, the Royal College of Paediatrics and Child Health, The British Society for Antimicrobial Chemotherapy, British Infection Society, and the British Paediatric Allergy, Immunity and Infection Group. All publications and presentations related to the trial will be authorised by the TMG in accordance with the BATCH publication policy. Where appropriate, the results of this trial can be directly implemented in the revisions of the NICE guidelines.

DISCUSSION

This trial has the potential to impact the clinical care of hospitalised children with confirmed or suspected SBI, which currently accounts for a large proportion of antibiotic use in hospitalised children. It will contribute to the development of PCT-guided antibiotic management guidelines of infections in hospitalised children, and will address the safety of shorter antibiotic courses. If shorter duration of PCT-guided antimicrobial therapy is shown to be safe and effective, this will have major implications for direct and indirect costs of childhood hospitalisations from infection. This will potentially lead to significant reductions in duration of hospitalisation and reduced antibiotic exposure, resulting in a positive impact on healthcare services and societal costs. Reduced exposure to antibiotics will, in turn, reduce AMR. This trial will demonstrate if introducing PCT-guided AMS management in the NHS is cost-effective. Reduced antibiotic use will lead to reduced hospital costs and reduced ADRs.

For peer review only - http://bmjopen.bmj.com/site/about/guidelines.xhtml

This trial is timely as it aligns with the current Department of Health Five Year action plan for antimicrobial resistance 2019 to 2024 and is a response to research recommendations from two published NICE guidance documents (DG18 and NG15).

Acknowledgements: In addition to the authors, the BATCH team comprises Helen Nabwera, Katrina Cathie, Daniel Owens, Jenny Whitbread, Zoe Gray, Zoe Oliver, Judith Evans, Sarah Jones, Sam Clarkstone, Abin Thomas, Kim Smallman and Sarah Milosevic. The authors would like to thank the research nurses, clinicians and laboratory staff for their support during the trial. They would also like to acknowledge the contribution of the Liverpool GenerationR YPAG, the TSC members, namely Ann Marie Swart, Paul Brocklehurst, Colette Smith, Paul Dark, Phil Shackley, and Kelly Chapman, and IDMC members, namely Graeme MacLennan, Danielle Horton-Taylor, Philip Howard and Alastair Sutcliffe. The CTR receives funding from Health and Care Research Wales and Cancer Research UK.

Author Contributions: EDC is the chief investigator of this trial. EDC along with JB, SNF, SP, LH, SVP, ET-J, LB-H, CM, CH, CP, JP, MP and KH led the development of the research question, study design, obtaining the funding and implementation of the protocol. CAW is the trial manager and ET-J is the senior trial manager who coordinate the operational delivery of the trial protocol and recruitment. LB-H is the lead qualitative researcher. CH and PP are the trial statisticians. DH is the data manager. All authors listed provided critical review and final approval of the manuscript.

Funding: This trial was funded by the National Institute of Health Research Health Technology Assessment (NIHR HTA) Programme, reference 15/188/42. Sponsored by University of Liverpool, Research Support Office, 2nd Floor Block D, Waterhouse Building, 3 Brownlow Street, Liverpool, L69 3GL. Contact person: Mr Neil French; Sponsor@Liverpool.ac.uk, Sponsor reference: UoL001333. The study is supported by the NIHR Clinical Research Network, NIHR Southampton Clinical Research Facility,

Disclaimer: The views expressed are those of the authors and not necessarily those of the NHS, the NIHR or the Department of Health and Social Care. Neither the Sponsor nor the Funder had any role on the study design; collection, management, analysis and

For peer review only - http://bmjopen.bmj.com/site/about/guidelines.xhtml

interpretation of data; writing of this manuscript or in the decision to submit this manuscript for publication.

Competing interests: EDC is a member of NIHR Invention for Innovation panel, member of NICE Diagnostic advisory committee April 2014-September 2020, Member of MRC DPFS panel and MRC COVID-19 Agile panel. SNF is an NIHR Senior Investigator. All other authors declare no competing interests.

Patient and public involvement: Patients and/or the public were involved in the design, or conduct, or reporting, or dissemination plans of this research. Refer to the Methods section for further details.

Patient consent for publication: Not required.

Provenance and peer review: Not commissioned; externally peer reviewed.

Word count: 5161

For peer review only

For peer review only - http://bmjopen.bmj.com/site/about/guidelines.xhtml

References

1. Singer M, Deutschman C, Seymour C, et al. The Third International Consensus Definitions for Sepsis and Septic Shock (Sepsis-3). *JAMA* 2016;315(8):801-10. doi: 10.1001/jama.2016.0287
2. Kumar A, Roberts D, Wood K, et al. Duration of hypotension before initiation of effective antimicrobial therapy is the critical determinant of survival in human septic shock. *Critical Care Medicine* 2006;34(6):1589-96.
3. Fischer J, Ramser M, Fanconi S. Use of antibiotics in pediatric intensive care and potential savings. *Intensive Care Medicine* 2000;27(7):959-66.
4. Goldmann D, Weinstein R, Wenzel R, et al. Strategies to Prevent and Control the Emergence and Spread of Antimicrobial-Resistant Microrganisms in Hospitals. A challenge to hospital leadership. *JAMA* 1996;275(3):234-40.
5. Irwin A, Grant A, Williams R, et al. Predicting Risk of Serious Bacterial Infections in Febrile Children in the Emergency Department. *Pediatrics* 2017;140(2):e20162853.
6. Carrol E, Thomson A, Hart C. Procalcitonin as a marker of sepsis. *International Journal of Microbial Agents* 2002;20(1):1-9.
7. Department of Health. Tackling Antimicrobial Resistence 2019-2024. The UK's five-year national action plan, 2019.
8. Patel S, Vergnano S. The impact of paediatric antimicrobial stewardship programmes on patient outcomes. *Curr Opin Infect Dis* 2018;31(3):216-23. doi: doi:10.1097/QCO.0000000000000449
9. Nielsen M, Baines P, Jennings R, et al. Procalcitonin, C-reactive protein, neutrophil gelatinase-associated lipocalin, resistin and the APTT waveform for the early diagnosis of serious bacterial infection and prediction of outcome in critically ill children. *PLoS One* 2021;16(2)
10. Westwood M, Ramaekers B, Whiting P, et al. Procalcitonin testing to guide antibiotic therapy for the treatment of sepsis in intensive care settings and for suspected bacterial infeaction in emergency department settings: a systematic review and ocst-effectiveness analysis. *Health Technology Assessment* 2015;19(96) doi: 10.3310/hta19960
11. McMullan B, Andersen D, Blyth C, et al. Antibiotic duration and timing of the swtich from intravenous to oral route for bacterial infections in children: systematic review and guidelines. *Lancet Infectious Diseases* 2016;16(8):e139-52. doi: 10.1016/S1473-3099(16)30024-X
12. Gallagher R, Kirkham J, Mason J, et al. Development and inter-rater reliability of the Liverpool adverse drug reaction causality assessment tool. . *PLoS One* 2011;6(12):e28096.
13. Bracken L, Nunn A, Kirkham J, et al. Development of the Liverpool Adverse Drug Reaction Avoidability Assessment Tool. *PLoS ONE* 2017;12(1):e0169393. doi: doi:10.1371/journal.pone.0169393
14. Stevens K. Valuation of the Child Health Utility 9D Index. *PharmacoEcononics* 2012;30:729–47.
15. Altman D, Bland J. Treatment allocation by minimisation. *BMJ* 2005;330:843.
16. Generation R Young People Improving Research [Available from: https://generationr.org.uk/liverpool

For peer review only - http://bmjopen.bmj.com/site/about/guidelines.xhtml

17. Gillespie D, Francis N, Carrol E, et al. Use of co-primary outcomes for trials of antimicrobial stewardship interventions. *The Lancet Infectious Diseases* 2018;18(6):595-97.
18. Paediatric Intensive Care Audit Network (PICANet). Annual Report (published November 2015), 2015.
19. Bustinduy ACS, I. Shaw, R. Irwin, A. Thiagarajan, J. Beynon, R. Ladhani, S. Sharland, M. CABIN network. Predictors of fever-related admissions to a paediatric assessment unit, ward and reattendances in a South London emergency department: the CABIN 2 study. . *Arch Dis Child* 2017;102(1):22-28. doi: doi:10.1136/archdischild-2016-310494
20. D'Souza SG, R. Jennings, R. Siner S. Paulus, S. Thorburn, K. Chesters, C. Downey, C. Baines, P. Lane, S. Carrol, ED. Procalcitonin and Other Common Biomarkers Do Not Reliably Identify Patients at Risk for Bacterial Infection After Congenital Heart Surgery. . *Pediatr Crit Care Med* 2019;20(3):243-51. doi: doi:10.1097/PCC.0000000000001826
21. Chuang-Stein C, Stryszak P, Dmitrienko A, et al. Challenge of multiple co-primary endpoints: a new approach. *Stat Med* 2007;26(6):1181-92. doi: 10.1002/sim.2604
22. NICE. Meningitis (bacterial) and meningococcal septicaemia in under 16s: recognition, diagnosis and management. Clinical guideline [CG102]. 2010
23. de Jong E, van Oers J, Beischuizen A, et al. Efficacy and safety of procalitonin guidance in reducing the duration of antibiotic treatment in critically ill patients: a randomised, controlled, open-label trial. *Lancet Infectious Diseases* 2016 doi: 10.1016/S1473-3099(16)00053-0

For peer review only

For peer review only - http://bmjopen.bmj.com/site/about/guidelines.xhtml

Table 1 Eligibility Criteria

Inclusion criteria	Exclusion criteria
• All children aged between 72 hours old and up to 18 years old admitted to hospital for confirmed or suspected severe bacterial infection (SBI), in whom intravenous (IV) antibiotics are commenced, and who are expected to remain on IV antibiotics for more than 48 hours.<br><br>Conditions include (but not limited to):<br>bacteraemia, central line-associated bloodstream infections (CLABSIs), uncomplicated bone and joint infections (such as single site infection, osteomyelitis with adjacent septic arthritis or septic arthritis with adjacent osteomyelitis), discitis, empyema, pneumonia, pyelonephritis, sinusitis, retropharyngeal abscess, pyomyositis, uncomplicated culture-negative meningitis, intra-abdominal infections, lymphadenitis, cellulitis.<br><br>• First time in the BATCH trial.	• Preterm infants age <37 weeks corrected gestational age, under 72 hours old or ≥18 years of age.<br>• Children admitted moribund and not expected to survive more than 24 hours.<br>• Children with a predicted duration of IV antibiotics of less than 48 hours.<br>• Children not expected to survive at least 28 days because of a pre-existing condition.<br>• Children with bacterial meningitis*, bacterial endocarditis, or brain abscess.<br>• Children with complicated bone and joint infections**.<br>• Children receiving antibiotics for surgical prophylaxis.<br>• Children with chronic co-morbidities, such as cystic fibrosis, chronic lung disease, bronchiectasis where there is already a pre-defined length of course of antibiotics.<br>• Children who are severely immunocompromised (e.g. chemotherapy, stem cell transplant, biological therapy for inflammatory or rheumatological conditions).<br>• Children who, in the opinion of the local investigator, are unsuitable for randomisation due to high probability of requiring sustained IV therapy.<br>• Children with a presence of existing directive to withhold life-sustaining treatment.<br>• Inborn infants admitted to Neonatal Intensive Care Units (NICU), Neonatal High Dependency Units (NHDU), Special Care Baby Units (SCBU) or Postnatal wards.<br><br>*excluded due to NICE guideline on bacterial meningitis has predefined recommendations for duration of IV antibiotics.[22]

For peer review only - http://bmjopen.bmj.com/site/about/guidelines.xhtml

	** defined as chronic and/or related to a fracture or fixation device or prosthesis or implant. Chronic osteomyelitis presents six or more weeks after bone infection and is characterised by the presence of bone destruction and formation of sequestra.

For peer review only

For peer review only - http://bmjopen.bmj.com/site/about/guidelines.xhtml

Table 2 Schedule of enrolment, interventions and assessments

Data Type		Source Data	Data type	Screening	Baseline	Post randomisation until discharged home	Follow up (Day 28)	Frequency	By whom
1	Informed Consent	Consent form	-	X				Once	Site Clinical/Research team
2	Eligibility Assessment	Eligibility Case Report Form (CRF)	-	X				Once	Site research team
3	Demographics	CRF			X			Once	Research Nurse
4	Admission data	CRF	Comorbidities, preadmission antibiotic use, initial working diagnosis		X			Once	Research Nurse
5	Health-related Quality of Life	QRF	CHU9D		X		X	Twice	Patient/parent reported (over telephone or post at Day 28)
6	Randomisation	CRF	-		X			Once	Site Research team
7	Antibiotic use (IV/Oral)	Observation charts/ Medical notes	Antibiotic type, dose, duration,			X		Daily	Research Nurse
8	Blood tests including PCT	CRF/Medical notes	Routine blood tests<br>PCT results (for those in intervention arm)			X			
9	Clinical review	CRF/Medical notes	Clinical decision made and whether the algorithm was complied with			X		As required when a clinical decision has been made	

For peer review only - http://bmjopen.bmj.com/site/about/guidelines.xhtml

Data Type		Source Data	Data type	Screening	Baseline	Post randomisation until discharged home	Follow up (Day 28)	Frequency	By whom
10	Cerebrospinal fluid metrics, radiology and microbiology	CRF/Medical notes	White cell count, biochemistry. Microbiology results, radiology results			X			
11	Re-commencing of Antibiotics (IV and oral	Observation charts/ Medical notes	Antibiotic type, dose, duration, time recommenced			X		Daily	Research Nurse
12	Unscheduled Admissions	Medical notes	PICU readmissions post discharge			X		Daily	Research Nurse
13	Mortality	Medical notes	Date, description			X		If before Day 28	Research Nurse
14	Discharge	Medical notes	Date, description			X		If before Day 28	Research Nurse
15	Adverse events	Observation charts/ Medical notes	Date, type			X		Daily	Research Nurse
16	Suspected Adverse Drug Reactions (ADR)	Liverpool Causality Assessment tool	Date, description			X		Daily	Research Nurse
17	Resource Use	Questionnaire	Direct Medical costs (Inc. medication and ventilation and vasopressor) and Resource Use				X	Once	Patient/parent reported (over telephone or post)
18	SAE	SAE form			←------------------------As Required----------------------→				Research Nurse
19	Withdrawals	Withdrawal form			←------------------------As Required----------------------→				Research Nurse, Centre for Trial Research

For peer review only - http://bmjopen.bmj.com/site/about/guidelines.xhtml

Table 3 Elements of the Composite Safety Outcome

Composite element	Definition	Reason for inclusion	Expected prevalence in usual care	Potential direction of change with intervention
Unscheduled admissions/ Readmissions	Admitted/readmitted to PICU or unplanned readmission to hospital within 7 days of stopping IV antibiotics	Indicators of a deterioration and need for increased level of care	Our observation study showed 8.8% patients have admissions/re-admissions[5]	Increase
Reinstating IV antibiotic therapy	Restarting IV antibiotic (for any reason) therapy within 7 days of stopping IV therapy	Indicator of potentially inappropriate withdrawal of IV antibiotics and deterioration	de Jong study 2.9% in control arm re-started IV antibiotic[23]	Increase
Mortality	Death for any reason in the 28 days following randomisation	-	PICANet Annual report 2015: deaths on PICUs ~4% in 2012-2014[18]	Increase

For peer review only - http://bmjopen.bmj.com/site/about/guidelines.xhtml

Table 4 Combined Primary Outcome

	Antibiotic duration different (reduction in PCT group) (H1)	Antibiotic duration no different (no reduction in PCT group) H0)
Safety composite not worse in PCT group (H1)	✔	✖
Safety composite worse in PCT group (H0)	✖	✖

✔ - Intervention successful; ✖ - Intervention unsuccessful

Table 5 Summary of analyses of co-primary outcomes

	Co-primary outcomes	Analysis approach	Covariates in the model
Primary analysis	Duration of days of IV antibiotics (Intervention effect)	Cox regression (superiority test)	Trial arm and minimisation factors (site, age group)
	Adverse events (composite safety outcome)	Logistic regression (non-inferiority test)	Trial arm and minimisation factors (site, age group)
Secondary analyses	Duration of days of IV antibiotics (Intervention effect)	Kaplan Meier plot	Trial arm
		Log rank test	Trial arm
		Cox regression (assessments of suspected baseline confounders)	Covariates in the primary analysis, plus suspected baseline confounders (e.g. gender)
		Complier Average Causal Effect (CACE)	Covariates in the primary analysis, plus intervention adherence
	Adverse events (composite safety outcome)	Logistic regression (assessments of suspected baseline confounders)	Covariates in the primary analysis, plus suspected baseline confounders (e.g. gender)

Table 6 Summary of analyses of secondary outcomes

Secondary outcomes	Analysis approach	Covariates in the model
Proportion of ADR	Logistic regression	Trial arm and minimisation factors (site, age group)
Proportion of HAI		
Proportion of unscheduled readmission		
Proportion of re-commencing IV antibiotics		
Proportion of mortality		
Duration of antibiotics (IV and oral)	Cox regression	Trial arm and minimisation factors (site, age group)
Time to switch from broad to narrow spectrum antibiotics		
Time to discharge from hospital		
Average utility	Linear regression	Trial arm and minimisation factors (site, age group)

For peer review only - http://bmjopen.bmj.com/site/about/guidelines.xhtml

Figure 1 Guidelines for continuing or stopping IV antibiotics

Figure 2 Trial Schema / Participant flow

For peer review only

For peer review only - http://bmjopen.bmj.com/site/about/guidelines.xhtml

Figure 1 Guidelines for continuing or stopping IV antibiotics

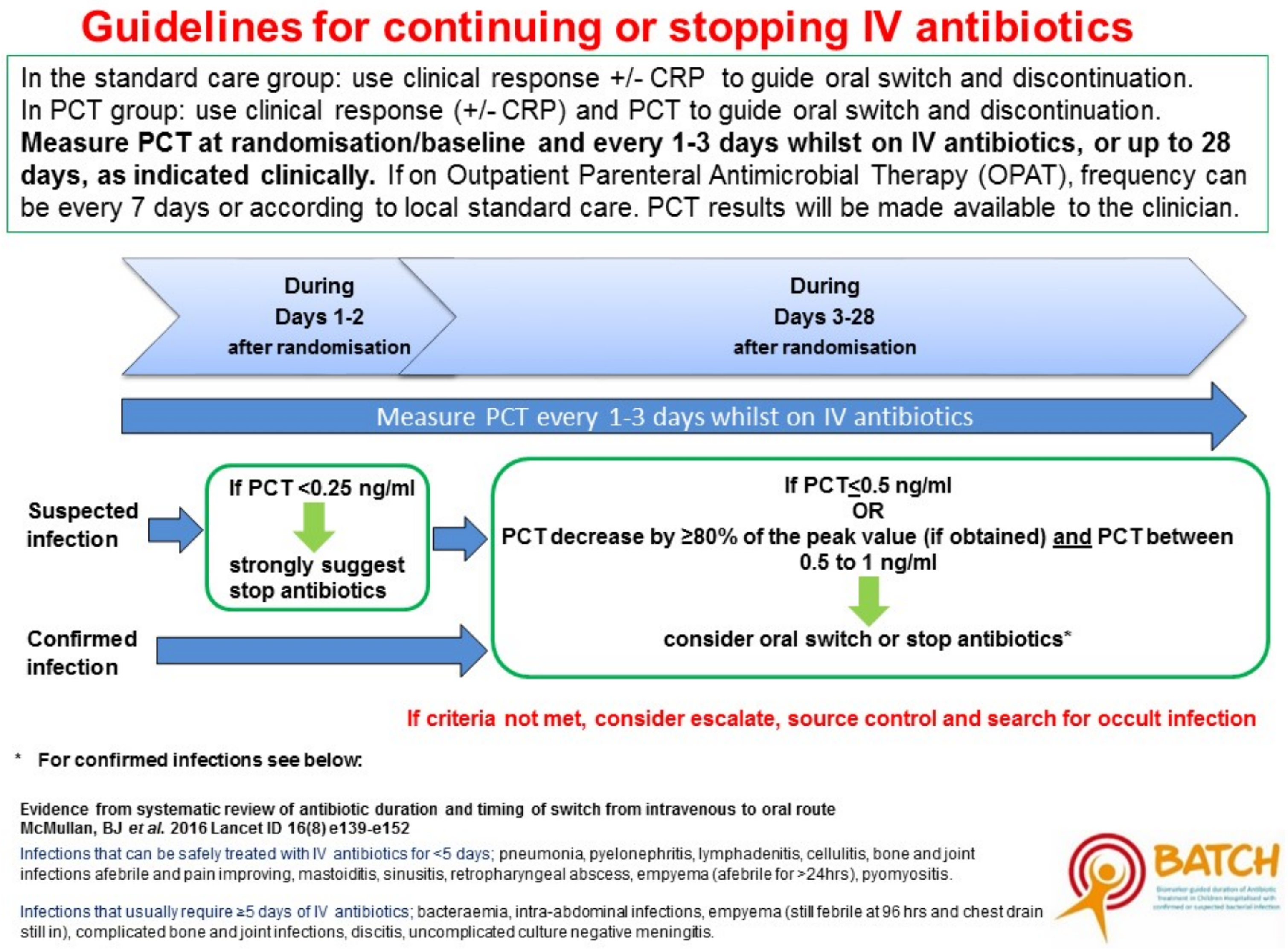

Figure 2 Trial Schema / Participant flow

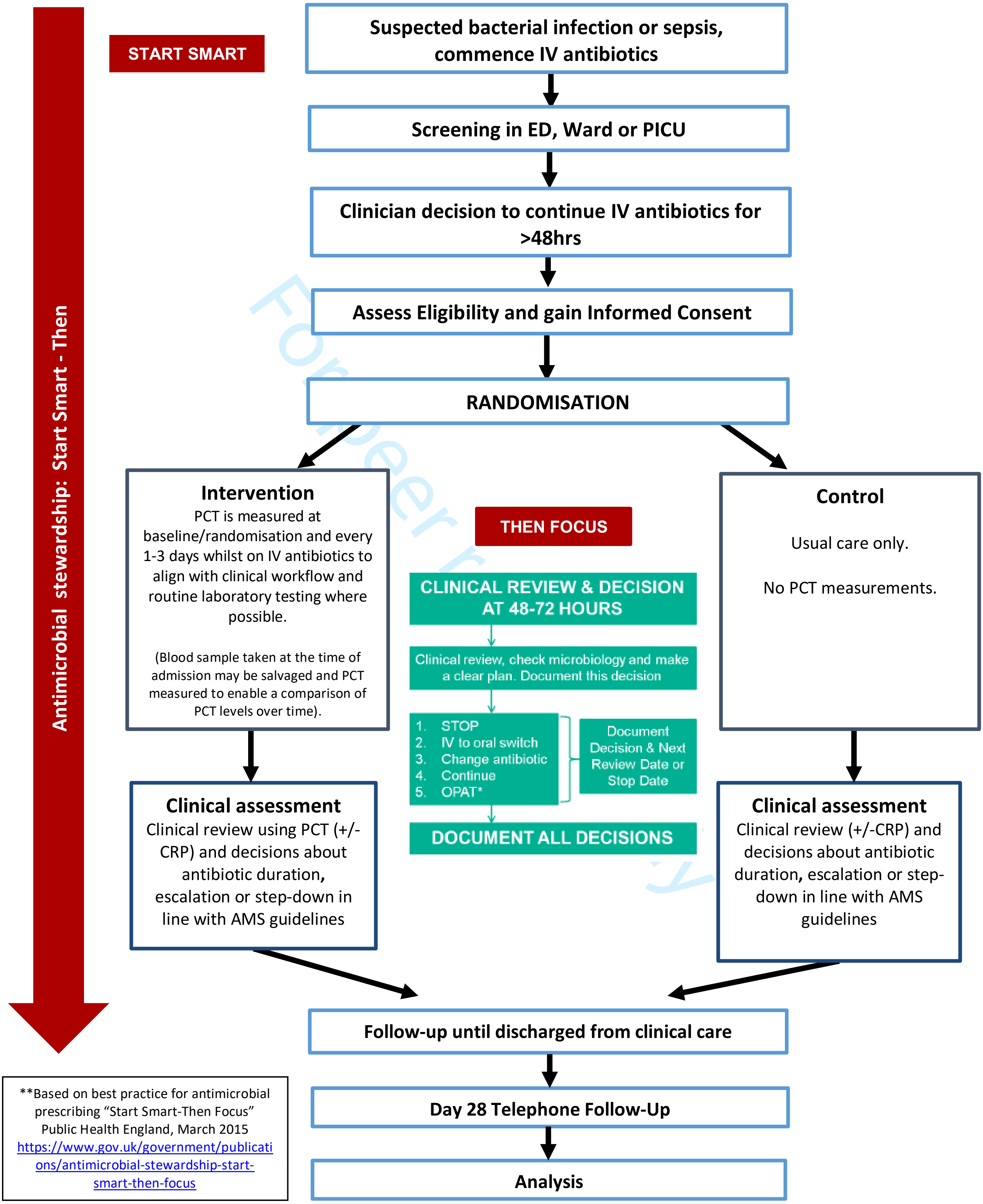

Insert NHS Trust / Health Board logo

Biomarker-guided duration of Antibiotic Treatment in Children Hospitalised with confirmed or suspected bacterial infection Trial

Information Sheet for Parents & Guardians

We are inviting you and your child to take part in this research trial run by Doctors at your hospital and scientists at the University of Liverpool, as well as some researchers from Cardiff University, Lancaster University and the University of Southampton. Before you decide if you want your child to take part, we would like you to understand why the research is being done and what it would involve for you and your child.

Please read this information sheet carefully. One of our team can go through the information sheet with you and will answer any questions you have.

What is the study about?

A daily task in hospitals is to assess ill children for the presence or absence of infection. A decision has to be made as to whether to start, stop or change antibiotics. If we can reduce antibiotic usage within hospitals, this would be an important step in combating the spread of hospital superbugs.

We are interested in studying bacterial infections in children and young people admitted to hospital. We will be assessing different markers of bacterial infection in children in hospital, to see if they can help us identify how children and young people are responding to the antibiotics that are being used to treat the infection.

For peer review only - http://bmjopen.bmj.com/site/about/guidelines.xhtml

Procalcitonin is a blood test which measures the body's response to bacterial infection, so the level is high if you have a bacterial infection, but levels reduce as you respond to antibiotics.

We will be using procalcitonin to help us decide if a patient is responding to antibiotic treatment or whether the treatment can be stopped safely.

We want to see if the use of a procalcitonin test can safely aid decisions to start, stop or change antibiotics. We are looking to recruit nearly two thousand patients aged under 18 years old to take part in a trial to help us reduce antibiotic usage safely in children and young people admitted to hospital.

We need to assess new tests of infection in a scientific manner to be sure that they improve patient care before being introduced into everyday routine practice.

Why has my child been asked to take part?

Your child's clinician thinks that your child may have a bacterial infection, and has identified that they could be eligible for the BATCH trial.

Does my child have to take part?

Your child does not have to take part in this trial. Participation in the study is entirely voluntary and you and your child are free to refuse to take part or withdraw from the trial at any time without having to give a reason. If you do decide not to take part or to withdraw from the trial, this will not affect the care that your child receives.

What will happen if my child takes part?

If you do decide to take part in the trial your child will be randomised to either have procalcitonin tests or normal care. We will try to do these procalcitonin tests at the same time as routine blood tests are being taken, however, we may need to take an additional blood test at separate time points if routine bloods are not due to be collected or there is not enough routine blood collected to perform the procalcitonin test,

There will be an equal chance of your child having the additional procalcitonin tests or receiving normal care.

For peer review only - http://bmjopen.bmj.com/site/about/guidelines.xhtml

Can I choose which part of the trial to be in?

No. To get the most reliable information about how well the procalcitonin test works we must ensure that each patient is given an equal chance of having the additional tests or not, and that this is chosen at random.

During your child's hospital stay

Your child's clinician will manage your child's care as per normal practice, however, if your child is randomised to the intervention group and has the procalcitonin tests, your child's treatment will be guided by the results of these tests. We will study how your child's procalcitonin levels relate to their response to treatment.

We will collect an additional 1ml of blood for this test. We will try to collect this when blood is being taken as part of routine care, however we may need to take an additional blood test at separate time points if routine bloods are not due to be collected or there is not enough routine blood collected to perform the procalcitonin test. All the other tests performed are those which should routinely be performed.

We will collect information on:

- whether infection is present and on the severity of their illness
- costs associated with having a child in hospital with a bacterial infection
- You will also be asked to complete some questionnaires about your child's health status.

If you do not give consent to take part in the study this will not affect the care given to your child.

What will happen to the blood sample?

Your child's samples taken whilst they are being treated for this illness will be tested in accredited laboratories at your hospital. They will be kept safe and will only be identified by a study code.
This may include samples taken when they were admitted which are normally discarded by the laboratory after the routine tests are done.

Your child's blood samples taken whilst they are being treated for this illness may also be stored for future research concerning infections and antibiotic resistance.

For peer review only - http://bmjopen.bmj.com/site/about/guidelines.xhtml

The samples will be stored **confidentially** in a secure location in a laboratory at the University of Liverpool. Your child's name will not be stored with the sample and will not be used in any publications relating to use of your child's sample(s). **You may withdraw consent for this storage and future use of your child's sample(s)**. If you withdraw consent, the sample(s) will not be used in any future studies and will be destroyed according to approved practices. We will seek ethical approval if we want to use the samples for any future research. We will not be doing any tests on human DNA now or in the future.

At Day 28 follow-up.

We will contact you 28 days after your child was recruited into the study to find out how they are. This will likely be by telephone, email or post and should take around half an hour of your time. We would also like to look at your child's medical records so that we can collect information on healthcare costs such as prescriptions, visits to the GP and so on. This is to allow us to work out if the additional test to reduce antibiotic use will save money in the NHS in the long run.

Longer term follow up using health data

After the trial, we would like to see how your child is doing in the longer term by using routinely collected health data. This is information that your GP and hospital collect when looking after your child. We will look at this information to explore the long term effects of procalcitonin test versus normal care.

The reason we want to look at this information is to assess your child's well-being and health following the trial. The research team will provide details such as name, NHS number, date of birth, postcode and gender to identify your child to NHS Digital in order for those professionals to provide relevant information about your child's ongoing health. NHS Digital will provide information on how trial participants have used different health services (e.g. how many hospital visits) and for what reasons.

NHS digital will provide data to Cardiff University. No identifiable data will be sent to the database. Instead, a study number will be assigned to each individual and this will be used to join pieces of information together. Data viewed by the research team will not be identifiable. In other words, when we look at your health information, all we will see is a database containing numbers. A Data Manager will use the study number to identify a person and join up the information. We will not know who is who in the database. The data will be held on a secure server and only the research team will have access to it.

For peer review only - http://bmjopen.bmj.com/site/about/guidelines.xhtml

Qualitative interview study

A few parents in the trial will be asked by a member of the research team to discuss the experience of their child taking part in the trial and about their child's health. If you agree, a researcher will give you some information about the additional study and what will be required. After you have had time to go through this and ask any questions you might have, you will be asked if you are happy to take part. If so you will be asked to sign a consent form to confirm that you understand this part of the study and agree to participate. If you do not take part this will not affect your involvement in the research or ongoing health care.

What are the possible advantages of taking part?

If the number of days that it is necessary to give antibiotic treatment can be safely reduced, children may benefit from the possibility of fewer days suffering from the known side effects of antibiotic treatment and a reduced risk of building up resistance to antibiotics working in the future (antimicrobial resistance). Your child will continue to receive standard clinical treatment, and their clinicians continue to decide on how long antibiotic treatment is given. By participating in this trial the information we collect about your child will help children/young people in the future.

What are the possible disadvantages and risks of taking part?

Taking part in the trial will mean that we ask you to give up some of your time during your child's hospital stay and at your child's follow-up telephone call. There are no risks for your child. In the event that something does go wrong and your child is harmed during the research and this is due to someone's negligence then you may have grounds for a legal action for compensation against Alder Hey Children's NHS Foundation Trust and The University of Liverpool, but you may have to pay your legal costs. The normal National Health Service complaints mechanisms will still be available.

Will all my information be kept confidential?

Yes, all information collected about you and your child during the course of the trial will be kept strictly confidential. Doctors involved in the care of your child will be informed about your participation in the trial, and will be kept up to date with your child's progress to ensure their safety, but they will not see any answers to trial questionnaires.

For peer review only - http://bmjopen.bmj.com/site/about/guidelines.xhtml

Your child's medical records may be examined by the research team but will be kept confidential. Your child's name (or any other identifiable information) will not be given out or appear on any publications**.**

University of Liverpool and Cardiff University will use your name, date of birth and contact details to contact you about the research study, and make sure that relevant information about the study is recorded for your care, and to oversee the quality of the study. Individuals from University of Liverpool and Cardiff University and regulatory organisations may look at your research records to check the accuracy of the research study. The only people in University of Liverpool and Cardiff University who will have access to information that identifies you/your child will be people who need to contact you to collect more information, take part in a qualitative interview, send you the results of the trial or to audit the data collection process. The people who analyse the information will not be able to identify you and will not be able to find out your name, or contact details.

University of Liverpool and Cardiff University will keep identifiable information about you/your child securely, for a minimum of 10 years after the BATCH trial has finished. This is in line with University of Liverpool policies.

All members of the trial team and regulatory authorities are trained in data protection issues. They are also bound by the terms of the General Data Protection Regulation (GDPR) (EU) 2016/679.

Who is responsible for looking after my information?

University of Liverpool is the sponsor for this study based in the United Kingdom. The University of Liverpool and Cardiff University will be using information from you and/your child's medical records in order to undertake this study and will act as the data controller for this study. This means that we are responsible for looking after your information and using it properly. Your rights to access, change or move your information are limited, as we need to manage your information in specific ways in order for the research to be reliable and accurate. If you withdraw from the study, we will keep the information about you/ your child that we have already obtained. To safeguard your rights, we will use the minimum personally-identifiable information possible.

You can find out more about how we use your information here: https://www.cardiff.ac.uk/public-information/policies-and-procedures/data-protection

For peer review only - http://bmjopen.bmj.com/site/about/guidelines.xhtml

Is my data likely to be used for future research?

When you agree to take part in a research study, the information about your health and care may be provided to researchers running other research studies in this organisation and in other organisations. These organisations may be universities, NHS organisations or companies involved in health and care research in this country or abroad. Your information will only be used by organisations and researchers to conduct research in accordance with the UK Policy Framework for Health and Social Care Research.

This information will not identify you/your child and will not be combined with other information in a way that could identify you. The information will only be used for the purpose of health and care research, and cannot be used to contact you or to affect your care. It will not be used to make decisions about future services available to you, such as insurance.

What if I, or my child do not want to carry on being part of the trial?

You can decide for your child to stop taking part in the trial at any time and without needing to give a reason. If you wish to withdraw, you can contact the trial manager or let the research nurse know next time they contact you. In order for us to understand the reasons why parents withdraw their children from the study, we may ask you why you have decided to withdraw. However, you do not have to give any reasons.

It is usual practice to keep the information we have collected to help draw conclusions about the treatment you received. If however you do not want your information used you may request that it be withdrawn from the study as well.

If you decide to stop taking part, your child's current and future medical care and the legal rights of you and your child will not be affected in any way.

What will happen to the results of the research?

We hope that the results of the trial will improve the care of many patients in the future. At the end of the trial, the results will be published in medical journals and presented at medical conferences. This will allow us to tell other doctors and healthcare professionals across the world about the results of the trial.

For peer review only - http://bmjopen.bmj.com/site/about/guidelines.xhtml

Information that identifies you or your child **will not** be presented. We can provide you with information of where to access the published trial data. A copy of the results can be sent to you if you would like.

Who has funded and approved the study?

This trial has been funded by the National Institute for Health Research Health Technology Assessment (NIHR HTA) Programme and is being managed by Cardiff University.

The study has been approved by an independent NHS Research Ethics Committee (REC) North West-Liverpool East (reference: 18/NW/0100). The committee makes sure that the study is conducted ethically to protect the safety, rights, wellbeing and dignity of the patient in line with the requirements of Clinical Trial Regulations.

What if there is a problem?

If your child experiences any side effects or complications as a result of taking part in the trial there are no compensation arrangements over and above the usual routes available to any patient treated within the NHS. If at any point you are unhappy with any aspect of the trial, please advise your child's Clinician or the research team at the Centre for Trials Research, Cardiff University (contact numbers below). If you remain unhappy and wish to formally complain, you can do this through the normal NHS complaints procedure. Taking part in the trial will not affect your legal rights.

What do I need to do now?

If you agree to consider having your child take part in the study please tell your child's clinician whether or not you are happy for your child to take part. If your child is old enough you will probably want to discuss the study with them. The research nurse can help you discuss the study with your child.

Thank you for reading this information sheet and considering participation in this trial. Our team is experienced and dedicated to doing this important trial to the highest international standards, and helping to improve the future care of patients.

For peer review only - http://bmjopen.bmj.com/site/about/guidelines.xhtml

Should you have any further questions or require further information about taking part you can contact (during normal working hours):

BATCH Trial Manager
Centre for Trials Research,
Cardiff University,
Heath Park,
Cardiff, CF14 4YS
Tel: 02920 687609
Email: BATCH@cardiff.ac.uk

Please note that this number is only for queries regarding the trial; if you have an urgent medical problem please contact your surgeon/doctor in the normal way.

The Principal Investigator for this site is:-

Insert PI details

The Research Nurse for this site is:-

Insert RN detail

For peer review only

For peer review only - http://bmjopen.bmj.com/site/about/guidelines.xhtml

BATCH: Biomarker-guided duration of Antibiotic Treatment in Children Hospitalised with confirmed or suspected bacterial infection Trial

Insert NHS Trust /Health Board logo

Site ID	Patient ID

CONSENT FORM

(Please **initial** each box and sign in full at the bottom of the page)

1. I confirm that I have read and understood the Information Sheet Information Sheet for Parents and Guardians (version 2.1, dated 31/01/2019) for the BATCH trial. I have had the opportunity to consider the information, ask questions and have had these answered satisfactorily.
2. I understand that my child's participation is voluntary and that I am free to withdraw my child at any time, without giving any reason, without my child's normal medical care or legal rights being affected.
3. I understand the trial is randomised and no one can pick which treatment my child receives. I understand that my child will be randomised to have either procalcitonin tests or usual care. If my child is allocated to the treatment arm of the trial their procalcitonin levels will be tested. This may be done as part of routine blood tests or an additional blood sample may need to be taken at separate time points if routine bloods are not due to be collected or not enough routine blood is collected to perform the procalcitonin test. I consent to these tests being performed on the samples collected during my child's illness. I understand that I will not be given the laboratory results.
4. I understand that the research team may be required to look in my child's medical notes or contact my child's GP to ask about my child's health and medications. I give permission for them to do this.
5. I understand that a member of the research team will need to contact me by telephone, email or post to complete a survey about my child's health. I give permission for them to do so.
6. I understand that a member of the research team may need to contact me to carry out an interview about my child's health and experiences in the trial (Qualitative Interviews). I give permission for them to do so.
7. I understand that information collected about my child that is held and maintained by NHS Digital and other central UK NHS bodies, may be collected from my medical records and other health-related records and looked at by the research team and responsible practitioners during the trial. I give permission for these individuals to have access to these records and for them to be used in this research on the understanding that all information will remain confidential.
8. I understand that information collected about my child (including name and address) will be held at the Centre for Trials Research, Cardiff University according to the 2018 General Data Protection Regulation (GDPR) (EU 2016/679). I understand that this information will be kept strictly confidential and that no personal information will be used in the study report or publications.
9. I understand that my child's blood samples will be stored securely for further research. Names and addresses will be removed from all samples stored for research purposes. Further ethical approval will be sought from the Ethics Committee for any future research on these samples. No DNA analysis will be carried out now or in the future.
10. I agree for my child to take part in the above trial.

Name of Child (please print):____

Name of Parent / Guardian (please print):__________________________ Signed:__________Date:___/___/___

Name of Person taking consent (please print):______________________Signed:__________Date:___/___/___

When completed, store White copy in Site File; Green for Medical Notes; Yellow for parent/carer
Please fax a copy to CTR: 02030 095405

Local Researcher: Professor Enitan Carrol, Honorary Consultant in Paediatric Infectious Diseases and Immunology, University of Liverpool and Alder Hey Children's NHS Foundation Trust

For peer review only - http://bmjopen.bmj.com/site/about/guidelines.xhtml

Reporting checklist for protocol of a clinical trial.

Based on the SPIRIT guidelines.

Instructions to authors

Complete this checklist by entering the page numbers from your manuscript where readers will find each of the items listed below.

Your article may not currently address all the items on the checklist. Please modify your text to include the missing information. If you are certain that an item does not apply, please write "n/a" and provide a short explanation.

Upload your completed checklist as an extra file when you submit to a journal.

In your methods section, say that you used the SPIRITreporting guidelines, and cite them as:

Chan A-W, Tetzlaff JM, Gøtzsche PC, Altman DG, Mann H, Berlin J, Dickersin K, Hróbjartsson A, Schulz KF, Parulekar WR, Krleža-Jerić K, Laupacis A, Moher D. SPIRIT 2013 Explanation and Elaboration: Guidance for protocols of clinical trials. BMJ. 2013;346:e7586

		Reporting Item	Page Number
**Administrative information**			
Title	#1	Descriptive title identifying the study design, population, interventions, and, if applicable, trial acronym	1
Trial registration	#2a	Trial identifier and registry name. If not yet registered, name of intended registry	1
Trial registration: data set	#2b	All items from the World Health Organization Trial Registration Data Set	1
Protocol version	#3	Date and version identifier	17
Funding	#4	Sources and types of financial, material, and other support	19
Roles and responsibilities: contributorship	#5a	Names, affiliations, and roles of protocol contributors	1 and 19

For peer review only

Roles and responsibilities: sponsor contact information	#5b	Name and contact information for the trial sponsor	19
Roles and responsibilities: sponsor and funder	#5c	Role of study sponsor and funders, if any, in study design; collection, management, analysis, and interpretation of data; writing of the report; and the decision to submit the report for publication, including whether they will have ultimate authority over any of these activities	19
Roles and responsibilities: committees	#5d	Composition, roles, and responsibilities of the coordinating centre, steering committee, endpoint adjudication committee, data management team, and other individuals or groups overseeing the trial, if applicable (see Item 21a for data monitoring committee)	17
**Introduction**			
Background and rationale	#6a	Description of research question and justification for undertaking the trial, including summary of relevant studies (published and unpublished) examining benefits and harms for each intervention	2-5
Background and rationale: choice of comparators	#6b	Explanation for choice of comparators	7
Objectives	#7	Specific objectives or hypotheses	5
Trial design	#8	Description of trial design including type of trial (eg, parallel group, crossover, factorial, single group), allocation ratio, and framework (eg, superiority, equivalence, non-inferiority, exploratory)	6
**Methods: Participants, interventions, and outcomes**			
Study setting	#9	Description of study settings (eg, community clinic, academic hospital) and list of countries where data will be	6

For peer review only

		collected. Reference to where list of study sites can be obtained	
Eligibility criteria	#10	Inclusion and exclusion criteria for participants. If applicable, eligibility criteria for study centres and individuals who will perform the interventions (eg, surgeons, psychotherapists)	6 and Table 1
Interventions: description	#11a	Interventions for each group with sufficient detail to allow replication, including how and when they will be administered	7
Interventions: modifications	#11b	Criteria for discontinuing or modifying allocated interventions for a given trial participant (eg, drug dose change in response to harms, participant request, or improving / worsening disease)	n/a
Interventions: adherance	#11c	Strategies to improve adherence to intervention protocols, and any procedures for monitoring adherence (eg, drug tablet return; laboratory tests)	n/a
Interventions: concomitant care	#11d	Relevant concomitant care and interventions that are permitted or prohibited during the trial	n/a
Outcomes	#12	Primary, secondary, and other outcomes, including the specific measurement variable (eg, systolic blood pressure), analysis metric (eg, change from baseline, final value, time to event), method of aggregation (eg, median, proportion), and time point for each outcome. Explanation of the clinical relevance of chosen efficacy and harm outcomes is strongly recommended	7-8
Participant timeline	#13	Time schedule of enrolment, interventions (including any run-ins and washouts), assessments, and visits for participants. A schematic diagram is highly recommended (see Figure)	10-11 and Figure 2
Sample size	#14	Estimated number of participants needed to achieve study objectives and how it was determined, including clinical and statistical assumptions supporting any sample size calculations	12
Recruitment	#15	Strategies for achieving adequate participant enrolment to reach target sample size	9

For peer review only

**Methods: Assignment of interventions (for controlled trials)**			
Allocation: sequence generation	#16a	Method of generating the allocation sequence (eg, computer-generated random numbers), and list of any factors for stratification. To reduce predictability of a random sequence, details of any planned restriction (eg, blocking) should be provided in a separate document that is unavailable to those who enrol participants or assign interventions	10
Allocation concealment mechanism	#16b	Mechanism of implementing the allocation sequence (eg, central telephone; sequentially numbered, opaque, sealed envelopes), describing any steps to conceal the sequence until interventions are assigned	10
Allocation: implementation	#16c	Who will generate the allocation sequence, who will enrol participants, and who will assign participants to interventions	10
Blinding (masking)	#17a	Who will be blinded after assignment to interventions (eg, trial participants, care providers, outcome assessors, data analysts), and how	13
Blinding (masking): emergency unblinding	#17b	If blinded, circumstances under which unblinding is permissible, and procedure for revealing a participant's allocated intervention during the trial	n/a
**Methods: Data collection, management, and analysis**			
Data collection plan	#18a	Plans for assessment and collection of outcome, baseline, and other trial data, including any related processes to promote data quality (eg, duplicate measurements, training of assessors) and a description of study instruments (eg, questionnaires, laboratory tests) along with their reliability and validity, if known. Reference to where data collection forms can be found, if not in the protocol	10-11 and Table 2

For peer review only

Data collection plan: retention	#18b	Plans to promote participant retention and complete follow-up, including list of any outcome data to be collected for participants who discontinue or deviate from intervention protocols	11
Data management	#19	Plans for data entry, coding, security, and storage, including any related processes to promote data quality (eg, double data entry; range checks for data values). Reference to where details of data management procedures can be found, if not in the protocol	9
Statistics: outcomes	#20a	Statistical methods for analysing primary and secondary outcomes. Reference to where other details of the statistical analysis plan can be found, if not in the protocol	13
Statistics: additional analyses	#20b	Methods for any additional analyses (eg, subgroup and adjusted analyses)	14
Statistics: analysis population and missing data	#20c	Definition of analysis population relating to protocol non-adherence (eg, as randomised analysis), and any statistical methods to handle missing data (eg, multiple imputation)	14
**Methods: Monitoring**			
Data monitoring: formal committee	#21a	Composition of data monitoring committee (DMC); summary of its role and reporting structure; statement of whether it is independent from the sponsor and competing interests; and reference to where further details about its charter can be found, if not in the protocol. Alternatively, an explanation of why a DMC is not needed	17
Data monitoring: interim analysis	#21b	Description of any interim analyses and stopping guidelines, including who will have access to these interim results and make the final decision to terminate the trial	13
Harms	#22	Plans for collecting, assessing, reporting, and managing solicited and spontaneously reported adverse events and other unintended effects of trial interventions or trial conduct	12

For peer review only

Auditing	#23	Frequency and procedures for auditing trial conduct, if any, and whether the process will be independent from investigators and the sponsor	9
**Ethics and dissemination**			
Research ethics approval	#24	Plans for seeking research ethics committee / institutional review board (REC / IRB) approval	17
Protocol amendments	#25	Plans for communicating important protocol modifications (eg, changes to eligibility criteria, outcomes, analyses) to relevant parties (eg, investigators, REC / IRBs, trial participants, trial registries, journals, regulators)	17
Consent or assent	#26a	Who will obtain informed consent or assent from potential trial participants or authorised surrogates, and how (see Item 32)	9
Consent or assent: ancillary studies	#26b	Additional consent provisions for collection and use of participant data and biological specimens in ancillary studies, if applicable	9
Confidentiality	#27	How personal information about potential and enrolled participants will be collected, shared, and maintained in order to protect confidentiality before, during, and after the trial	9
Declaration of interests	#28	Financial and other competing interests for principal investigators for the overall trial and each study site	19
Data access	#29	Statement of who will have access to the final trial dataset, and disclosure of contractual agreements that limit such access for investigators	n/a
Ancillary and post trial care	#30	Provisions, if any, for ancillary and post-trial care, and for compensation to those who suffer harm from trial participation	n/a
Dissemination policy: trial results	#31a	Plans for investigators and sponsor to communicate trial results to participants, healthcare professionals, the public, and other relevant groups (eg, via publication, reporting in results databases, or other data sharing arrangements), including any publication restrictions	18

For peer review only

Dissemination policy: authorship	#31b	Authorship eligibility guidelines and any intended use of professional writers	18
Dissemination policy: reproducible research	#31c	Plans, if any, for granting public access to the full protocol, participant-level dataset, and statistical code	n/a
**Appendices**			
Informed consent materials	#32	Model consent form and other related documentation given to participants and authorised surrogates	Supplementary material
Biological specimens	#33	Plans for collection, laboratory evaluation, and storage of biological specimens for genetic or molecular analysis in the current trial and for future use in ancillary studies, if applicable	7

Notes:

- 10: 6 and Table 1
- 13: 8-9 and Figure 2
- 18a: 8-9 and Table 2 The SPIRIT checklist is distributed under the terms of the Creative Commons Attribution License CC-BY-ND 3.0. This checklist was completed on 29. November 2020 using https://www.goodreports.org/, a tool made by the EQUATOR Network in collaboration with Penelope.ai

For peer review only

BMJ Open

Biomarker-guided duration of Antibiotic Treatment in Children Hospitalised with confirmed or suspected bacterial infection (BATCH): Protocol for a randomised controlled trial.

Journal:	*BMJ Open*
Manuscript ID	bmjopen-2020-047490.R2
Article Type:	Protocol
Date Submitted by the Author:	23-Nov-2021
Complete List of Authors:	Waldron, Cherry-Ann; Cardiff University, Centre for Trials Research<br>Thomas-Jones, Emma; Cardiff University, Centre for Trials Research<br>Bernatoniene, Jolanta; Bristol Royal Hospital for Children, Paediatric Infectious Disease Department<br>Brookes-Howell, Lucy; Cardiff University Centre for Trials Research<br>Faust, Saul; University of Southampton, UK, NIHR Wellcome Trust Clincial Research Facility<br>Harris, Debbie; Cardiff University, Centre for Trials Research<br>Hinds, Lucy ; Sheffield Children's NHS Foundation Trust, Paediatrics<br>Hood, Kerenza; Cardiff University Centre for Trials Research<br>Huang, Chao; Hull York Medical School,<br>Mateus, Ceu; University of Lancaster, Division of Health Research<br>Pallmann, Philip; Cardiff University Centre for Trials Research, College of Biomedical and Life Sciences, Centre for Trials Research<br>Patel, Sanjay; Southampton Children's Hospital<br>Paulus, Stephane; University of Oxford, Paediatrics; University of Liverpool Institute of Infection and Global Health,<br>Peak, Matthew; Alder Hey Children's NHS Foundation Trust, R&D<br>Powell, Colin; Children's Hospital for Wales, Paediatrics; Sidra Medicine<br>Preston, Jenny; Alder Hey Children's NHS Foundation Trust<br>Carrol, Enitan; University of Liverpool, Institute of Infection, Veterinary and Ecological Sciences
**Primary Subject Heading**:	Paediatrics
Secondary Subject Heading:	Infectious diseases
Keywords:	INFECTIOUS DISEASES, Paediatric intensive & critical care < INTENSIVE & CRITICAL CARE, Diagnostic microbiology < INFECTIOUS DISEASES, Public health < INFECTIOUS DISEASES

SCHOLARONE™
Manuscripts

Biomarker-guided duration of **A**ntibiotic **T**reatment in **C**hildren **H**ospitalised with confirmed or suspected bacterial infection (BATCH): Protocol for a randomised controlled trial.

Waldron CA[1*], Thomas-Jones E[1], Bernatoniene J[2], Brookes-Howell L[1], Faust S.N[3], Harris D[1], Hinds L[4], Hood K[1], Huang C[5], Mateus C[6], Pallmann P[1], Patel S[7], Paulus S[8], Peak M[9], Powell C[10], Preston J[9], Carrol ED[11]

[*]Corresponding author
Dr Cherry-Ann Waldron, Centre for Trials Research, Cardiff University, 7th Floor, Neuadd Meirionnydd, Heath Park, Cardiff, CF14 4YS, Tel: +44(0)29 20687609, Email: waldronc@cardiff.ac.uk

1 Centre for Trials Research, Cardiff University, Cardiff, UK
2 University Hospitals Bristol NHS Foundation Trust
3 University Hospital Southampton NHS Foundation Trust, Southampton, UK
4 Sheffield Children's NHS Foundation Trust, Sheffield, UK
5 Hull York Medical School, Hull, University of Hull, UK
6 Division of Health Research, Lancaster University, Lancaster, UK
7 Faculty of Medicine, University of Southampton, University Hospital Southampton NHS Foundation Trust, Southampton
8 Oxford University Hospitals NHS Foundation Trust, Oxford, UK
9 Alder Hey Children's NHS Foundation Trust, Liverpool, UK
10 Child Health, School of Medicine, Cardiff University, UK / Sidra Medicine, Doha, Qatar
11 University of Liverpool, Liverpool, UK

Key words: Paediatrics, Bacterial infection, Procalcitonin, Antibiotic duration

ABSTRACT

Introduction

Procalcitonin (PCT) is a biomarker more specific for bacterial infection and responds quicker than other commonly used biomarkers such as C-Reactive Protein, but is not routinely used in the NHS. Studies mainly in adults show that using PCT to guide clinicians may reduce antibiotic use, reduce hospital stay, with no associated adverse effects such as increased rates of hospital re-admission, incomplete treatment of infections, relapse or death. A review conducted for NICE recommends further research on PCT testing to guide antibiotic use in children.

For peer review only - http://bmjopen.bmj.com/site/about/guidelines.xhtml

Methods and analysis

BATCH is a multi-centre, prospective, two-arm, individually RCT with a 28 day follow up and internal pilot. The intervention is a PCT-guided algorithm used in conjunction with best practice. The control arm is best practice alone. We plan to recruit 1942 children, aged between 72 hours and up to 18 years old, who are admitted to hospital and being treated with intravenous antibiotics for suspected or confirmed bacterial infection. Co-primary outcomes are duration of antibiotic use and a composite safety measure. Secondary outcomes include time to switch from broad to narrow spectrum antibiotics, time to discharge, adverse drug reactions, health utility and cost effectiveness. We will also perform a qualitative process evaluation. Recruitment commenced in June 2018 and paused briefly between March-May 2020 due to the COVID-19 pandemic.

Ethics and dissemination

The trial protocol was approved by the HRA and NHS REC (North West Liverpool East REC reference 18/NW/0100). We will publish the results in international peer-reviewed journals and present at scientific meetings.

Trial registration: ISRCTN 11369832

Strengths and limitations of this trial

- Trial will evaluate both safety and effectiveness of PCT to guide antibiotic duration in children hospitalised with suspected or confirmed bacterial infection.
- Randomised controlled trial with multicentre design including patients from hospital sites in England and Wales.Efficient trial design with co-primary endpoints and including a health economic analysis and qualitative process evaluation.
- Due to the type of intervention it is not possible to blind patients and clinicians.
- Potential shift in patient population following a recruitment break due to COVID-19 pandemic will be addressed with sensitivity analyses.

INTRODUCTION

Sepsis is defined as life-threatening organ dysfunction caused by a dysregulated host response to infection.[1] Sepsis causes many non-specific symptoms and signs that can also be

For peer review only - http://bmjopen.bmj.com/site/about/guidelines.xhtml

caused by a large number of conditions that may or may not be due to infection, and that may or may not require immediate or urgent treatment. Sepsis is usually caused by bacteria, although viral and fungal causes do occur. The problem for clinicians is the difficulty in distinguishing bacterial sepsis from other conditions presenting with similar non-specific signs and symptoms. Prompt administration of antibiotics reduces mortality by half,[2] but indiscriminate antibiotic use unnecessarily increases antimicrobial resistance (AMR), resulting in increased costs in hospitalised patients,[3 4] which represents a significant resource burden in the NHS. Not all children admitted with bacterial infection will meet the criteria for sepsis, but they could still have serious infection, requiring IV antibiotics for several days.

Biomarker blood tests currently used in the NHS, such as C-Reactive Protein (CRP) do not reliably differentiate beween severe bacterial infection (SBI) (defined previously [5]) and inflammation, and show a delayed response to bacterial infection. PCT is a biomarker released in response to inflammatory stimuli including bacterial infections, with very high levels produced in SBI.[6] In contrast to CRP, PCT rises early and peaks early, and falls rapidly in response to effective antimicrobial therapy. This makes blood PCT potentially a better biomarker for monitoring progression of SBI and response to antimicrobial therapy, and for facilitating clinical decision making by informing initiation, change or discontinuation of antimicrobial therapy. It supports the aims of the Department of Health Five Year Antimicrobial Resistance Strategy 2019-2024 of conserving and stewarding the effectiveness of existing antimicrobials by ensuring that antibiotics are used responsibly and less often (i.e. antimicrobial stewardship (AMS)).[7]

There is strong evidence for introducing paediatric AMS programmes in hospital settings, in terms of reduced antibiotic use, improved quality of prescribing and cost-savings. Long-term and sustainable reductions in antimicrobial prescribing and a reduction of resistance rates at a population level have been achieved by the implementation of nationally coordinated, whole-system approaches, with no evidence of an increase in the rate of serious infection or bacterial complications.[8] We recently published a large study prospectively assessing the performance of multiple biomarkers of serious bacterial infection (SBI) in a heterogeneous cohort of critically ill children and uniquely profiled longitudinal biomarker changes.

For peer review only - http://bmjopen.bmj.com/site/about/guidelines.xhtml

Longitudinal profiles for PCT showed the greatest percentage drop in values over the first seven days of therapy in children with SBI, suggesting that PCT might be useful in guiding duration of antimicrobial therapy in children [9].

National Institute for Health and Care Excellence (NICE) guidance on AMS (https://www.nice.org.uk/guidance/ng15) recommends decision support systems as an AMS intervention. The use of a PCT-based algorithm to guide antibiotic stopping or escalation is one such decision support system which can be used. The AMS guidelines made research recommendations including RCTs to determine whether short or long courses of antibiotics reduce AMR and whether using point-of-care tests is clinically and cost effective when prescribing antimicrobials in children with respiratory tract infections. The BATCH trial described in this paper is aligned with these recommendations in seeking to evaluate if PCT-guided management can result in shorter courses of antibiotics whilst not increasing the risk of adverse outcomes to paediatric patients.

A systematic review and cost-effectiveness analysis conducted on behalf of NICE (https://www.nice.org.uk/guidance/dg18) evaluated PCT testing to guide antibiotic therapy for the treatment of sepsis in intensive care settings and for suspected bacterial infection in emergency department (ED) settings in adults and children [10]. It concluded that addition of a PCT algorithm to the information used to guide antibiotic treatment may reduce antibiotic exposure in adults in intensive care unit (ICU) settings and in the ED without any adverse consequences and may also be associated with reductions in the length of hospital and ICU stay in adults. Evidence was not found on the effectiveness using a PCT algorithm to guide antibiotic treatment for children with suspected or confirmed SBI admitted from emergency care. None of the identified studies were conducted in the UK, and it was not clear whether the control arms of these studies were representative of standard practice in the UK. Therefore, the report recommended further studies to adequately assess the effectiveness of adding PCT algorithms to the information used to guide antibiotic treatment in adults and children with suspected or confirmed SBI in ICU settings and in adults and children with suspected bacterial infection in ED settings. It states that further studies are needed particularly for children, where data are currently lacking, and research examining (short-

For peer review only - http://bmjopen.bmj.com/site/about/guidelines.xhtml

term) health-state utility values in the UK for adults and children with confirmed or suspected SBI in the ICU and ED.

A recent systematic review and meta-analysis of antibiotic duration for bacterial infections in children demonstrated that IV to oral switch can occur earlier than previously recommended.[11] The authors produced recommendations for antibiotic duration and IV to oral switch to support clinical decision making, and recommend prospective research on optimal antibiotic durations. The lack of good evidence on recommended duration of antibiotic therapy leads to an overuse of antibiotics, contributing to the development of AMR. Combating AMR has been identified as a national and global priority. Shorter courses of antibiotic therapy would be associated with reductions in adverse effects for patients, and reductions in healthcare resource utilisation. Results from this research will inform recommendations relating to the duration of antibiotic use in future guideline updates including NICE sepsis guidelines.

Aims and Objectives

The BATCH trial aims to improve AMS in hospitalised children with suspected or confirmed bacterial infection, by reducing antibiotic duration with guidance from an additional PCT laboratory test.

Primary Objective

To determine if the addition of PCT testing to current best practice based on the NICE AMS guidelines can safely allow a reduction in duration of intravenous antibiotic therapy in hospitalised children with suspected or confirmed bacterial infection compared to current best practice alone.

To meet this objective specifically, we will assess;

- Duration of IV antibiotics
- Unscheduled admissions to PICU with infective diagnosis
- Readmissions to PICU with infective diagnosis
- Unscheduled readmissions with infective diagnosis within a week of stopping IV antibiotics

For peer review only - http://bmjopen.bmj.com/site/about/guidelines.xhtml

For peer review only

- Re-commencing IV antibiotics for the same infection within a week of stopping IV antibiotics
- Mortality

Secondary Objectives

To assess the effect of additional PCT testing to AMS best practice on:

- total duration of oral and IV antibiotics,
- time to switch from broad spectrum to narrow spectrum antibiotics,
- time to discharge from hospital,
- hospital acquired infection,
- suspected adverse drug reactions,
- cost of hospital episode,
- health related quality of life,

Also to provide detailed understanding of parent and health professionals attitudes to and experiences of, participating in the BATCH RCT.

METHODS AND ANALYSIS

Design

This is a multi-centre, prospective, two-arm, individually randomised controlled trial. An internal pilot will assess the site and participant absolute recruitment and consent rates, the proportion of participants undergoing PCT assessments and the ability to collect the primary outcome data. Recruitment commenced in June 2018 and paused briefly between March-May 2020 due to the COVID-19 pandemic.

Participants

Children admitted to hospital with suspected or confirmed SBI (diagnosed by the clinical team) and commenced on IV antibiotics, in whom IV antibiotics are likely to be continued for more than 48 hours. Recruited from paediatric wards or Paediatric Intensive Care Units

For peer review only - http://bmjopen.bmj.com/site/about/guidelines.xhtml

(PICUs) within children's hospitals and general hospitals in the UK (approximately n=10). The eligibility criteria are described in Table 1.

Intervention

In children randomised to the intervention arm, a blood sample will be sent to the hospital laboratory for a PCT test at baseline/randomisation and every 1-3 days whilst still on IV antibiotics to align with clinical workflow and routine laboratory testing where possible. This includes instances where IV antibiotics are restarted for the same infection (up to day 28 post-randomisation). An additional 1ml (minimum 0.5ml) lithium heparin samples will be collected for PCT analysis.

PCT tests will be performed on a bioMérieux VIDAS® platform. It is a prerequisite that participating sites have access this platform to take part in the trial. This is a semiautomated immunoassay system based on Enzyme Linked Fluorescent Assay principles. Calibration is performed in line with manufacturer's guidelines. It is simple and flexible to use and gives results in 20 minutes. It requires 200µl of plasma or serum and can be run on a sample sent for routine biochemistry after the routine tests have been performed.

PCT results feed into an algorithm where thresholds have been defined by previous data (Figure 1). The algorithm provides both definitive guidelines, e.g. stop antibiotics if PCT <0.25 ng/mL, and advisory guidelines, e.g. consider oral switch if PCT decreased by $\geq$80%. Clinicians can overrule the algorithm if they feel it is appropriate to do so. The algorithm values are based on our now published work which is the largest study to prospectively assess the performance of multiple biomarkers of SBI in a heterogeneous cohort of critically ill children and uniquely profiles longitudinal biomarker changes within the cohort. [9]

Children in the control arm will not have the PCT test performed.

Primary Outcome measure

The trial will use a co-primary outcome of antibiotic use and safety.

1) Antibiotic usage is defined as the number of days IV antibiotics used.

For peer review only - http://bmjopen.bmj.com/site/about/guidelines.xhtml

2) Safety is defined as the absence of all of the following:
    - Unscheduled admissions/re-admissions (to include re-admission within 7 days of discharge with infective diagnosis, unscheduled re-admission to PICU with infective diagnosis, or admission to PICU with infective diagnosis),
    - Re-treatment for same condition within 7 days of stopping IV antibiotics (re-starting IV antibiotics which have been stopped),
    - Death.

Secondary Outcome measures

- Total duration of antibiotics (IV and oral).
- Time to switch from broad spectrum to narrow spectrum antibiotics.
- Time to discharge from hospital.
- Suspected Adverse Drug Reactions (ADR) (categorised using the Liverpool Causality Assessment Tool comprising ten modified questions with their dichotomous responses into a flowchart to arrive at one of four outcome categories: 'definite', 'probable', 'possible', or 'unlikely'. It has high interrater agreement). [12 13]
-
- Cost of hospital episode.
- Hospital Acquired Infection (HAI) as defined by the clinical team up to Day 28.
- Health utility as measured by the Child Health Utility questionnaire (CHU9D) [14] up to Day 28.

Internal Pilot

An internal pilot phase will be conducted over the first eight months of the recruitment period with six lead sites. Predefined progression criteria will be used to assess feasibility to progress to the full trial, such as site and patient absolute recruitment and consent rate, proportion of patients undergoing PCT assessments and the ability to collect primary outcome data.

Trial procedures

Data Collection

For peer review only - http://bmjopen.bmj.com/site/about/guidelines.xhtml

All data collection will be by electronic data capture using a bespoke database developed by the Centre for Trials Research (CTR) and hosted by Cardiff University secure servers. It is encrypted and accessed by individual username and password. Paper copies of all case report forms will be available. Essential documents will be kept securely in a locked cupboard, and at the end of the trial, will be archived at an approved external storage facility for 10 years.

Data Management

Details of data management procedures (such as checking for missing, illegible or unusual values (range checks)) will be specified in the BATCH Data Management Plan. Details of Monitoring procedures will be specified in the BATCH Monitoring Plan.

Identification and screening

Identification of potential participants will be by the clinical care team, or the clinical members of the research team involved in care of children on the ward, or the general paediatric or infectious diseases teams involved in care of children on the ward. This includes a member of the research team visiting the wards where children with SBI are admitted to assess eligibility and screening admissions lists.

Informed consent

Informed consent will be obtained by those suitably trained and on the delegation log. Parent/carer(s) of children (or the child if over the age of 16 or Gillick competent) will be given a participant information sheet (Supplementary file 1) about the trial and will have time to consider before being asked to sign the consent form (Supplementary file 2). The consent form includes storing samples for future research. Age appropriate information sheets will also be provided for children who are old enough to use them, and those deemed to have capacity will be asked to sign an age appropriate assent form, additional to their parent/carer's consent.

For peer review only - http://bmjopen.bmj.com/site/about/guidelines.xhtml

Once consented, participants will be allocated a unique identification number (participant ID). Separate informed consent will be taken for participation in the qualitative data collection.

Withdrawal

Participants may withdraw consent to participate in any aspect of the trial, at any time. Declining to participate or withdrawing from the trial will not affect the care of the child.

Randomisation and enrolment

Children will only be randomised if the clinician expects IV antibiotics will be prescribed for longer than 48 hours. This will typically be between 20-48 hours after admission, to fit with clinical work flow of ward rounds and phlebotomy times for routine blood tests.

Participants will be allocated to the trial arms in a 1:1 ratio using minimisation[15] via a secure (24-hour) web-based randomisation programme controlled centrally by the CTR. The covariates whose imbalance is to be minimised are age group and site. A random element will be added to the minimisation algorithm (as described in [15]) to reduce the risk of predictability and subversion. Full details are provided in the BATCH randomisation strategy. To further minimise the risk of subversion, the specific age groups and the random element will not be disclosed to those involved in recruiting participants until the end of the recruitment period.

All participants will be enrolled in the trial from the date of randomisation until Day 28 follow up. Participants will be assessed until they are discharged from clinical care. Figure 2 shows the trial schema.

During Hospitalisation

Outcome data described in Table 2 will be recorded daily by the research nurse for all recruited participants (up to and including Day 28, or until discharge). Observation and

For peer review only - http://bmjopen.bmj.com/site/about/guidelines.xhtml

medication charts and medical notes will be reviewed. Assessments include antibiotic use, routine test results, PCT measurements and clinician adherence to the algorithm.

28 day follow up

At Day 28 (+2 week time window) parents will be contacted by telephone or email to ask about their child's healthcare resource use (e.g. hospital admissions, other prescribed medicines, over-the-counter medicines, GP attendance), direct non-medical costs (i.e. travel costs, child care costs, expenses incurred while in hospital, self-reported lost earnings) and the child's health related quality of life (i.e. CHU9D). If efforts to contact them by phone or email are unsuccessful, a questionnaire booklet will be posted out with a pre-paid envelope for return.

Public and patient involvement

Patient and Public Involvement (PPI) will be sought throughout the research process, from conceptualisation to dissemination. An example of this is the active involvement of the Liverpool GenerationR Young People's Advisory Group (YPAG) in contributing to the design of this research.[16] The group consists of 24 young people aged between 8 to 21 year olds who have worked with several researchers exploring the topic of developing tests to rapidly detect or diagnose SBI in children. YPAG members are well aware of the problems associated with diagnosing and treating sepsis and have discussed at length the issues associated with AMR and the need to educate young people and families about the misuse of antibiotics.

We will involve the Liverpool GenerationR YPAG, throughout the duration of the trial. The group will advise on children's information sheets and the production of educational materials for young people and families on the most appropriate use of antibiotics. We will invite parents and young people to contribute actively to dissemination events, including

For peer review only - http://bmjopen.bmj.com/site/about/guidelines.xhtml

presenting parents' and young peoples' views and stories. Parent representatives will also be invited to attend Trial Management and Steering Group Meetings. Members of the YPAG and parents will be trained by our PPI Lead.

Safety Reporting

Events described in table 3 (i.e. hospitalisation and death) are primary outcomes of the trial and are recorded as part of primary outcome data collection, and therefore are not subject to expedited reporting. Serious adverse events will be reported if the event results in persistent or significant disability or incapacity or consists of a congenital anomaly or birth defect. An assessment of causality between the event and the trial intervention will be carried out by the Principal Investigator or delegated clinician, and then independently by a clinical reviewer who will also assess expectedness. If the clinical reviewer classifies the event as probably or definitely caused by the intervention, it will be classified as a serious adverse reaction.

Sample Size

Two co-primary outcomes (IV antibiotics duration and a composite safety outcome) are defined in this trial[17] and the overall sample size is determined by both.

The focus for the intervention is on moving the step down from IV to oral therapy earlier, and therefore the time until this step down is the primary outcome on antibiotic usage, and the trial is powered to detect if PCT-directed care is *superior* to standard care on time until switch from IV antibiotics. The size of potential shortening of time to detect an effect has been taken from a systematic review.[10] The safety co-primary (table 3) is a composite measure reflecting various outcomes which represent deterioration or lack of clinical response in the child, and would be expected to increase if IV antibiotics were being withdrawn inappropriately early.

For peer review only - http://bmjopen.bmj.com/site/about/guidelines.xhtml

In terms of IV antibiotic duration, a one day reduction[18] in antibiotics from an estimated median of 5 days in the control arm (from our observation data[5]) demonstrates a hazard ratio of 1.25. At a 5% significance level with 90% power, 844 participants with observed IV antibiotics duration are needed. In terms of the event rates of safety elements, an observational study a showed a re-admission rate of 15% at Day 28.[19] In critically ill patients, up to 3% reinstating IV antibiotic therapy rate, and 3% mortality were reported.[10 18 20] With some overlaps considered, we estimate around 15% overall rate of our composite safety outcome. The SAPS trial in adults used a non-inferiority margin of 8% for mortality.[10] Given the lower expected rate of safety outcomes in this population we have chosen a similar relative non-inferiority bound of 5%. This means increases in the composite safety measure of less than 5% (from 15 to 20%) using PCT guided therapy would be considered as not inferior. With a one-sided significance level of 0.05 and 90% power we would need 1748 participants to test non-inferiority. Overall, with 1748 effectively recruited participants, we would have 99% power to detect antibiotic duration decrease and 90% power to test non-inferiority in safety separately. Assuming that these two co-primary outcomes are independent, this would give us at least 89% power for the combined analysis.[21] By considering 10% loss to follow-up for the primary outcomes, our final targeted sample size is inflated to 1942 as achievable.

STATISTICAL ANALYSES

Primary Outcome

Our primary analysis of co-primary outcomes will be intention to treat and will compare between trial arms: a) the duration of days of IV antibiotics following randomisation using Cox regression; and b) the rate of adverse events using logistic regression, with a one-sided 95% confidence interval constructed to assess non-inferiority. This analysis will control for balancing factors in the randomisation. A positive conclusion will only be made if both a decrease in IV antibiotic duration AND non-inferiority in safety can be demonstrated (Table 4). We will assess if heterogeneity among centres exists and fit a two-level model with random centre effects if confirmed. Adherence to the PCT algorithm will be recorded, and per-protocol and complier average causal effect (CACE) analysis will be undertaken to test the treatment effects to patients with fully utilised PCT algorithm (Table 5). No interim

For peer review only - http://bmjopen.bmj.com/site/about/guidelines.xhtml

analysis will be performed. The Trial Statistician will be blinded to the study group allocation.

Secondary Outcomes

For secondary outcomes, differences in the proportion of ADR, HAI, unscheduled readmission, re-commencing IV antibiotics and 28-day mortality will be assessed separately by logistic regression models. We will report the median (interquartile range [IQR]) of the total duration of antibiotics (IV and oral), time to switch from broad to narrow spectrum antibiotics and time to discharge from hospital in both treatment groups and assess the group differences via Kaplan Meier plots and Cox regression. Average utility will be compared between the two groups at 28 days using linear regression (Table 6). The ineligible/inevaluable participants will be excluded in secondary analyses (per protocol, CACE, etc.).

Sub group analysis

Several exploratory sub group analyses, including one split by the organ system of the infection (i.e. lower urinary tract, lower respiratory, intra-abdominal, bacteraemia, skin and soft tissue, etc), will be pre-specified in the Statistical Analysis Plan.

Missing, unused and spurious data

Missing primary outcome data are likely to be minimal, so complete case analysis will be used. However, if this exceeds more than 20% of participants we will employ multiple imputation and report the impact on the treatment effect alongside the complete case analysis.

Economic evaluation

For peer review only - http://bmjopen.bmj.com/site/about/guidelines.xhtml

Health economic analysis will include direct and indirect costs associated with unscheduled admissions (to ward or PICU), re-admissions, re-starting IV antibiotics, and hospital-acquired infections. Descriptive and regression analysis will be used to identify key elements of service use and cost and to explore the potential impact of baseline participant characteristics on the costs and outcomes measures. Average cost per participant will be estimated at the end of the treatment and the follow-up periods, respectively, and average cost per sub-group of patients may be explored for the same time points. Bootstrapping and missing data imputation will be done if justified. Differences in each arm will be assessed and used for the computation of an incremental cost-effectiveness ratio (ICER). We will calculate ICERs for a clinically effective outcome (fewer days on IV antibiotics with increased or equivalent safety) and the cost per IV antibiotic day avoided.

A cost-effectiveness analysis will assess possible efficiency gains. A NHS perspective will be used and relevant direct medical costs will be collected. Information on resource use will include data on inpatient bed days, antibiotic consumption, nursing and medical resources, other medicines including over the counter medicines, diagnostic and monitoring laboratory tests, GP visits and emergency visits. Direct hospital costs will be calculated by multiplying resource use with the accompanying unit costs collected from patient level data in the participating hospitals, routine NHS sources (e.g. NHS reference costs and *British National Formulary* (BNFc) and from the manufacturer of the PCT test, as appropriate. Time horizon will be 28 days, therefore there is no need to consider a discount rate. Patients' health utility will be measured using CHU9D up to day 28.

Descriptive and regression analysis will be used to identify key elements of service use and cost and to explore the potential impact of baseline participant characteristics on the costs and outcomes measures. Differences in each arm will be assessed and used for the computation of anICER. One way sensitivity analysis will be carried out in key model parameters. Probabilistic sensitivity analysis (PSA) and cost-effectiveness acceptability curves (CEACs) will be constructed. Information on direct non-medical costs, such as travelling to and from the hospitals, and indirect costs, such as parents' productivity losses, will also be collected.

For peer review only - http://bmjopen.bmj.com/site/about/guidelines.xhtml

Qualitative study

The qualitative evaluation will aim to explore the experiences and understanding of parents of children recruited into the BATCH trial (n= 10-15) about their child's condition and treatment of confirmed or suspected bacterial infection, and also to explore their views and experiences about participating in a RCT. Interviews will be conducted after the participant reaches their day 28 follow up. A purposive sample strategy will be employed to include parents from both intervention and control arms and inclusion of different site.

It will also aim to explore the views and experiences of health professionals involved in the BATCH trial (n=10-20) about participating in a RCT with a focus on acceptability of the trial, clinical equipoise, taking informed consent, and support needs of trial involvement. Interviews will be conducted at two time points (before or at the beginning and after the intervention) which will enable us to capture whether there are any changes in attitudes towards the PCT test. A purposive sample strategy will be developed to address representation from up to 5 different sites and variation in health professional role (e.g. ward nurse. consultant, research nurse etc.)

With regards to the sample size for both health professionals and parents, the qualitative researcher(s) will make pragmatic decisions along with the research team regarding when enough is known about certain themes (I.e. data saturation has occurred.

Semi-structured interviews will be undertaken face-to-face or via telephone. They will be recorded and transcribed. Data will be double coded and analysed using thematic analysis. Non-participant observation of episodes of patient care and trial delivery (of both arms) will also be carried out in some centres. Observations and field notes will enable an understanding of how the individual intervention components and delivery processes work in the real healthcare setting.

TRIAL MANAGEMENT

The trial is sponsored by University of Liverpool, coordinated by CTR, Cardiff University and hosted by Alder Hey Children's NHS Foundation Trust. The other partner organisations are

For peer review only - http://bmjopen.bmj.com/site/about/guidelines.xhtml

University of Southampton, University Hospital Southampton NHS Foundation Trust, University Hospitals Bristol NHS Foundation Trust, Oxford University Hospitals NHS Foundation Trust, Sheffield Children's NHS Foundation Trust, Lancaster University and Hull University.

Trial Management Group

The Trial Management Group (TMG) will meet monthly throughout the course of the trial and will include the chief investigator co-applicants, collaborators, trial manager, data manager, statistician and administrator. A parent representative will also attend and contribute to the design and management of the trial, as well as patient-facing materials. TMG members will be required to sign up to the remit and conditions as set out in the TMG Charter.

Trial Steering Committee

A Trial Steering Committee (TSC) consisting of an independent chairperson, two independent members and a parent representative will provide oversight of the BATCH trial. Members will be required to sign up to the remit and conditions as set out in the TSC charters and will meet at least annually.

Independent Data Monitoring Committee

An Independent Data Monitoring Committee (IDMC) will provide oversight of all matters related to patient safety and data quality. Members will be required to sign up to the remit and conditions as set out in the IDMC charter and will meet at least annually.

Ethics and Dissemination

Research Approvals

The trial was approved by the Health Research Authority and NHS Research Ethics Committee (North West Liverpool East REC reference 18/NW/0100) on 13th April 2018. The following substantial amendments were made to the trial and were communicated to all

For peer review only - http://bmjopen.bmj.com/site/about/guidelines.xhtml

trial sites: Amendment 2 (30 May 2018); Amendment 3 (13 July 2018); Amendment 5 (12 December 2018); Amendment 9 (9 August 2019); Amendment 14 (9 October 2020). The current protocol is version 5.0 dated 8 July 2020.

Dissemination Plan

Following completion of the trial, a final report will be prepared for the National Institute of Health Research (NIHR) HTA Journal series. Findings will be published in peer-reviewed journals and presented at international conferences. Nationally, we will engage with NICE, the Royal College of Paediatrics and Child Health, The British Society for Antimicrobial Chemotherapy, British Infection Society, and the British Paediatric Allergy, Immunity and Infection Group. All publications and presentations related to the trial will be authorised by the TMG in accordance with the BATCH publication policy. Where appropriate, the results of this trial can be directly implemented in the revisions of the NICE guidelines.

DISCUSSION

This trial has the potential to impact the clinical care of hospitalised children with confirmed or suspected SBI, which currently accounts for a large proportion of antibiotic use in hospitalised children. It will contribute to the development of PCT-guided antibiotic management guidelines of infections in hospitalised children, and will address the safety of shorter antibiotic courses. If shorter duration of PCT-guided antimicrobial therapy is shown to be safe and effective, this will have major implications for direct and indirect costs of childhood hospitalisations from infection. This will potentially lead to significant reductions in duration of hospitalisation and reduced antibiotic exposure, resulting in a positive impact on healthcare services and societal costs. Reduced exposure to antibiotics will, in turn, reduce AMR. This trial will demonstrate if introducing PCT-guided AMS management in the NHS is cost-effective. Reduced antibiotic use will lead to reduced hospital costs and reduced ADRs.

For peer review only - http://bmjopen.bmj.com/site/about/guidelines.xhtml

This trial is timely as it aligns with the current Department of Health Five Year action plan for antimicrobial resistance 2019 to 2024 and is a response to research recommendations from two published NICE guidance documents (DG18 and NG15).

Acknowledgements: In addition to the authors, the BATCH team comprises Helen Nabwera, Katrina Cathie, Daniel Owens, Jenny Whitbread, Zoe Gray, Zoe Oliver, Judith Evans, Sarah Jones, Sam Clarkstone, Abin Thomas, Kim Smallman and Sarah Milosevic. The authors would like to thank the research nurses, clinicians and laboratory staff for their support during the trial. They would also like to acknowledge the contribution of the Liverpool GenerationR YPAG, the TSC members, namely Ann Marie Swart, Paul Brocklehurst, Colette Smith, Paul Dark, Phil Shackley, and Kelly Chapman, and IDMC members, namely Graeme MacLennan, Danielle Horton-Taylor, Philip Howard and Alastair Sutcliffe. The CTR receives funding from Health and Care Research Wales and Cancer Research UK.

Author Contributions: EDC is the chief investigator of this trial. EDC along with JB, SNF, SP, LH, SVP, ET-J, LB-H, CM, CH, CP, JP, MP and KH led the development of the research question, study design, obtaining the funding and implementation of the protocol. CAW is the trial manager and ET-J is the senior trial manager who coordinate the operational delivery of the trial protocol and recruitment. LB-H is the lead qualitative researcher. CH and PP are the trial statisticians. DH is the data manager. All authors listed provided critical review and final approval of the manuscript.

Funding: This trial was funded by the National Institute of Health Research Health Technology Assessment (NIHR HTA) Programme, reference 15/188/42. Sponsored by University of Liverpool, Research Support Office, 2nd Floor Block D, Waterhouse Building, 3 Brownlow Street, Liverpool, L69 3GL. Contact person: Mr Neil French; Sponsor@Liverpool.ac.uk, Sponsor reference: UoL001333. The study is supported by the NIHR Clinical Research Network, NIHR Southampton Clinical Research Facility,

Disclaimer: The views expressed are those of the authors and not necessarily those of the NHS, the NIHR or the Department of Health and Social Care. Neither the Sponsor nor the Funder had any role on the study design; collection, management, analysis and

For peer review only - http://bmjopen.bmj.com/site/about/guidelines.xhtml

interpretation of data; writing of this manuscript or in the decision to submit this manuscript for publication.

Competing interests: EDC is a member of NIHR Invention for Innovation panel, member of NICE Diagnostic advisory committee April 2014-September 2020, Member of MRC DPFS panel and MRC COVID-19 Agile panel. SNF is an NIHR Senior Investigator. All other authors declare no competing interests.

Patient and public involvement: Patients and/or the public were involved in the design, or conduct, or reporting, or dissemination plans of this research. Refer to the Methods section for further details.

Patient consent for publication: Not required.

Provenance and peer review: Not commissioned; externally peer reviewed.

Word count: 5197

For peer review only - http://bmjopen.bmj.com/site/about/guidelines.xhtml

For peer review only

References

1. Singer M, Deutschman C, Seymour C, et al. The Third International Consensus Definitions for Sepsis and Septic Shock (Sepsis-3). *JAMA* 2016;315(8):801-10. doi: 10.1001/jama.2016.0287
2. Kumar A, Roberts D, Wood K, et al. Duration of hypotension before initiation of effective antimicrobial therapy is the critical determinant of survival in human septic shock. *Critical Care Medicine* 2006;34(6):1589-96.
3. Fischer J, Ramser M, Fanconi S. Use of antibiotics in pediatric intensive care and potential savings. *Intensive Care Medicine* 2000;27(7):959-66.
4. Goldmann D, Weinstein R, Wenzel R, et al. Strategies to Prevent and Control the Emergence and Spread of Antimicrobial-Resistant Microrganisms in Hospitals. A challenge to hospital leadership. *JAMA* 1996;275(3):234-40.
5. Irwin A, Grant A, Williams R, et al. Predicting Risk of Serious Bacterial Infections in Febrile Children in the Emergency Department. *Pediatrics* 2017;140(2):e20162853.
6. Carrol E, Thomson A, Hart C. Procalcitonin as a marker of sepsis. *International Journal of Microbial Agents* 2002;20(1):1-9.
7. Department of Health. Tackling Antimicrobial Resistence 2019-2024. The UK's five-year national action plan, 2019.
8. Patel S, Vergnano S. The impact of paediatric antimicrobial stewardship programmes on patient outcomes. *Curr Opin Infect Dis* 2018;31(3):216-23. doi: doi:10.1097/QCO.0000000000000449
9. Nielsen M, Baines P, Jennings R, et al. Procalcitonin, C-reactive protein, neutrophil gelatinase-associated lipocalin, resistin and the APTT waveform for the early diagnosis of serious bacterial infection and prediction of outcome in critically ill children. *PLoS One* 2021;16(2)
10. Westwood M, Ramaekers B, Whiting P, et al. Procalcitonin testing to guide antibiotic therapy for the treatment of sepsis in intensive care settings and for suspected bacterial infeaction in emergency department settings: a systematic review and ocst-effectiveness analysis. *Health Technology Assessment* 2015;19(96) doi: 10.3310/hta19960
11. McMullan B, Andersen D, Blyth C, et al. Antibiotic duration and timing of the swtich from intravenous to oral route for bacterial infections in children: systematic review and guidelines. *Lancet Infectious Diseases* 2016;16(8):e139-52. doi: 10.1016/S1473-3099(16)30024-X
12. Gallagher R, Kirkham J, Mason J, et al. Development and inter-rater reliability of the Liverpool adverse drug reaction causality assessment tool. . *PLoS One* 2011;6(12):e28096.
13. Bracken L, Nunn A, Kirkham J, et al. Development of the Liverpool Adverse Drug Reaction Avoidability Assessment Tool. *PLoS ONE* 2017;12(1):e0169393. doi: doi:10.1371/journal.pone.0169393
14. Stevens K. Valuation of the Child Health Utility 9D Index. *PharmacoEcononics* 2012;30:729–47.
15. Altman D, Bland J. Treatment allocation by minimisation. *BMJ* 2005;330:843.
16. Generation R Young People Improving Research [Available from: https://generationr.org.uk/liverpool

For peer review only - http://bmjopen.bmj.com/site/about/guidelines.xhtml

17. Gillespie D, Francis N, Carrol E, et al. Use of co-primary outcomes for trials of antimicrobial stewardship interventions. *The Lancet Infectious Diseases* 2018;18(6):595-97.
18. Paediatric Intensive Care Audit Network (PICANet). Annual Report (published November 2015), 2015.
19. Bustinduy ACS, I. Shaw, R. Irwin, A. Thiagarajan, J. Beynon, R. Ladhani, S. Sharland, M. CABIN network. Predictors of fever-related admissions to a paediatric assessment unit, ward and reattendances in a South London emergency department: the CABIN 2 study. . *Arch Dis Child* 2017;102(1):22-28. doi: doi:10.1136/archdischild-2016-310494
20. D'Souza SG, R. Jennings, R. Siner S. Paulus, S. Thorburn, K. Chesters, C. Downey, C. Baines, P. Lane, S. Carrol, ED. Procalcitonin and Other Common Biomarkers Do Not Reliably Identify Patients at Risk for Bacterial Infection After Congenital Heart Surgery. . *Pediatr Crit Care Med* 2019;20(3):243-51. doi: doi:10.1097/PCC.0000000000001826
21. Chuang-Stein C, Stryszak P, Dmitrienko A, et al. Challenge of multiple co-primary endpoints: a new approach. *Stat Med* 2007;26(6):1181-92. doi: 10.1002/sim.2604
22. NICE. Meningitis (bacterial) and meningococcal septicaemia in under 16s: recognition, diagnosis and management. Clinical guideline [CG102]. 2010
23. de Jong E, van Oers J, Beischuizen A, et al. Efficacy and safety of procalitonin guidance in reducing the duration of antibiotic treatment in critically ill patients: a randomised, controlled, open-label trial. *Lancet Infectious Diseases* 2016 doi: 10.1016/S1473-3099(16)00053-0

For peer review only

For peer review only - http://bmjopen.bmj.com/site/about/guidelines.xhtml

Table 1 Eligibility Criteria

Inclusion criteria	Exclusion criteria
• All children aged between 72 hours old and up to 18 years old admitted to hospital for confirmed or suspected severe bacterial infection (SBI), in whom intravenous (IV) antibiotics are commenced, and who are expected to remain on IV antibiotics for more than 48 hours.<br><br>Conditions include (but not limited to):<br>bacteraemia, central line-associated bloodstream infections (CLABSIs), uncomplicated bone and joint infections (such as single site infection, osteomyelitis with adjacent septic arthritis or septic arthritis with adjacent osteomyelitis), discitis, empyema, pneumonia, pyelonephritis, sinusitis, retropharyngeal abscess, pyomyositis, uncomplicated culture-negative meningitis, intra-abdominal infections, lymphadenitis, cellulitis.<br><br>• First time in the BATCH trial.	• Preterm infants age <37 weeks corrected gestational age, under 72 hours old or ≥18 years of age.<br>• Children admitted moribund and not expected to survive more than 24 hours.<br>• Children with a predicted duration of IV antibiotics of less than 48 hours.<br>• Children not expected to survive at least 28 days because of a pre-existing condition.<br>• Children with bacterial meningitis*, bacterial endocarditis, or brain abscess.<br>• Children with complicated bone and joint infections**.<br>• Children receiving antibiotics for surgical prophylaxis.<br>• Children with chronic co-morbidities, such as cystic fibrosis, chronic lung disease, bronchiectasis where there is already a pre-defined length of course of antibiotics.<br>• Children who are severely immunocompromised (e.g. chemotherapy, stem cell transplant, biological therapy for inflammatory or rheumatological conditions).<br>• Children who, in the opinion of the local investigator, are unsuitable for randomisation due to high probability of requiring sustained IV therapy.<br>• Children with a presence of existing directive to withhold life-sustaining treatment.<br>• Inborn infants admitted to Neonatal Intensive Care Units (NICU), Neonatal High Dependency Units (NHDU), Special Care Baby Units (SCBU) or Postnatal wards.<br><br>*excluded due to NICE guideline on bacterial meningitis has predefined recommendations for duration of IV antibiotics.[22]

For peer review only

	** defined as chronic and/or related to a fracture or fixation device or prosthesis or implant. Chronic osteomyelitis presents six or more weeks after bone infection and is characterised by the presence of bone destruction and formation of sequestra.

For peer review only

For peer review only - http://bmjopen.bmj.com/site/about/guidelines.xhtml

Table 2 Schedule of enrolment, interventions and assessments

Data Type		Source Data	Data type	Screening	Baseline	Post randomisation until discharged home	Follow up (Day 28)	Frequency	By whom
1	Informed Consent	Consent form	-	X				Once	Site Clinical/Research team
2	Eligibility Assessment	Eligibility Case Report Form (CRF)	-	X				Once	Site research team
3	Demographics	CRF			X			Once	Research Nurse
4	Admission data	CRF	Comorbidities, preadmission antibiotic use, initial working diagnosis		X			Once	Research Nurse
5	Health-related Quality of Life	QRF	CHU9D		X		X	Twice	Patient/parent reported (over telephone or post at Day 28)
6	Randomisation	CRF	-		X			Once	Site Research team
7	Antibiotic use (IV/Oral)	Observation charts/ Medical notes	Antibiotic type, dose, duration,			X		Daily	Research Nurse
8	Blood tests including PCT	CRF/Medical notes	Routine blood tests<br>PCT results (for those in intervention arm)			X			
9	Clinical review	CRF/Medical notes	Clinical decision made and whether the algorithm was complied with			X		As required when a clinical decision has been made	

For peer review only

**Data Type**		**Source Data**	**Data type**	**Screening**	**Baseline**	**Post randomisation until discharged home**	**Follow up (Day 28)**	**Frequency**	**By whom**
10	Cerebrospinal fluid metrics, radiology and microbiology	CRF/Medical notes	White cell count, biochemistry. Microbiology results, radiology results			X			
11	Re-commencing of Antibiotics (IV and oral	Observation charts/ Medical notes	Antibiotic type, dose, duration, time recommenced			X		Daily	Research Nurse
12	Unscheduled Admissions	Medical notes	PICU readmissions post discharge			X		Daily	Research Nurse
13	Mortality	Medical notes	Date, description			X		If before Day 28	Research Nurse
14	Discharge	Medical notes	Date, description			X		If before Day 28	Research Nurse
15	Adverse events	Observation charts/ Medical notes	Date, type			X		Daily	Research Nurse
16	Suspected Adverse Drug Reactions (ADR)	Liverpool Causality Assessment tool	Date, description			X		Daily	Research Nurse
17	Resource Use	Questionnaire	Direct Medical costs (Inc. medication and ventilation and vasopressor) and Resource Use				X	Once	Patient/parent reported (over telephone or post)
18	SAE	SAE form			←------------------------As Required----------------------→				Research Nurse
19	Withdrawals	Withdrawal form			←------------------------As Required----------------------→				Research Nurse, Centre for Trial Research

For peer review only

Table 3 Elements of the Composite Safety Outcome

Composite element	Definition	Reason for inclusion	Expected prevalence in usual care	Potential direction of change with intervention
Unscheduled admissions/ Readmissions	Admitted/readmitted to PICU or unplanned readmission to hospital within 7 days of stopping IV antibiotics	Indicators of a deterioration and need for increased level of care	Our observation study showed 8.8% patients have admissions/re-admissions[5]	Increase
Reinstating IV antibiotic therapy	Restarting IV antibiotic (for any reason) therapy within 7 days of stopping IV therapy	Indicator of potentially inappropriate withdrawal of IV antibiotics and deterioration	de Jong study 2.9% in control arm re-started IV antibiotic[23]	Increase
Mortality	Death for any reason in the 28 days following randomisation	-	PICANet Annual report 2015: deaths on PICUs ~4% in 2012-2014[18]	Increase

For peer review only - http://bmjopen.bmj.com/site/about/guidelines.xhtml

Table 4 Combined Primary Outcome

	Antibiotic duration different (reduction in PCT group) (H1)	Antibiotic duration no different (no reduction in PCT group) H0)
Safety composite not worse in PCT group (H1)	✔	✖
Safety composite worse in PCT group (H0)	✖	✖

✔ - Intervention successful; ✖ - Intervention unsuccessful

Table 5 Summary of analyses of co-primary outcomes

	Co-primary outcomes	Analysis approach	Covariates in the model
Primary analysis	Duration of days of IV antibiotics (Intervention effect)	Cox regression (superiority test)	Trial arm and minimisation factors (site, age group)
	Adverse events (composite safety outcome)	Logistic regression (non-inferiority test)	Trial arm and minimisation factors (site, age group)
Secondary analyses	Duration of days of IV antibiotics (Intervention effect)	Kaplan Meier plot	Trial arm
		Log rank test	Trial arm
		Cox regression (assessments of suspected baseline confounders)	Covariates in the primary analysis, plus suspected baseline confounders (e.g. gender)
		Complier Average Causal Effect (CACE)	Covariates in the primary analysis, plus intervention adherence
	Adverse events (composite safety outcome)	Logistic regression (assessments of suspected baseline confounders)	Covariates in the primary analysis, plus suspected baseline confounders (e.g. gender)

Table 6 Summary of analyses of secondary outcomes

Secondary outcomes	Analysis approach	Covariates in the model
Proportion of ADR	Logistic regression	Trial arm and minimisation factors (site, age group)
Proportion of HAI		
Proportion of unscheduled readmission		
Proportion of re-commencing IV antibiotics		
Proportion of mortality		
Duration of antibiotics (IV and oral)	Cox regression	Trial arm and minimisation factors (site, age group)
Time to switch from broad to narrow spectrum antibiotics		
Time to discharge from hospital		
Average utility	Linear regression	Trial arm and minimisation factors (site, age group)

For peer review only - http://bmjopen.bmj.com/site/about/guidelines.xhtml

Figure 1 Guidelines for continuing or stopping IV antibiotics

Figure 2 Trial Schema / Participant flow

For peer review only

For peer review only - http://bmjopen.bmj.com/site/about/guidelines.xhtml

Figure 1 Guidelines for continuing or stopping IV antibiotics

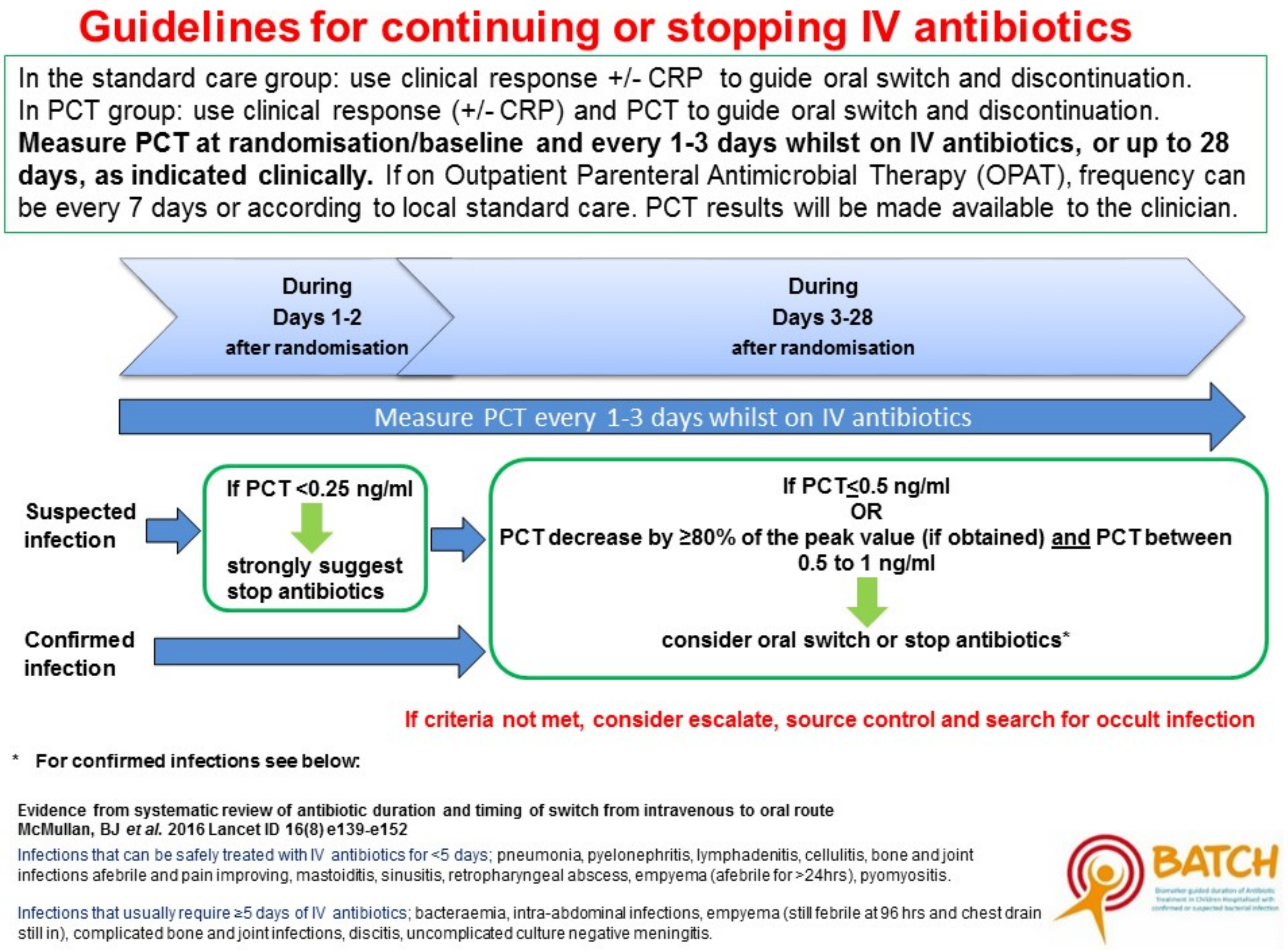

Figure 2 Trial Schema / Participant flow

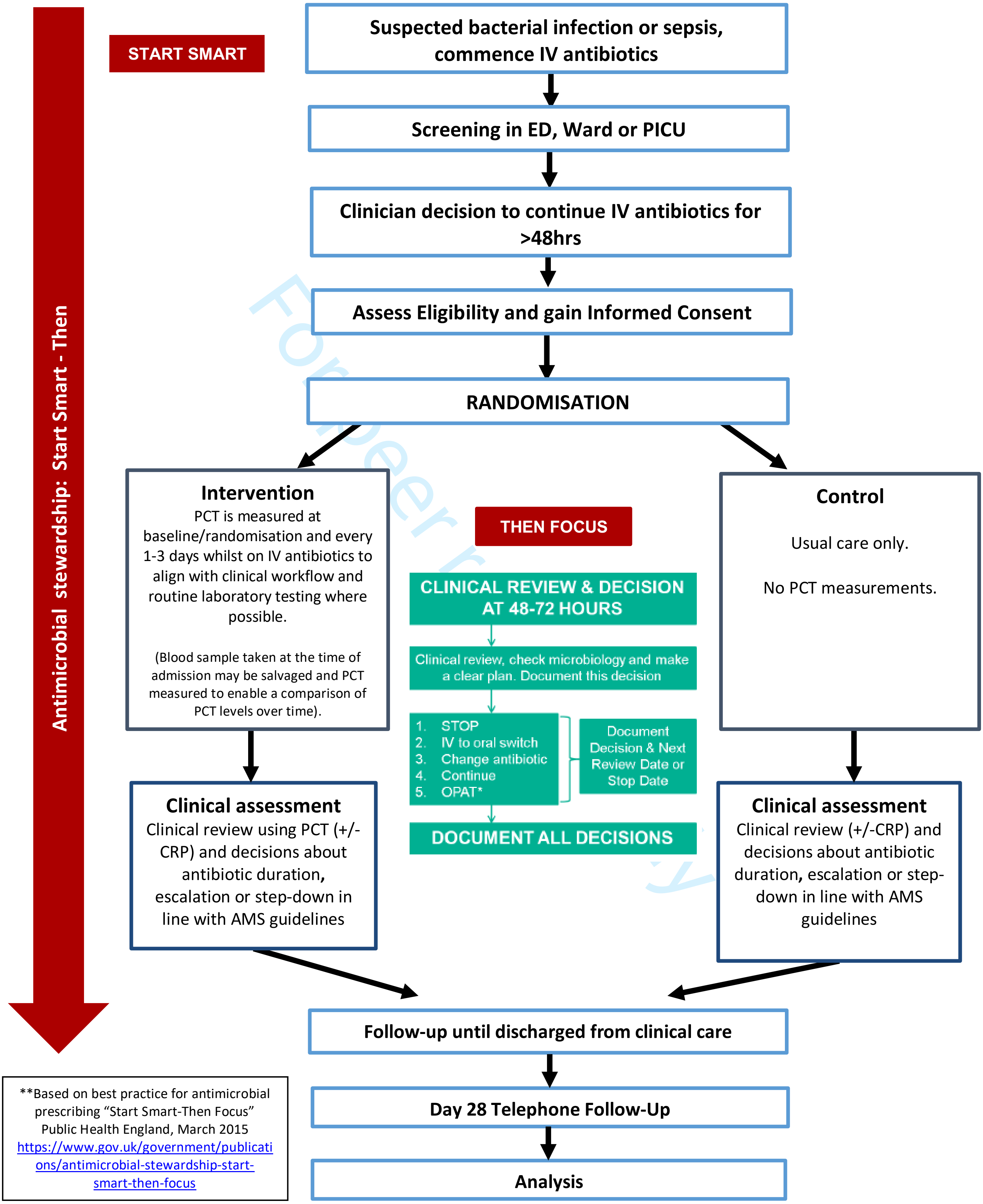

Insert NHS Trust / Health Board logo

Biomarker-guided duration of Antibiotic Treatment in Children Hospitalised with confirmed or suspected bacterial infection Trial

Information Sheet for Parents & Guardians

We are inviting you and your child to take part in this research trial run by Doctors at your hospital and scientists at the University of Liverpool, as well as some researchers from Cardiff University, Lancaster University and the University of Southampton. Before you decide if you want your child to take part, we would like you to understand why the research is being done and what it would involve for you and your child.

Please read this information sheet carefully. One of our team can go through the information sheet with you and will answer any questions you have.

What is the study about?

A daily task in hospitals is to assess ill children for the presence or absence of infection. A decision has to be made as to whether to start, stop or change antibiotics. If we can reduce antibiotic usage within hospitals, this would be an important step in combating the spread of hospital superbugs.

We are interested in studying bacterial infections in children and young people admitted to hospital. We will be assessing different markers of bacterial infection in children in hospital, to see if they can help us identify how children and young people are responding to the antibiotics that are being used to treat the infection.

For peer review only - http://bmjopen.bmj.com/site/about/guidelines.xhtml

Procalcitonin is a blood test which measures the body's response to bacterial infection, so the level is high if you have a bacterial infection, but levels reduce as you respond to antibiotics.

We will be using procalcitonin to help us decide if a patient is responding to antibiotic treatment or whether the treatment can be stopped safely.

We want to see if the use of a procalcitonin test can safely aid decisions to start, stop or change antibiotics. We are looking to recruit nearly two thousand patients aged under 18 years old to take part in a trial to help us reduce antibiotic usage safely in children and young people admitted to hospital.

We need to assess new tests of infection in a scientific manner to be sure that they improve patient care before being introduced into everyday routine practice.

Why has my child been asked to take part?

Your child's clinician thinks that your child may have a bacterial infection, and has identified that they could be eligible for the BATCH trial.

Does my child have to take part?

Your child does not have to take part in this trial. Participation in the study is entirely voluntary and you and your child are free to refuse to take part or withdraw from the trial at any time without having to give a reason. If you do decide not to take part or to withdraw from the trial, this will not affect the care that your child receives.

What will happen if my child takes part?

If you do decide to take part in the trial your child will be randomised to either have procalcitonin tests or normal care. We will try to do these procalcitonin tests at the same time as routine blood tests are being taken, however, we may need to take an additional blood test at separate time points if routine bloods are not due to be collected or there is not enough routine blood collected to perform the procalcitonin test,

There will be an equal chance of your child having the additional procalcitonin tests or receiving normal care.

For peer review only - http://bmjopen.bmj.com/site/about/guidelines.xhtml

Can I choose which part of the trial to be in?

No. To get the most reliable information about how well the procalcitonin test works we must ensure that each patient is given an equal chance of having the additional tests or not, and that this is chosen at random.

During your child's hospital stay

Your child's clinician will manage your child's care as per normal practice, however, if your child is randomised to the intervention group and has the procalcitonin tests, your child's treatment will be guided by the results of these tests. We will study how your child's procalcitonin levels relate to their response to treatment.

We will collect an additional 1ml of blood for this test. We will try to collect this when blood is being taken as part of routine care, however we may need to take an additional blood test at separate time points if routine bloods are not due to be collected or there is not enough routine blood collected to perform the procalcitonin test. All the other tests performed are those which should routinely be performed.

We will collect information on:

- whether infection is present and on the severity of their illness
- costs associated with having a child in hospital with a bacterial infection
- You will also be asked to complete some questionnaires about your child's health status.

If you do not give consent to take part in the study this will not affect the care given to your child.

What will happen to the blood sample?

Your child's samples taken whilst they are being treated for this illness will be tested in accredited laboratories at your hospital. They will be kept safe and will only be identified by a study code.
This may include samples taken when they were admitted which are normally discarded by the laboratory after the routine tests are done.

Your child's blood samples taken whilst they are being treated for this illness may also be stored for future research concerning infections and antibiotic resistance.

For peer review only - http://bmjopen.bmj.com/site/about/guidelines.xhtml

The samples will be stored **confidentially** in a secure location in a laboratory at the University of Liverpool. Your child's name will not be stored with the sample and will not be used in any publications relating to use of your child's sample(s). **You may withdraw consent for this storage and future use of your child's sample(s)**. If you withdraw consent, the sample(s) will not be used in any future studies and will be destroyed according to approved practices. We will seek ethical approval if we want to use the samples for any future research. We will not be doing any tests on human DNA now or in the future.

At Day 28 follow-up.

We will contact you 28 days after your child was recruited into the study to find out how they are. This will likely be by telephone, email or post and should take around half an hour of your time. We would also like to look at your child's medical records so that we can collect information on healthcare costs such as prescriptions, visits to the GP and so on. This is to allow us to work out if the additional test to reduce antibiotic use will save money in the NHS in the long run.

Longer term follow up using health data

After the trial, we would like to see how your child is doing in the longer term by using routinely collected health data. This is information that your GP and hospital collect when looking after your child. We will look at this information to explore the long term effects of procalcitonin test versus normal care.

The reason we want to look at this information is to assess your child's well-being and health following the trial. The research team will provide details such as name, NHS number, date of birth, postcode and gender to identify your child to NHS Digital in order for those professionals to provide relevant information about your child's ongoing health. NHS Digital will provide information on how trial participants have used different health services (e.g. how many hospital visits) and for what reasons.

NHS digital will provide data to Cardiff University. No identifiable data will be sent to the database. Instead, a study number will be assigned to each individual and this will be used to join pieces of information together. Data viewed by the research team will not be identifiable. In other words, when we look at your health information, all we will see is a database containing numbers. A Data Manager will use the study number to identify a person and join up the information. We will not know who is who in the database. The data will be held on a secure server and only the research team will have access to it.

For peer review only - http://bmjopen.bmj.com/site/about/guidelines.xhtml

Qualitative interview study

A few parents in the trial will be asked by a member of the research team to discuss the experience of their child taking part in the trial and about their child's health. If you agree, a researcher will give you some information about the additional study and what will be required. After you have had time to go through this and ask any questions you might have, you will be asked if you are happy to take part. If so you will be asked to sign a consent form to confirm that you understand this part of the study and agree to participate. If you do not take part this will not affect your involvement in the research or ongoing health care.

What are the possible advantages of taking part?

If the number of days that it is necessary to give antibiotic treatment can be safely reduced, children may benefit from the possibility of fewer days suffering from the known side effects of antibiotic treatment and a reduced risk of building up resistance to antibiotics working in the future (antimicrobial resistance). Your child will continue to receive standard clinical treatment, and their clinicians continue to decide on how long antibiotic treatment is given. By participating in this trial the information we collect about your child will help children/young people in the future.

What are the possible disadvantages and risks of taking part?

Taking part in the trial will mean that we ask you to give up some of your time during your child's hospital stay and at your child's follow-up telephone call. There are no risks for your child. In the event that something does go wrong and your child is harmed during the research and this is due to someone's negligence then you may have grounds for a legal action for compensation against Alder Hey Children's NHS Foundation Trust and The University of Liverpool, but you may have to pay your legal costs. The normal National Health Service complaints mechanisms will still be available.

Will all my information be kept confidential?

Yes, all information collected about you and your child during the course of the trial will be kept strictly confidential. Doctors involved in the care of your child will be informed about your participation in the trial, and will be kept up to date with your child's progress to ensure their safety, but they will not see any answers to trial questionnaires.

For peer review only - http://bmjopen.bmj.com/site/about/guidelines.xhtml

Your child’s medical records may be examined by the research team but will be kept confidential. Your child’s name (or any other identifiable information) will not be given out or appear on any publications**.**

University of Liverpool and Cardiff University will use your name, date of birth and contact details to contact you about the research study, and make sure that relevant information about the study is recorded for your care, and to oversee the quality of the study. Individuals from University of Liverpool and Cardiff University and regulatory organisations may look at your research records to check the accuracy of the research study. The only people in University of Liverpool and Cardiff University who will have access to information that identifies you/your child will be people who need to contact you to collect more information, take part in a qualitative interview, send you the results of the trial or to audit the data collection process. The people who analyse the information will not be able to identify you and will not be able to find out your name, or contact details.

University of Liverpool and Cardiff University will keep identifiable information about you/your child securely, for a minimum of 10 years after the BATCH trial has finished. This is in line with University of Liverpool policies.

All members of the trial team and regulatory authorities are trained in data protection issues. They are also bound by the terms of the General Data Protection Regulation (GDPR) (EU) 2016/679.

Who is responsible for looking after my information?

University of Liverpool is the sponsor for this study based in the United Kingdom. The University of Liverpool and Cardiff University will be using information from you and/your child’s medical records in order to undertake this study and will act as the data controller for this study. This means that we are responsible for looking after your information and using it properly. Your rights to access, change or move your information are limited, as we need to manage your information in specific ways in order for the research to be reliable and accurate. If you withdraw from the study, we will keep the information about you/ your child that we have already obtained. To safeguard your rights, we will use the minimum personally-identifiable information possible.

You can find out more about how we use your information here:
https://www.cardiff.ac.uk/public-information/policies-and-procedures/data-protection

For peer review only - http://bmjopen.bmj.com/site/about/guidelines.xhtml

Is my data likely to be used for future research?

When you agree to take part in a research study, the information about your health and care may be provided to researchers running other research studies in this organisation and in other organisations. These organisations may be universities, NHS organisations or companies involved in health and care research in this country or abroad. Your information will only be used by organisations and researchers to conduct research in accordance with the UK Policy Framework for Health and Social Care Research.

This information will not identify you/your child and will not be combined with other information in a way that could identify you. The information will only be used for the purpose of health and care research, and cannot be used to contact you or to affect your care. It will not be used to make decisions about future services available to you, such as insurance.

For peer review only

What if I, or my child do not want to carry on being part of the trial?

You can decide for your child to stop taking part in the trial at any time and without needing to give a reason. If you wish to withdraw, you can contact the trial manager or let the research nurse know next time they contact you. In order for us to understand the reasons why parents withdraw their children from the study, we may ask you why you have decided to withdraw. However, you do not have to give any reasons.

It is usual practice to keep the information we have collected to help draw conclusions about the treatment you received. If however you do not want your information used you may request that it be withdrawn from the study as well.

If you decide to stop taking part, your child's current and future medical care and the legal rights of you and your child will not be affected in any way.

What will happen to the results of the research?

We hope that the results of the trial will improve the care of many patients in the future. At the end of the trial, the results will be published in medical journals and presented at medical conferences. This will allow us to tell other doctors and healthcare professionals across the world about the results of the trial.

For peer review only - http://bmjopen.bmj.com/site/about/guidelines.xhtml

Information that identifies you or your child **<u>will not</u>** be presented. We can provide you with information of where to access the published trial data. A copy of the results can be sent to you if you would like.

Who has funded and approved the study?

This trial has been funded by the National Institute for Health Research Health Technology Assessment (NIHR HTA) Programme and is being managed by Cardiff University.

The study has been approved by an independent NHS Research Ethics Committee (REC) North West-Liverpool East (reference: 18/NW/0100). The committee makes sure that the study is conducted ethically to protect the safety, rights, wellbeing and dignity of the patient in line with the requirements of Clinical Trial Regulations.

What if there is a problem?

If your child experiences any side effects or complications as a result of taking part in the trial there are no compensation arrangements over and above the usual routes available to any patient treated within the NHS. If at any point you are unhappy with any aspect of the trial, please advise your child's Clinician or the research team at the Centre for Trials Research, Cardiff University (contact numbers below). If you remain unhappy and wish to formally complain, you can do this through the normal NHS complaints procedure. Taking part in the trial will not affect your legal rights.

What do I need to do <u>now</u>?

If you agree to consider having your child take part in the study please tell your child's clinician whether or not you are happy for your child to take part. If your child is old enough you will probably want to discuss the study with them. The research nurse can help you discuss the study with your child.

Thank you for reading this information sheet and considering participation in this trial**.** Our team is experienced and dedicated to doing this important trial to the highest international standards, and helping to improve the future care of patients.

For peer review only - http://bmjopen.bmj.com/site/about/guidelines.xhtml

Should you have any further questions or require further information about taking part you can contact (during normal working hours):

BATCH Trial Manager
Centre for Trials Research,
Cardiff University,
Heath Park,
Cardiff, CF14 4YS
Tel: 02920 687609
Email: BATCH@cardiff.ac.uk

Please note that this number is only for queries regarding the trial; if you have an urgent medical problem please contact your surgeon/doctor in the normal way.

The Principal Investigator for this site is:-

Insert PI details

The Research Nurse for this site is:-

Insert RN detail

For peer review only

For peer review only - http://bmjopen.bmj.com/site/about/guidelines.xhtml

BATCH: Biomarker-guided duration of Antibiotic Treatment in Children Hospitalised with confirmed or suspected bacterial infection Trial

Insert NHS Trust /Health Board logo

Site ID | | |

Patient ID | | | |

CONSENT FORM

(Please **initial** each box and sign in full at the bottom of the page)

1. I confirm that I have read and understood the Information Sheet Information Sheet for Parents and Guardians (version 2.1, dated 31/01/2019) for the BATCH trial. I have had the opportunity to consider the information, ask questions and have had these answered satisfactorily. ☐
2. I understand that my child's participation is voluntary and that I am free to withdraw my child at any time, without giving any reason, without my child's normal medical care or legal rights being affected. ☐
3. I understand the trial is randomised and no one can pick which treatment my child receives. I understand that my child will be randomised to have either procalcitonin tests or usual care. If my child is allocated to the treatment arm of the trial their procalcitonin levels will be tested. This may be done as part of routine blood tests or an additional blood sample may need to be taken at separate time points if routine bloods are not due to be collected or not enough routine blood is collected to perform the procalcitonin test. I consent to these tests being performed on the samples collected during my child's illness. I understand that I will not be given the laboratory results. ☐
4. I understand that the research team may be required to look in my child's medical notes or contact my child's GP to ask about my child's health and medications. I give permission for them to do this. ☐
5. I understand that a member of the research team will need to contact me by telephone, email or post to complete a survey about my child's health. I give permission for them to do so. ☐
6. I understand that a member of the research team may need to contact me to carry out an interview about my child's health and experiences in the trial (Qualitative Interviews). I give permission for them to do so. ☐
7. I understand that information collected about my child that is held and maintained by NHS Digital and other central UK NHS bodies, may be collected from my medical records and other health-related records and looked at by the research team and responsible practitioners during the trial. I give permission for these individuals to have access to these records and for them to be used in this research on the understanding that all information will remain confidential. ☐
8. I understand that information collected about my child (including name and address) will be held at the Centre for Trials Research, Cardiff University according to the 2018 General Data Protection Regulation (GDPR) (EU 2016/679). I understand that this information will be kept strictly confidential and that no personal information will be used in the study report or publications. ☐
9. I understand that my child's blood samples will be stored securely for further research. Names and addresses will be removed from all samples stored for research purposes. Further ethical approval will be sought from the Ethics Committee for any future research on these samples. No DNA analysis will be carried out now or in the future. ☐
10. I agree for my child to take part in the above trial. ☐

Name of Child (please print):____

Name of Parent / Guardian (please print):______________________ Signed:__________Date:___/___/___

Name of Person taking consent (please print):____________________Signed:__________Date:___/___/___

When completed, store White copy in Site File; Green for Medical Notes; Yellow for parent/carer
Please fax a copy to CTR: 02030 095405

Local Researcher: Professor Enitan Carrol, Honorary Consultant in Paediatric Infectious Diseases and Immunology, University of Liverpool and Alder Hey Children's NHS Foundation Trust

For peer review only - http://bmjopen.bmj.com/site/about/guidelines.xhtml

Reporting checklist for protocol of a clinical trial.

Based on the SPIRIT guidelines.

Instructions to authors

Complete this checklist by entering the page numbers from your manuscript where readers will find each of the items listed below.

Your article may not currently address all the items on the checklist. Please modify your text to include the missing information. If you are certain that an item does not apply, please write "n/a" and provide a short explanation.

Upload your completed checklist as an extra file when you submit to a journal.

In your methods section, say that you used the SPIRITreporting guidelines, and cite them as:

Chan A-W, Tetzlaff JM, Gøtzsche PC, Altman DG, Mann H, Berlin J, Dickersin K, Hróbjartsson A, Schulz KF, Parulekar WR, Krleža-Jerić K, Laupacis A, Moher D. SPIRIT 2013 Explanation and Elaboration: Guidance for protocols of clinical trials. BMJ. 2013;346:e7586

		Reporting Item	Page Number
**Administrative information**			
Title	#1	Descriptive title identifying the study design, population, interventions, and, if applicable, trial acronym	1
Trial registration	#2a	Trial identifier and registry name. If not yet registered, name of intended registry	1
Trial registration: data set	#2b	All items from the World Health Organization Trial Registration Data Set	1
Protocol version	#3	Date and version identifier	17
Funding	#4	Sources and types of financial, material, and other support	19
Roles and responsibilities: contributorship	#5a	Names, affiliations, and roles of protocol contributors	1 and 19

For peer review only

Roles and responsibilities: sponsor contact information	#5b	Name and contact information for the trial sponsor	19
Roles and responsibilities: sponsor and funder	#5c	Role of study sponsor and funders, if any, in study design; collection, management, analysis, and interpretation of data; writing of the report; and the decision to submit the report for publication, including whether they will have ultimate authority over any of these activities	19
Roles and responsibilities: committees	#5d	Composition, roles, and responsibilities of the coordinating centre, steering committee, endpoint adjudication committee, data management team, and other individuals or groups overseeing the trial, if applicable (see Item 21a for data monitoring committee)	17
**Introduction**			
Background and rationale	#6a	Description of research question and justification for undertaking the trial, including summary of relevant studies (published and unpublished) examining benefits and harms for each intervention	2-5
Background and rationale: choice of comparators	#6b	Explanation for choice of comparators	7
Objectives	#7	Specific objectives or hypotheses	5
Trial design	#8	Description of trial design including type of trial (eg, parallel group, crossover, factorial, single group), allocation ratio, and framework (eg, superiority, equivalence, non-inferiority, exploratory)	6
**Methods: Participants, interventions, and outcomes**			
Study setting	#9	Description of study settings (eg, community clinic, academic hospital) and list of countries where data will be	6

For peer review only

		collected. Reference to where list of study sites can be obtained	
Eligibility criteria	#10	Inclusion and exclusion criteria for participants. If applicable, eligibility criteria for study centres and individuals who will perform the interventions (eg, surgeons, psychotherapists)	6 and Table 1
Interventions: description	#11a	Interventions for each group with sufficient detail to allow replication, including how and when they will be administered	7
Interventions: modifications	#11b	Criteria for discontinuing or modifying allocated interventions for a given trial participant (eg, drug dose change in response to harms, participant request, or improving / worsening disease)	n/a
Interventions: adherance	#11c	Strategies to improve adherence to intervention protocols, and any procedures for monitoring adherence (eg, drug tablet return; laboratory tests)	n/a
Interventions: concomitant care	#11d	Relevant concomitant care and interventions that are permitted or prohibited during the trial	n/a
Outcomes	#12	Primary, secondary, and other outcomes, including the specific measurement variable (eg, systolic blood pressure), analysis metric (eg, change from baseline, final value, time to event), method of aggregation (eg, median, proportion), and time point for each outcome. Explanation of the clinical relevance of chosen efficacy and harm outcomes is strongly recommended	7-8
Participant timeline	#13	Time schedule of enrolment, interventions (including any run-ins and washouts), assessments, and visits for participants. A schematic diagram is highly recommended (see Figure)	10-11 and Figure 2
Sample size	#14	Estimated number of participants needed to achieve study objectives and how it was determined, including clinical and statistical assumptions supporting any sample size calculations	12
Recruitment	#15	Strategies for achieving adequate participant enrolment to reach target sample size	9

For peer review only

**Methods: Assignment of interventions (for controlled trials)**			
Allocation: sequence generation	#16a	Method of generating the allocation sequence (eg, computer-generated random numbers), and list of any factors for stratification. To reduce predictability of a random sequence, details of any planned restriction (eg, blocking) should be provided in a separate document that is unavailable to those who enrol participants or assign interventions	10
Allocation concealment mechanism	#16b	Mechanism of implementing the allocation sequence (eg, central telephone; sequentially numbered, opaque, sealed envelopes), describing any steps to conceal the sequence until interventions are assigned	10
Allocation: implementation	#16c	Who will generate the allocation sequence, who will enrol participants, and who will assign participants to interventions	10
Blinding (masking)	#17a	Who will be blinded after assignment to interventions (eg, trial participants, care providers, outcome assessors, data analysts), and how	13
Blinding (masking): emergency unblinding	#17b	If blinded, circumstances under which unblinding is permissible, and procedure for revealing a participant's allocated intervention during the trial	n/a
**Methods: Data collection, management, and analysis**			
Data collection plan	#18a	Plans for assessment and collection of outcome, baseline, and other trial data, including any related processes to promote data quality (eg, duplicate measurements, training of assessors) and a description of study instruments (eg, questionnaires, laboratory tests) along with their reliability and validity, if known. Reference to where data collection forms can be found, if not in the protocol	10-11 and Table 2

For peer review only

Data collection plan: retention	#18b	Plans to promote participant retention and complete follow-up, including list of any outcome data to be collected for participants who discontinue or deviate from intervention protocols	11
Data management	#19	Plans for data entry, coding, security, and storage, including any related processes to promote data quality (eg, double data entry; range checks for data values). Reference to where details of data management procedures can be found, if not in the protocol	9
Statistics: outcomes	#20a	Statistical methods for analysing primary and secondary outcomes. Reference to where other details of the statistical analysis plan can be found, if not in the protocol	13
Statistics: additional analyses	#20b	Methods for any additional analyses (eg, subgroup and adjusted analyses)	14
Statistics: analysis population and missing data	#20c	Definition of analysis population relating to protocol non-adherence (eg, as randomised analysis), and any statistical methods to handle missing data (eg, multiple imputation)	14
**Methods: Monitoring**			
Data monitoring: formal committee	#21a	Composition of data monitoring committee (DMC); summary of its role and reporting structure; statement of whether it is independent from the sponsor and competing interests; and reference to where further details about its charter can be found, if not in the protocol. Alternatively, an explanation of why a DMC is not needed	17
Data monitoring: interim analysis	#21b	Description of any interim analyses and stopping guidelines, including who will have access to these interim results and make the final decision to terminate the trial	13
Harms	#22	Plans for collecting, assessing, reporting, and managing solicited and spontaneously reported adverse events and other unintended effects of trial interventions or trial conduct	12

For peer review only

Auditing	#23	Frequency and procedures for auditing trial conduct, if any, and whether the process will be independent from investigators and the sponsor	9
**Ethics and dissemination**			
Research ethics approval	#24	Plans for seeking research ethics committee / institutional review board (REC / IRB) approval	17
Protocol amendments	#25	Plans for communicating important protocol modifications (eg, changes to eligibility criteria, outcomes, analyses) to relevant parties (eg, investigators, REC / IRBs, trial participants, trial registries, journals, regulators)	17
Consent or assent	#26a	Who will obtain informed consent or assent from potential trial participants or authorised surrogates, and how (see Item 32)	9
Consent or assent: ancillary studies	#26b	Additional consent provisions for collection and use of participant data and biological specimens in ancillary studies, if applicable	9
Confidentiality	#27	How personal information about potential and enrolled participants will be collected, shared, and maintained in order to protect confidentiality before, during, and after the trial	9
Declaration of interests	#28	Financial and other competing interests for principal investigators for the overall trial and each study site	19
Data access	#29	Statement of who will have access to the final trial dataset, and disclosure of contractual agreements that limit such access for investigators	n/a
Ancillary and post trial care	#30	Provisions, if any, for ancillary and post-trial care, and for compensation to those who suffer harm from trial participation	n/a
Dissemination policy: trial results	#31a	Plans for investigators and sponsor to communicate trial results to participants, healthcare professionals, the public, and other relevant groups (eg, via publication, reporting in results databases, or other data sharing arrangements), including any publication restrictions	18

For peer review only

Dissemination policy: authorship	#31b	Authorship eligibility guidelines and any intended use of professional writers	18
Dissemination policy: reproducible research	#31c	Plans, if any, for granting public access to the full protocol, participant-level dataset, and statistical code	n/a
**Appendices**			
Informed consent materials	#32	Model consent form and other related documentation given to participants and authorised surrogates	Supplementary material
Biological specimens	#33	Plans for collection, laboratory evaluation, and storage of biological specimens for genetic or molecular analysis in the current trial and for future use in ancillary studies, if applicable	7

Notes:

- 10: 6 and Table 1
- 13: 8-9 and Figure 2
- 18a: 8-9 and Table 2 The SPIRIT checklist is distributed under the terms of the Creative Commons Attribution License CC-BY-ND 3.0. This checklist was completed on 29. November 2020 using https://www.goodreports.org/, a tool made by the EQUATOR Network in collaboration with Penelope.ai

For peer review only
